# Animal Toxicology Studies on the Male Reproductive Effects of 2,3,7,8-Tetrachlorodibenzo-p-Dioxin: Data Analysis and Health Effects Evaluation

**DOI:** 10.3389/fendo.2021.696106

**Published:** 2021-11-03

**Authors:** Tongtong Zhang, Xiang Zhou, Xiaohan Ren, Xu Zhang, Jiajin Wu, Shangqian Wang, Zengjun Wang

**Affiliations:** ^1^ Department of Urology, The First Affiliated Hospital of Nanjing Medical University, Nanjing, China; ^2^ State Key Laboratory of Reproductive Medicine, Nanjing Medical University, Nanjing, China; ^3^ Department of Urology, The First People’s Hospital of Xuzhou City, Xuzhou, China

**Keywords:** reproductive toxicity, semen parameter, dioxin, environmental pollutant, meta-analysis

## Abstract

2,3,7,8-Tetrachlorodibenzo-p-dioxin (TCDD) is a well-known environmental poison that exist in the environment for many years. However, its effect on the male reproductive system has not been clearly stated. We conducted a meta-analysis of the effect of TCDD on the male reproductive system of rodents about TCDD. Results showed that that TCDD exposure reduced the testis weight (weighted mean difference [WMD]: −0.035, 95% confidence interval [CI]: −0.046 to −0.025), sperm count (WMD: −35, 95% CI: −42.980 to −27.019), and blood testosterone concentration (WMD: −0.171, 95% CI: −0.269 to −0.073). According to our research results, TCDD can cause damage to the male reproductive system of rodents through direct or indirect exposure. In order to further explore the potential hazards of TCDD to humans, more human-related research needs to be carried out.

## 1 Introduction

2,3,7,8-Tetrachlorodibenzo-p-dioxin (TCDD) is characterized by white crystals or tan crystalline powder. TCDD is a highly toxic substance to mammals. Numerous studies suggested that the exposure to TCDD enhances the incidence rate of several kinds of malignant neoplasms in humans, and WHO declared that TCDD is a human carcinogen (NCBI, PubChem Database). TCDD is a dioxin-like compound and considered the most persistent and the most potent endocrine disruptor among other dioxin-like congeners (1, 2). Among the 210 congeners, TCDD is considered to be the most toxic (3). As an unwanted byproduct produced during the synthesis of chlorinated hydrocarbons, manufacturing pesticides, burning household waste, and forest fires, TCDD is found in the soil, air, water, and in daily foods, like fish, meat, and dairy products (4, 5). Given its extreme resistance to degradation, high lipophilicity, and extreme stability (half-life in humans up to 7–9 years), TCDD accumulates in soil and water, enters the food chain, and ingested by humans (2, 6). TCDD has gotten its great disrepute from the use of Agent Orange, a herbicide applied during the Vietnam War (August 1965 to February 1971), of which TCDD is a side product. Another famous public safety incidence has occurred in July 1976, when a chemical plant has exploded near Seveso, Italy, known as Seveso Disaster, and exposed local residents to the highest known levels of TCDD, which has resulted in high incidence of tooth malformation, low semen quality, and dysregulated cell immunity (6).

TCDD is a recognized environmental poison. The sources of TCDD include human industrial production activities and natural processes, such as volcanic eruptions and forest fires. TCDD has been identified as a human carcinogen and can increase the incidence of many malignant tumors. Recently, researchers have begun to pay attention to the possible damage of TCDD to the human reproductive system (https://www.who.int/news-room/fact-sheets/detail/dioxins-and-their-effects-on-human-health). Considering the insufficient evidence in human research, the effect of TCDD on the human male reproductive system remains unknown. A systematic quantitative analysis of the existing animal research data is conducted to provide indirect evidence for anthropological research.

Nowadays, male reproductive disorders have been well noticed and become a worldwide problem. The sperm counts in young men significantly decline over the past few years, and the rates of male reproductive cancer are on the rise in many countries (7). The incidence of testicular cancer, cryptorchidism, and hypospadias continues to increase. By contrast, the quality of semen is declining (8). In 1938–1990, the sperm concentration has decreased nearly thrice with increasing genitourinary abnormalities, such as testicular malignancy and hypospadia (9). According to the research of Agarwal, A., et al., at least 30,000,000 men around the world are suffering from infertility (10). Current studies suggested that environmental pollutant exposures of the fetal testis and their toxicity effect on the adult endocrine system may act as the foremost factors in the observed phenomena (11). With the fast development of industry and civilization, environmental toxin-induced male reproductive disorders are becoming prominent.

The potential adverse effects of TCDD on males have been investigated by human epidemiological studies and nonhuman animal studies (6, 12–16). The gestational exposure of TCDD is reported to decrease the body weight of male offspring, but a 30-year cohort study found no significant association between male offspring birthweight changes and *in utero* TCDD exposure (16–18). A dosage of 0.7 µg/kg TCDD exposure on gestational day 15 reduces the anogenital distance (AGD) of male offspring on postnatal days 1 and 4, whereas 1 µg/kg TCDD exposure on gestational day 15 shows no apparent effect on male AGD on postnatal day 1 in another study (15, 19). The oral dosage of 1 µg/kg TCDD is reported to induce low testes weight, but these results are still inconsistent with other studies (20, 21). Considering that the conclusions of animal studies are not consistent and that the evidence of human studies is inadequate, we decided to conduct a systematic review with meta-analysis to synthesize the outcomes of animal experiments to arouse people’s warnings and provide ideas for the future in-depth research of TCDD or related materials.

## 2 Methods

### 2.1 Topic Statement and Problem Formulation

The topic statement and Population, Exposure, Comparator, and Outcome (PECO) formulation of our systematic review were based on the handbook (22) developed by the National Toxicology Program’s Office of Health Assessment and Translation (OHAT) for animal experimental studies (**Supplementary Table 0**).

### 2.2 Literature Search Strategy and Inclusion Criteria

Pubmed, Embase, Cochrane library, PROSPERO, and Google were searched using terms like “TCDD”, “meta”, or “systematic review” to find protocols or published systematic reviews similar with our topic to avoid duplication of work. Afterward, a systematic literature search process was performed in Pubmed, Embase, and Cochrane library on March 7, 2020. The search strategy was developed by combining the individual PECO parts as the formula: (1. species) AND (2. toxin) AND (3. outcomes). Controlled (i.e., medical subject headings) and free terms were applied to enrich the search result and avoid missing available articles, and the variation of word formation was simultaneously considered (**Supplementary Table 1**).

The inclusion and the exclusion criteria were developed to select eligible studies.

The inclusion criteria were as follows:

1) studies that have been peer reviewed,

2) studies in English,

3) studies on controlled animal TCDD exposure experiments,

4) studies with clear species limited to rat or mouse,

5) studies on male reproductive outcomes.

The exclusion criteria were as follows:

1) studies that were not original research articles,

2) studies without full text,

3) studies without male reproductive outcomes,

4) studies investigating neither rat nor mouse species,

5) studies with useless or unclear extractable data.

### 2.3 Literature Selection

Studies were screened by two researchers. The search outcomes were pooled together into the reference management software (EndNote X7, Thomson Scientific) to find potential duplications. Two researchers individually screened the search result in accordance with the inclusion and the exclusion criteria. During the first step, the title and abstract of each search result were browsed in accordance with the inclusion criteria to determine the potential eligible studies. Disagreements between the researchers about whether an article should be included were resolved by reviewing the full-text article.

### 2.4 Risk of Bias Assessment of Included Studies

The RoB of all included studies was assessed by evaluating the 11 questions for animal experimental study in accordance with the OHAT handbook (22) for animal studies. Briefly, a reviewer independently read each article and answered the 11 RoB questions on the basis of the contents of the article. The response options for each RoB question were Definitely Low (green, ++) if the included study showed direct evidence of low RoB practices, Probably Low (light green, +) if the included study showed indirect evidence of low RoB practices or may not cause significant bias, Probably High (light red, −) if the included study showed indirect evidence of high RoB practices or did not report relevant RoB questions, and Definitely High (red, −−) if the included study showed direct evidence of high RoB practices. Three key elements (i.e., randomization, experimental conditions, and blinding during study) were considered more important than other elements because these elements may seriously affect the confidence of outcomes. The evaluation was conducted twice. The first time was in reverse order of publication time, and the second time was in positive order of publication time to avoid inconsistencies in the evaluation results due to the order of reading. We displayed our assessment outcomes imitating the table developed by the OHAT book (22).

### 2.5 Data Extraction

Data elements were extracted for analyses in terms of number of animals (*n*), means (*m*), standard deviation (SD) or standard error (SE), life stage of outcome assessment, life stage of dosage administration, and general information (such as the title, publication time, and region of studies). SE data cannot be directly applied into the meta-analysis. Thus, the outcomes displayed as SE were transformed into SD in accordance with the formula: $SD=SE\times \sqrt{n}$ where *n* is the sample size for the following quantitative analysis. The data in tables and text were extracted directly. Data from figures were indirectly extracted using a graph digitizer software (GetData Graph Digitizer, Version 2.25, software available at http://getdata-graph-digitizer.com/download.php).

### 2.6 Data Standardization

Eighteen outcomes, including more than five articles, were subjected to meta-analysis. The design of the animal studies was different in the selection of animal strains, administration methods, and exposure time before the quantitative evaluation. Thus, the data were artificially standardized to prevent the data from being too discrete. The units of all outcomes were standardized (e.g., 100 ng/kg was standardized into 0.1 µg/kg). Thus, WMD rather than SMD was applied.

1. All strains of rats or mice were set as standard species “rat” or “mouse” individually.

a) Rat represents Sprague Dawley, LE, Albino, or Wistar rat or any strain of rat species,

b) Mouse represents CD-1 or C57BL/6 mouse or any strain of mouse species.

2. Exposure windows were standardized into five periods:

a) Pregestational: period before maternal gestational (< G0),

b) Gestational: period from the first day pregnancy was detected by performing vaginal smear or observing vaginal plug [G0, P0),

c) Lactational: period from the first day of birth to weaning [P0, P21),

d) Pubertal: period from weaning to postnatal day 56 [P21, P56),

e) Mature: period after postnatal day 56 (> P56).

3. Administration was classified into three methods.

a) i.p. represents intraperitoneal injection,

b) i.h. represents hypodermic injection,

c) p.o. represents peroral gavage.

4. Outcomes were standardized and classified into four groups:

a) sperm count (×10⁶), daily sperm production (×10⁶), sperm motility (%), and abnormal sperm (%),

b) serum testosterone (ng/ml), AGD (mm), and relative AGD (% body length),

c) ventral prostate weight (g), prostate weight (g), relative ventral prostate weight (% body weight), seminal vesicle weight (g), and relative seminal vesicle weight (% body weight),

d) testis weight (g), testes weight (g), relative testis weight (% body weight), relative testes weight (% body weight), epididymis weight (g), and relative epididymis weight (% body weight).

5. Dosage units were standardized as µg/kg, and dosage levels were divided into four groups

a) Low Level: (< 0.1 µg/kg),

b) Relative Low Level: [0.1 µg, 1 µg),

c) Relative High Level: [1 µg, 10 µg),

d) High Level: (>10 µg).

### 2.7 Meta-Analysis

The meta‐analysis was performed for outcomes if more than five studies were included. Effect sizes were generated by calculating the standardized (SMD) or the weighted (WMD) mean difference and 95% confidence interval (CI) of each intervention–control comparison. Individual SMDs or WMDs were pooled to reach a conclusion. The heterogeneity between each study was assessed using the *I²* statistics, and any degree of heterogeneity was acceptable due to the anticipated high heterogeneity of animal studies. The outcomes of heterogeneity assessment were defined into three groups, including low (*I²* <50%), moderate (50%≤*I²* <75%), and high (*I²* ≥75%) level. The fixed-effects model was applied if the heterogeneity level between studies was low or moderate, and the random-effects model was used if the heterogeneity level between studies was high. Stratified analysis for subtitles, such as species, exposure window, and dosage, was performed if more than three independent studies were included. Calculated p‐values < 0.05 indicated that the outcomes were statistically significant. If the effect between subgroups significantly differed with each other in the subgroup analysis, the subgroup was explained partly as the reason of heterogeneity. The Egger’s test was used to detect publication bias. If significant publication bias was detected, comparisons with small weights were excluded to observe the change in the effect size after exclusion, and the degree of effect of publication bias on the conclusion was judged on the basis of the amount of excluded data and the change in effect size. Statistical analysis was conducted using the statistical software STATA (StataSE12.0, Texas, USA, software available at https://www.stata.com/).

### 2.8 Confidence of Evidence Assessment

Confidence assessment reflects the credibility of the association between exposure to specific substances and corresponding human health outcomes. For each given outcome, the confidence rating was performed by considering the strengths and weaknesses in a series of human or animal studies that contribute to the body of evidence. The OHAT method for confidence of evidence assessment was applied in our analysis to judge and rate the confidence in the body of evidence on each outcome and conclude the level of health effect of each evidence. “High Confidence” indicates that future studies are not likely to change current conclusions, and “Very Low Confidence” indicates that future studies are very likely to change current conclusions (22). Briefly, for each outcome, initial confidence was set in accordance with four features (namely, controlled exposure, exposure prior to outcome, individual outcome data, and comparison group used), and the factors that may increase (i.e., magnitude, dose response, residual confounding, and consistency across species) and decrease (i.e., RoB, unexplained inconsistency, indirectness, imprecision, and publication bias) the level of confidence were considered. Disagreements were discussed by all reviewers. Initial confidence was upgraded and downgraded in accordance with the factors mentioned above to generate the final level of confidence (i.e., high, moderate, low, or very low) (22) and then transformed into human health effect levels (i.e., high, moderate, low, or inadequate).

## 3 Results

### 3.1 Search Results and Study Characteristics

**Figure 1:**
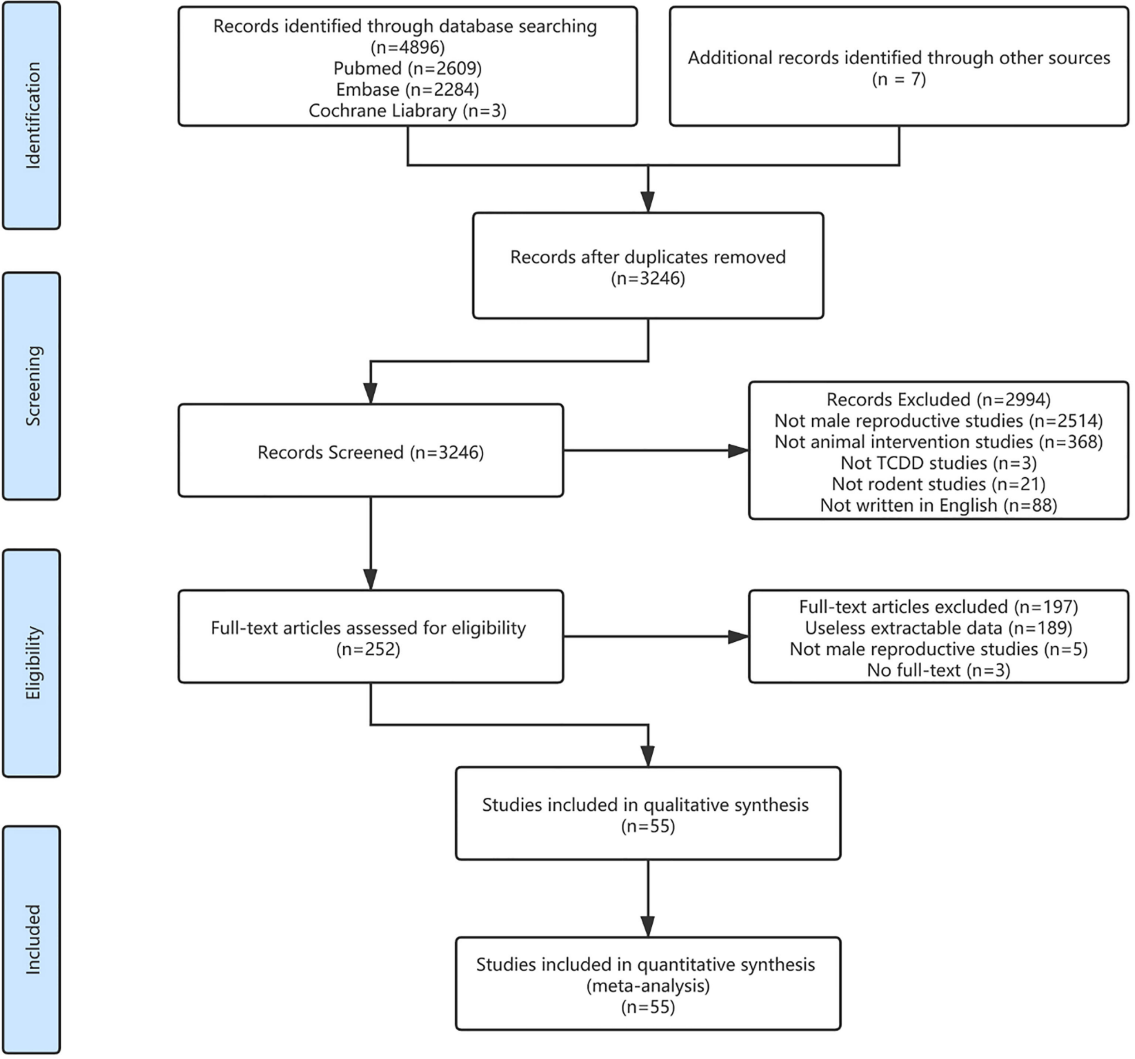
Study selection.

The systematic literature search was performed using the PECO statement and generated 3246 studies after finding duplication. After screening, a total of 55 studies (12–15, 19–21, 23–70) passed the inclusion criteria for data extraction. The literature selection process is displayed as the Preferred Reporting Items for Systematic Reviews and Meta-Analyses flow chart (**Figure 1** **)**. Among the 55 studies, 3 (5.45%), 1 (1.82%), 1 (1.82%), 2 (3.63%), 3 (5.45%), 5 (9.09%), 9 (16.36%), 8 (14.54), 4 (7.27%), 1 (1.82%), and 18 (32.73%) were from Brazil, Canada, Egypt, Finland, Germany, India, Japan, Korea, Turkey, UK, and USA, respectively. A total of 826 comparisons of 21 808 animals with species were included into the quantitative analysis. All studies were published from 1987 to 2019, and the dosage level ranged from 0.001 µg/kg to 1400 µg/kg. The average dosage level for the included studies were 14.22 µg/kg, and 26.04% of the studies applied 1 µg/kg. The oral route (p.o.), which accounted for 82.81%, was the most commonly used method of administration and similar to the route for human exposure to TCDD in the living environment. Half (58.72%) of the studies set the time window of exposure to the pregnancy stage, and 82.47% of these gestational exposure studies evaluated the male reproductive system parameters of pubertal stage and adulthood after birth, suggesting that researchers were concerned about the possible male reproductive system damage to the offspring caused by maternal exposure (**Table 1**).

**Table 1:** General characteristics of the included studies.

Study	Region	Species	Doses (ug/kg)	Exposure Stage	Assessment Stage	Administration	Indicators
Pohjanvirta, R., et al.(1987) (23)	USA	Han/Wistar rat	0, 125, 250, 375, 500, 625, 750, 1000, 1400	Mature	Mature	i.p.	a, g
Al-Bayati, Z. A. F., et al. (1988) (24)	USA	SD rat	0, 40	Pubertal	Pubertal	p.o.	a, c
Chahoud, I., et al. (1989) (25)	Germany	Wistar rat	0, 0.838	Pubertal- Mature	Mature	i.h.	a, c
Kleeman, J. M., et al. (1990) (26)	USA	SD rat	0, 100	Mature	Mature	p.o.	g, k
Chahoud, I., et al. (1992) (27)	Germany	Wistar rat	0, 0.5, 1, 3, 5	Mature	Mature	i.h.	a
Johnson, L., et al. (1992) (28)	USA	SD rat	0, 12.5, 25 50	Mature	Mature	i.p.	a, e, k, n, o
Mably, T. A., et al. (1992) (29)	USA	SD rat	0, 0.064, 0.16, 0.4, 1	Gestational	Mature	p.o.	b, e, n, o, q, r
Mably, T. A., et al. (1992) (30)	USA	SD rat	0, 0.064, 0.16, 0.4, 1	Gestational	Pubertal, Mature	p.o.	g, k, l, m, p
Bjerke, D. L. and R. E. Peterson (1994) (21)	USA	SD rat	0, 1	Gestational	Lactational, Mature	p.o.	b, e, g, l, m, n, p
Bjerke, D. L., et al. (1994) (19)	USA	SD rat	0, 0.7	Gestational	Lactational, Mature	p.o.	b, l, m
Gray, L. E., Jr., et al. (1995) (31)	USA	LE Hooded rat	0, 1	Gestational	Lactational, Mature	p.o.	b, e, l, n
Roman, B. L., et al. (1995) (32)	USA	SD rat	0, 1	Gestational	Lactational	p.o.	l, m, p
Sommer, R. J., et al. (1996) (33)	USA	SD rat	0, 1	Gestational	Mature	p.o.	n, o
Wilker, C., et al. (1996) (34)	USA	SD rat	0, 0.5, 1, 2	Gestational	Lactational, Mature	p.o.	e, h, k, l
Gray, L. E., et al. (1997) (20)	USA	LE Hooded rat	0, 0.05, 0.2, 0.8	Gestational	Pubertal, Mature	p.o.	b, e, g, n, o, p
Theobald, H. M. and R. E. Peterson (1997) (35)	USA	CD-1 mouse	0, 15, 30, 60	Gestational	Pubertal, Mature	p.o.	a, e, g, n, o
Cooke, G. M., et al. (1998) (36)	Canada	SD rat	0, 0.2, 1	Gestational	Lactational, Pubertal, Mature	p.o.	a, h
el-Sabeawy, F., et al. (1998) (37)	USA	SD rat	0, 5	Pubertal	Pubertal, Mature	i.p.	a, o
Faqi, A. S., et al. (1998) (38)	Germany	Wistar rat	0, 0.005, 0.012, 0.06	Pregestational-Pubertal	Mature	i.h.	c, f, j, n, o, p, s
Roman, B. L., et al. (1998) (39)	USA	SD rat	0, 1	Gestational	Mature	p.o.	n, o, k
Hamm, J. T., et al. (2000) (40)	USA	SD rat	0, 1	Gestational	Lactational, Pubertal, Mature	p.o.	k
Kang, K. S., et al. (2000) (41)	Korea	SD rat	0, 10	Pubertal	Pubertal	i.p.	a, g, n
Lin, T. M., et al. (2001) (42)	USA	C57BL/6 mouse	0, 5	Gestational	Mature	p,o,	a, c, e, f, n, o
Latchoumycandane, C., et al. (2002) (43)	India	Wistar rat	0, 0.001, 0.01, 0.1	Pubertal- Mature	Mature	p.o.	a, c, e, f, g, i, k, s
Latchoumycandane, C. and P. P. Mathur (2002) (44)	India	Wistar rat	0, 0.001, 0.01, 0.1	Pubertal- Mature	Mature	p.o.	a, e, g, k, o
Ohsako, S., et al. (2002) (45)	Japan	SD rat	0, 1	Gestational, Lactational	Mature	p.o, i.h.	b, d, e, f, g, i, m, n, o,
Kwon, Y. I., et al. (2004) (46)	Korea	C57BL/6 mouse	0, 27.5	Pubertal	Mature	i.h.	a, p
El-Tawil, O. S. and E. M. Elsaieed (2005) (47)	Egypt	SD rat	0, 0.05, 0.1, 0.2	Mature	Mature	p.o.	c, f, j, n, q, r, s
Ikeda, M., et al. (2005) (48)	Japan	SD rat	0, 0.2, 0.8	Gestational	Lactational	p.o.	l, p
Ikeda, M., et al. (2005) (49)	Japan	SD rat	0, 0.08	Pregestational-Lactational	Lactational	p.o.	d, l
Myllymäki, S. A., et al. (2005) (50)	Finland	SD rat	0, 0.04, 0.2, 1	Gestational	Lactational	p.o.	a, c, p
Yamano, Y., et al. (2005) (51)	Japan	SD rat	0, 0.3, 1	Lactational	Mature	i.h.	a, n
Yonemoto, J., et al. (2005) (52)	Japan	LE Hooded rat	0, 0.0125, 0.05, 0.2, 0.8	Gestational	Pubertal, Mature	p.o.	b, d, e, f, g, i, k, s
Haavisto, T. E., et al. (2006) (53)	Finland	SD rat	0, 0.04, 0.2, 1	Gestational	Lactational	p.o.	a, p
Park, J. S., et al. (2006) (54)	Korea	SD rat	0, 50	Mature	Mature	i.p.	b, e
Bell, D. R., et al. (2007) (55)	UK	Han rat	0, 0.05, 0.2, 1	Gestational	Mature	p.o.	b, d, e, f, h, j, k, n, q, r, s
Ohyama, K., et al. (2007) (56)	Japan	Wistar rat	0, 0.01	Gestational-Lactational	Lactational, Pubertal	i.h.	a
Choi, J. S., et al. (2008) (57)	Korea	SD rat	0, 50	Pubertal	Mature	i.p.	b, d, p
Jin, M. H., et al. (2008) (58)	Korea	C57BL/6	0, 1	Gestational	Pubertal, Mature	i.p.	l, m
Park, J. S., et al. (2008) (59)	Korea	SD rat	0, 40	Pubertal	Mature	i.p.	d, e, n
Dhanabalan, S. and P. P. Mathur (2009) (60)	India	Albino rat	0, 0.001	Mature	Mature	p.o.	a, p
Lee, S. C., et al. (2009) (61)	Korea	SD rat	0, 40	Pubertal	Mature	i.p.	b
Takeda, T., et al. (2009) (62)	Japan	Wistar rat	0, 1	Gestational	Mature	p.o.	d, f, i, m, s
Dhanabalan, S., et al. (2010) (63)	India	Wistar rat	0, 0.1	Mature	Mature	p.o.	e, g, k,n, p, q
Jin, M. H., et al. (2010) (64)	Korea	C57BL/6 mouse	0, 1	Lactational	Pubertal, Mature	p.o.	a, c, e, f, l, m, n
Dhanabalan, S., et al. (2011) (65)	India	Wistar rat	0, 0.1	Mature	Mature	p.o.	a, c, e, f, g, i, k, n, o, p, q, s
Sonmez, M., et al. (2011) (66)	Turkey	SD rat	0, 0.1	Mature	Mature	p.o.	p, q, r
Beytur, A., et al. (2012) (12)	Turkey	SD	0, 2	Mature	Mature	p.o.	a, e, h, k, n, p, q, r
Ciftci, O., et al. (2012) (13)	Turkey	SD	0, 2	Mature	Mature	p.o.	a, e, h, k, p, q, r
Fujimoto, N., et al. (2013) (67)	Japan	C57BL/6	0, 0.01, 0.1, 1	Lactational	Pubertal	i.p.	i
Oguz, F., et al. (2013) (68)	Turkey	SD rat	0, 2	Mature	Mature	p.o.	a, e, h, k, q, r
Sanabria, M., et al. (2016) (69)	Brazil	Wistar rat	0, 0.1, 0.5, 1	Gestational	Mature	p.o.	a, e, g, k, n, o, p
Erthal, R. P., et al. (2018) (14)	Brazil	SD rat	0, 1	Gestational	Mature	p.o.	a, o, p
Hattori, Y., et al. (2018) (70)	Japan	Wistar rat	0, 1	Gestational	Pubertal	p.o.	m, p
Silveira, L. T. R., et al. (2019) (15)	Brazil	Wistar rat	0, 1	Gestational	Lactational, Mature	p.o.	g, i, l, p

a: testis weight (g), b: testes weight (g), c: relative testis weight (% body weight), d: relative testes weight (% body weight), e: epididymis weight (g), f: relative epididymis weight (% body weight), g: ventral prostate weight (g), h: prostate weight (g), i: relative ventral prostate weight (% body weight), j: relative prostate weight (% body weight), k: seminal vesicle weight (g), l: anogenital distance (mm), m: relative anogenital distance (% body length), n: sperm count (×10⁶), o: daily sperm production (×10⁶), p: serum testosterone (ng/ml), q: sperm motility (%), r: abnormal sperm (%), s: relative seminal vesicle weight (% body weight). i.p. represents intraperitoneal injection, i.h. represents hypodermic injection, p.o. represents peroral gavage.

### 3.2 Assessment of RoB

In accordance with the recommendations of the OHAT handbook (22), the outcomes of RoB were assessed (**Table 2**). Most literature answered “probably high risk” to at least one of three key questions. “Allocation concealment” was not mentioned in all the included literature and was marked as “unreported” and translated into “probably high risk”. Regarding the randomness of experimental design, some studies mentioned “randomization” but did not give a detailed explanation of how they performed randomization (e.g., random number table and systematic sampling) and rated as “probably low risk”. In terms of the “experimental conditions”, most of the documents described the details of the animal’s circadian rhythm, food and drinking water, and room temperature. However, detailed questions, such as the height of the animal cages from the ground that had not been mentioned, remained and the evaluation of “definitely low risk” should be conservative. Thus, these studies were rated as “probably low risk”. According to the OHAT handbook, the quality of the literature was stratified. Two articles (64, 67) were classified as tier3 because the answers to the three key questions were all “probably high risk”, and the rest of the documents were classified as tier2. The outcomes of stratification were used to evaluate the confidence level in the later stage. The results of RoB are shown in **Table 2**.

**Table 2:** Risk of Bias assessment.

	Pohjanvirta, R., et al. (1987)	Al-Bayati, Z. A. F., et al. (1988)	Chahoud, I., et al. (1989)	Kleeman, J. M., et al. (1990)	Chahoud, I., et al. (1992)	Johnson, L., et al. (1992)	Mably, T. A., et al. (1992)	Mably, T. A., et al. (1992)	Bjerke, D. L. and R. E. Peterson (1994)	Bjerke, D. L., et al. (1994)	Gray, L. E., Jr., et al. (1995)	Roman, B. L., et al. (1995)	Sommer, R. J., et al. (1996)	Wilker, C., et al. (1996)	Gray, L. E., et al. (1997)	Theobald, H. M. and R. E. Peterson (1997)	Cooke, G. M., et al. (1998)	el-Sabeawy, F., et al. (1998)	Faqi, A. S., et al. (1998)	Roman, B. L., et al. (1998)	Hamm, J. T., et al. (2000)	Kang, K. S., et al. (2000)	Lin, T. M., et al. (2001)	Latchoumycandane, C., et al. (2002)	Latchoumycandane, C. and P. P. Mathur (2002)	Ohsako, S., et al. (2002)	Kwon, Y. I., et al. (2004)	
**Randomization**	**+**	**-**	**-**	**+**	**-**	**-**	**-**	**-**	**-**	**-**	**+**	**-**	**-**	**-**	**-**	**-**	**+**	**-**	**+**	**-**	**-**	**+**	**-**	**-**	**-**	**-**	**-**	
**Allocation concealment**	**-**	**-**	**-**	**-**	**-**	**-**	**-**	**-**	**-**	**-**	**-**	**-**	**-**	**-**	**-**	**-**	**-**	**-**	**-**	**-**	**-**	**-**	**-**	**-**	**-**	**-**	**-**	
**Confounding (design/analysis)**	**-**	**+**	**+**	**+**	**+**	**-**	**+**	**-**	**-**	**-**	**+**	**-**	**-**	**+**	**+**	**-**	**-**	**-**	**-**	**+**	**+**	**-**	**+**	**-**	**-**	**-**	**-**	
**Experimental conditions**	**+**	**+**	**+**	**+**	**+**	**+**	**+**	**+**	**+**	**+**	**+**	**+**	**+**	**+**	**+**	**+**	**-**	**+**	**+**	**+**	**+**	**+**	**+**	**+**	**+**	**+**	**+**	
**Blinding (during study)**	**-**	**-**	**-**	**-**	**-**	**-**	**-**	**-**	**-**	**-**	**-**	**-**	**-**	**-**	**-**	**-**	**-**	**-**	**-**	**-**	**-**	**-**	**-**	**-**	**-**	**-**	**-**	
**Complete outcome data**	**+**	**-**	**+**	**+**	**-**	**-**	**-**	**-**	**+**	**+**	**--**	**+**	**+**	**+**	**+**	**+**	**+**	**+**	**+**	**+**	**+**	**+**	**+**	**+**	**+**	**+**	**+**	
**Exposure characterization**	**++**	**++**	**++**	**++**	**-**	**++**	**++**	**++**	**++**	**-**	**-**	**--**	**-**	**-**	**-**	**++**	**++**	**++**	**++**	**-**	**-**	**-**	**-**	**-**	**-**	**++**	**--**	
**Outcome assessment**	**-**	**-**	**+**	**-**	**+**	**-**	**+**	**-**	**-**	**+**	**+**	**-**	**-**	**-**	**-**	**-**	**-**	**-**	**-**	**-**	**+**	**-**	**-**	**-**	**-**	**-**	**-**	
**Outcome reporting**	**+**	**+**	**+**	**+**	**+**	**+**	**+**	**+**	**+**	**+**	**-**	**+**	**+**	**+**	**+**	**+**	**+**	**+**	**+**	**+**	**+**	**+**	**-**	**+**	**+**	**+**	**+**	
**No other threat**	**-**	**-**	**-**	**-**	**-**	**-**	**-**	**-**	**-**	**-**	**-**	**-**	**-**	**-**	**-**	**-**	**-**	**-**	**-**	**-**	**-**	**-**	**-**	**-**	**-**	**-**	**-**	
**No other threat**	**-**	**-**	**-**	**-**	**-**	**-**	**-**	**-**	**-**	**-**	**-**	**-**	**-**	**-**	**-**	**-**	**-**	**-**	**-**	**-**	**-**	**-**	**-**	**-**	**-**	**-**	**-**	**-**
**Outcome reporting**	**+**	**+**	**-**	**+**	**+**	**+**	**+**	**+**	**+**	**+**	**+**	**+**	**+**	**+**	**-**	**+**	**+**	**+**	**+**	**+**	**+**	**+**	**+**	**+**	**+**	**+**	**+**	**+**
**Outcome assessment**	**+**	**+**	**-**	**-**	**-**	**-**	**+**	**-**	**+**	**+**	**-**	**+**	**-**	**+**	**+**	**+**	**+**	**+**	**-**	**+**	**+**	**-**	**+**	**+**	**+**	**+**	**+**	**+**
**Exposure characterization**	**-**	**+**	**+**	**++**	**-**	**++**	**++**	**-**	**++**	**-**	**+**	**+**	**-**	**+**	**+**	**-**	**++**	**-**	**++**	**-**	**++**	**++**	**-**	**++**	**-**	**-**	**-**	**-**
**Complete outcome data**	**-**	**-**	**+**	**+**	**+**	**+**	**+**	**-**	**+**	**+**	**+**	**+**	**+**	**+**	**+**	**+**	**+**	**+**	**+**	**+**	**+**	**+**	**+**	**+**	**+**	**+**	**+**	**+**
**Blinding (during study)**	**-**	**-**	**-**	**-**	**-**	**-**	**-**	**-**	**-**	**-**	**-**	**-**	**-**	**-**	**-**	**-**	**-**	**-**	**-**	**-**	**-**	**-**	**-**	**-**	**-**	**-**	**-**	**-**
**Experimental conditions**	**+**	**+**	**+**	**+**	**+**	**+**	**+**	**+**	**+**	**+**	**+**	**+**	**+**	**+**	**+**	**+**	**+**	**-**	**+**	**+**	**+**	**+**	**-**	**+**	**+**	**+**	**+**	**+**
**Confounding (design/analysis)**	**-**	**-**	**-**	**+**	**+**	**-**	**+**	**-**	**+**	**-**	**-**	**-**	**+**	**-**	**-**	**-**	**-**	**-**	**-**	**-**	**-**	**-**	**-**	**-**	**-**	**-**	**-**	**+**
**Allocation concealment**	**-**	**-**	**-**	**-**	**-**	**-**	**-**	**-**	**-**	**-**	**-**	**-**	**-**	**-**	**-**	**-**	**-**	**-**	**-**	**-**	**-**	**-**	**-**	**-**	**-**	**-**	**-**	**-**
**Randomization**	**+**	**-**	**-**	**+**	**-**	**+**	**+**	**-**	**+**	**+**	**-**	**+**	**+**	**-**	**-**	**-**	**-**	**-**	**+**	**+**	**+**	**+**	**-**	**-**	**-**	**-**	**-**	**+**
	**El-Tawil, O. S. and E. M. Elsaieed (2005)**	**Ikeda, M., et al. (2005)**	**Ikeda, M., et al. (2005)**	**Myllymäki, S. A., et al. (2005)**	**Yamano, Y., et al. (2005)**	**Yonemoto, J., et al. (2005)**	**Haavisto, T. E., et al. (2006)**	**Park, J. S., et al. (2006)**	**Bell, D. R., et al. (2007)**	**Ohyama, K., et al. (2007)**	**Choi, J. S., et al. (2008)**	**Jin, M. H., et al. (2008)**	**Park, J. S., et al. (2008)**	**Dhanabalan, S. and P. P. Mathur (2009)**	**Lee, S. C., et al. (2009)**	**Takeda, T., et al. (2009)**	**Dhanabalan, S., et al. (2010)**	**Jin, M. H., et al. (2010)**	**Dhanabalan, S., et al. (2011)**	**Sonmez, M., et al. (2011)**	**Beytur, A., et al. (2012)**	**Ciftci, O., et al. (2012)**	**Fujimoto, N., et al. (2013)**	**Oguz, F., et al. (2013)**	**Sanabria, M., et al. (2016)**	**Erthal, R. P., et al. (2018)**	**Hattori, Y., et al. (2018)**	**Silveira, L. T. R., et al. (2019)**

Definitely Low Risk of Bias (green, ++), Probably Low Risk of Bias (light green, +), Probably High Risk of Bias (light red, −), Definitely High Risk of Bias (red, −−).

### 3.3 Meta-Analysis

#### 3.3.1 Sperm Parameters

**Figure 2:**
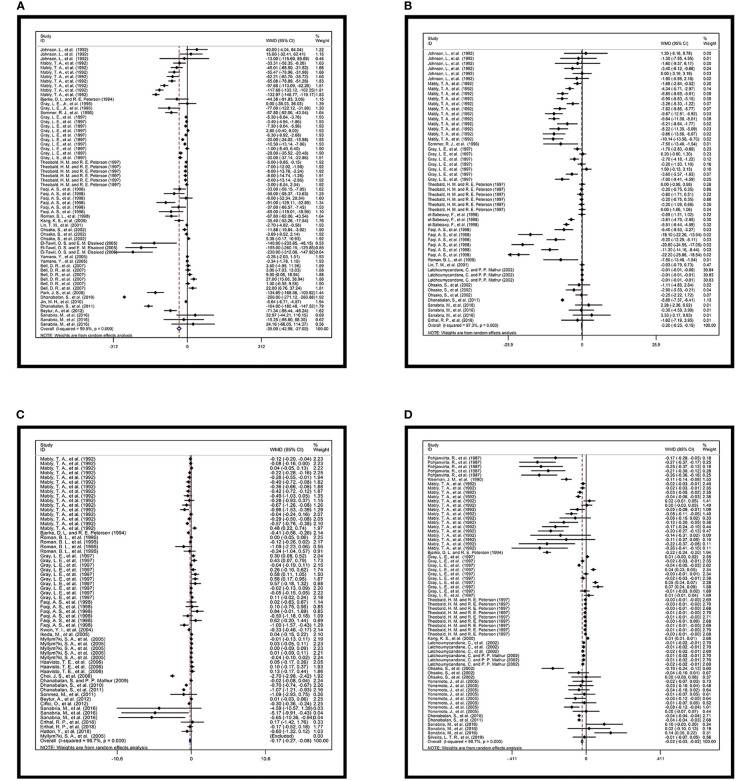
Forest plots of overall effects. Overall effects of TCDD and **(A)** sperm count (×10⁶), **(B)** daily sperm production (×10⁶), and **(C)** serum testosterone (ng/ml); **(D)** Total effect of TCDD and ventral prostate weight (g).

Sperm count was available from 62 comparisons provided by 21 studies (**Figure 2A** **).** The heterogeneity of the data was high (*I²* = 99.5%, *p* = 0.000). The sperm counts of rats and mice were examined. The exposure time spanned the life stage from prepregnancy to maturity, and the four levels of dosage were evaluated. The pooled WMD was −35 with 95% CI of −42.980 to −27.019 **(** **Table 3A** **).** Daily sperm production was also inversely affected in accordance with the data from 12 studies. The pooled WMD from 54 comparisons was −0.202 with 95% CI of −0.254 to −0.150 and *I²* of 97.3% (*p* = 0.000). The relative forest plots and data are shown in **Figure 2B** and **Table 3B**, respectively.

**Table 3:** Data of overall effects.

D+L pooled WMD	[95% Conf. Interval]	% Weight
A		
-35.000	(-42.980, -27.019)	100
Heterogeneity chi-squared = 11624.26 (d.f. = 61) p = 0.000		
I-squared (variation in WMD attributable to heterogeneity) = 99.5%		
B		
D+L pooled WMD	[95% Conf. Interval]	% Weight
-0.202	(-0.254, -0.150)	100
Heterogeneity chi-squared = 1992.72 (d.f. = 53) p = 0.000		
I-squared (variation in WMD attributable to heterogeneity) = 97.3%		
C		
D+L pooled WMD	[95% Conf. Interval]	% Weight
-0.171	(-0.269, -0.073)	100
Heterogeneity chi-squared = 1790.47 (d.f. = 61) p = 0.000		
I-squared (variation in WMD attributable to heterogeneity) = 96.6%		
D		
CD+L pooled WMD	[95% Conf. Interval]	% Weight
-0.022	(-0.027, -0.017)	100
Heterogeneity chi-squared = 1990.02 (d.f. = 65) p = 0.000		
I-squared (variation in WMD attributable to heterogeneity) = 96.7%		

Overall effects of TCDD and A: sperm count (×10⁶), B: daily sperm production (×10⁶), and C: serum testosterone (ng/ml); D: Total effect of TCDD and ventral prostate weight (g).

In addition to the negative effect on sperm quantity-associated parameter, low sperm quality was related to TCDD exposure. The percentage of sperm motility data from nine studies suggested that TCDD exposure significantly reduced the rats’ sperm motility, and the pooled WMD was −7.365. The interstudy heterogeneity of this indicator was slightly reduced compared with other indicators (*I²* = 89.3%). Similarly, the percentage of the abnormal sperm of rats increased after TCDD exposure, and its pooled WMD was 3.142 with 95% CI of 1.632 to 4.653. High heterogeneity was also detected among comparisons (*I²* = 94.3%). The relative forest plots and data are shown in **Supplementary Figures 1A, B** and **Supplementary Tables 1A, B**.

#### 3.3.2 Testosterone and AGD

Twenty studies reported plasma testosterone concentration indicators after TCDD exposure. A total of 62 comparisons were extracted and included in the quantitative analysis. Except one study, which was a mouse study, all other studies were rat studies. The time windows of exposure were from prepregnancy to adulthood (**Figure 2C** **)**. The overall effect was negative. The pooled WMD was −0.171 with 95% CI of −0.269 to −0.073. High heterogeneity was also detected (*I²* = 96.6%, *p* = 0.000). Data are shown in **Table 3C**.

AGD is an efficient parameter in the determination of intrauterine exposure to endocrine-disrupting chemicals and an effective tool in the investigation of *in utero* androgen production (71). AGD data were available in 12 studies, generating 41 comparisons focusing on pregnancy and lactation exposure, and the dosage level ranged from low to relatively high **(** **Supplementary Figure 1C** and **Supplementary Table 2C** **)**. The overall pooled WMD was −0.536 with 95% CI of −0.659 to −0.414, and high heterogeneity was detected (*I²* = 92.3%, *p* = 0.000). The relative AGD data were also analyzed. Details are shown in **Supplementary Figure 1D** and **Supplementary Table 2D**.

#### 3.3.3 Seminal Vesicle and Prostate Gland

A total of 66 pairs of comparisons focusing on ventral prostate weight were extracted from the included literature (i.e., rat and mouse studies). The effects of TCDD exposure from pregnancy to adulthood were studied, but very few studies were available on pubertal and lactational exposures, with only one comparison individually. The pooled WMD of the ventral prostate weight was −0.022 with 95% CI of −0.027 to −0.017 and high heterogeneity (*I²* = 96.7%, *p* = 0.000). Relative forest plots and data are shown in **Figure 2D** and **Table 3D**, respectively.

The data on the weight of the seminal vesicles were extracted from 15 studies including 57 comparisons, but the data from mouse studies were not available in any study. The exposure window spanned the gestational to mature life stage, and the effect of four dosage levels were examined. The pooled WMD was −0.041 with 95% CI of −0.051 to −0.032, and *I²* was 94.8% (*p* = 0.000). The relative forest plots and data are shown in **Supplementary Figure 1E** and **Supplementary Table 2E**, respectively.

In addition, other data associated with prostate and seminal vesicle were quantitatively analyzed. The forest plots and data are available in **Supplementary Figures 1F**, **2A** and **Supplementary Tables 2F**, **3A, B**.

#### 3.3.4 Testis and Epididymis

A total of 25 articles generating 97 comparisons provided testis weight data. Rat and mouse experiments were performed. The exposure period of the studies ranged from pregnancy to adulthood, and the dosage levels were from low to high. The pooled WMD was −0.035 with 95% CI of −0.046 to −0.025 and *I²* of 98.2% (*p* = 0.000), suggesting that TCDD exposure may reduce testicular weight but with high heterogeneity. The forest plots and data are shown in **Supplementary Figure 2C** and **Supplementary Table 2C**, respectively.

The epididymis weight data were extracted in 22 articles, and 94 pairs of comparisons from rats and mice were included in the analysis. The exposure phase also covered life stages from pregnancy to adulthood, but only one comparison of pubertal exposure data was extracted. The dosage levels of TCDD ranged from low to high. The pooled WMD of the total effect was −0.029 with the 95% CI of −0.034 to −0.023), and the heterogeneity between studies was strong (*I²* = 95.1%, *p* = 0.000), suggesting that TCDD exposure may cause the atrophy of epididymis. The forest plots and data are shown in **Supplementary Figure 2D** and **Supplementary Table 3D**, respectively.

In addition to the absolute weight data of the epididymis and testis, the testes weight and the relative weight of the above two organs were analyzed. The forest plots and their details are shown in **Supplementary Figures 3A–D** and **Supplementary Tables 4A–D**.

#### 3.3.5 Subgroup Analysis

Subgroup analysis was performed to discover the effects of exposure on related indicators under different subcategories. Each indicator was further analyzed in terms of species, exposure time window, and dosage level. Subgroup analysis revealed that some of the conclusions may have cross-species consistency (e.g., testis weight) and found differences in sensitivity of different tissues and organs to TCDD at different time windows. The relative outcomes of subgroup analysis and detailed data were classified by indicator (**Supplementary Figures 4**-**21** and **Supplementary Tables 5**-**22** **)**. After subgroup analysis, if the heterogeneity of the subgroup is lower than the original group and exceeds a level, it is considered that the source of the heterogeneity of the original group is related to the classification of the subgroup. If the heterogeneity of the subgroup is still high and does not differ from the original group by more than one level, it is considered that the source of the heterogeneity is related to the experimental design of the included study.

### 3.4 Evaluation of Publication Bias

The Egger’s test was applied to determine the publication bias. If the 95% CI did not include 0 through the Egger’s test, significant publication bias was detected (**Supplementary Figure 22**). The Egger’s test suggested that significant publication bias was detected in 10 outcomes. After gradually excluding comparisons with small weights, the indicators with publication bias were divided into two categories: 1. The publication bias can be neutralized by eliminating the comparisons with small weights, and the conclusion remains unchanged; and 2. The publication bias can be neutralized by eliminating the comparisons with small weights, but the conclusion changes or publication bias exists after 50% of comparisons with small weight are excluded. The publication bias in category 1 literature was considered to have minimal effect on conclusions. The publication bias was considered to have a great effect on conclusions in the two types of literature. The publication bias classification results were used to evaluate the confidence level at a later stage. The test outcomes and results of the adjusted details are shown in **Supplementary Tables 23**, **24**.

### 3.5 Confidence Rating

Given that all included studies were animal intervention studies, the initial level of evidence was rated as the highest level. Because the data of each outcome were all or mostly from the tier2 study category, a downgrade was performed in the RoB. The forest plots of the relative testis weight (% body weight) and daily sperm production (×10⁶) were discrete and had high *I²*. Thus, the rate of these two outcomes should be degraded to “Unexplained Inconsistency”. The publication biases of relative testis weight (% body weight), AGD (mm), seminal vesicle weight (g), and relative seminal vesicle weight (% body weight) were considered to influence the conclusions. Thus, these parameters were downgraded in the publication bias. The outcomes of testis weight (g), relative testis weight (% body weight), epididymis weight (g), relative epididymis weight (% body weight), ventral prostate weight, AGD (mm), relative AGD (% body length), sperm count (×10⁶), daily sperm production (×10⁶), and serum testosterone (ng/ml) appeared to have consistent conclusions among the species subgroup analysis and were upgraded in the “cross-species” stage. The conclusions of the systematic review were translated into the final health effect on the basis of their rating outcome according to the OHAT manual. Finally, 13 outcomes were considered transformable to human health and had moderate or high confidence. The results of the confidence rating and health effect evaluation are shown in **Table 4**.

**Table 4:** Outcomes of confidence rating and health effect assessment.

		Factors decreasing confidence					Factors increasing confidence					
	Body of Evidence	Risk of Bias	Unexplained Inconsistency	Indirectness	Imprecision	Publication Bias	Magnitude	Dose Response	Residual Confounding	Consistency Across Species	Final Rating	Level of evidence for health effect
**testis weight(g)**	**++++**	**↓**	**-**	**-**	**-**	**-**	**-**	**-**	**-**	**↑**	**++++**	**High**
**testes weight(g)**	**++++**	**↓**	**-**	**-**	**-**	**-**	**-**	**-**	**-**	**-**	**+++**	**Moderate**
**relative testis weight(% body weight)**	**++++**	**↓**	**↓**	**-**	**-**	**↓**	**-**	**-**	**-**	**↑**	**++**	**Inadequate**
**relative testes weight(% body weight)**	**++++**	**↓**	**-**	**-**	**-**	**-**	**-**	**-**	**-**	**-**	**+++**	**Inadequate**
**epididymis weight(g)**	**++++**	**↓**	**-**	**-**	**-**	**-**	**-**	**-**	**-**	**↑**	**++++**	**High**
**relative epididymis weight(% body weight)**	**++++**	**↓**	**-**	**-**	**-**	**-**	**-**	**-**	**-**	**↑**	**++++**	**High**
**ventral prostate weight(g)**	**++++**	**↓**	**-**	**-**	**-**	**-**	**-**	**-**	**-**	**↑**	**++++**	**High**
**prostate weight(g)**	**++++**	**↓**	**-**	**-**	**-**	**-**	**-**	**-**	**-**	**-**	**+++**	**Inadequate**
**relative ventral prostate weight(% body weight)**	**++++**	**↓**	**-**	**-**	**-**	**-**	**-**	**-**	**-**	**-**	**+++**	**Moderate**
**seminal vesicle weight(g)**	**++++**	**↓**	**-**	**-**	**-**	**↓**	**-**	**-**	**-**	**-**	**++**	**Low**
**anogenital distance(mm)**	**++++**	**↓**	**-**	**-**	**-**	**↓**	**-**	**-**	**-**	**↑**	**+++**	**Moderate**
**relative anogenital distance(% body length)**	**++++**	**↓**	**-**	**-**	**-**	**-**	**-**	**-**	**-**	**↑**	**++++**	**High**
**sperm count(×10⁶),**	**++++**	**↓**	**-**	**-**	**-**	**-**	**-**	**-**	**-**	**↑**	**++++**	**High**
**daily sperm production(×10⁶)**	**++++**	**↓**	**↓**	**-**	**-**	**-**	**-**	**-**	**-**	**↑**	**+++**	**Moderate**
**serum testosterone(ng/ml)**	**++++**	**↓**	**-**	**-**	**-**	**-**	**-**	**-**	**-**	**↑**	**++++**	**High**
**sperm motility(%)**	**++++**	**↓**	**-**	**-**	**-**	**-**	**-**	**-**	**-**	**-**	**+++**	**Moderate**
**abnormal sperm(%)**	**++++**	**↓**	**-**	**-**	**-**	**-**	**-**	**-**	**-**	**-**	**+++**	**Moderate**
**relative seminal vesicle weight(% body weight)**	**++++**	**↓**	**-**	**-**	**-**	**↓**	**-**	**-**	**-**	**-**	**++**	**Low**

## 4 Discussion

According to the results of our systematic review, TCDD exposure has a negative effect on the overall male reproductive system of rats and mice. Exposure to TCDD may cause atrophy of the testis (WMD: −0.035, 95% CI: −0.046 to −0.025) and epididymis (WMD: −0.029, 95% CI: −0.034 to −0.023), dysplasia of the ventral lobe prostate (WMD: −0.022, 95% CI: −0.027 to −0.017) and seminal vesicles (WMD: −0.041, 95% CI: −0.051 to −0.032), severely reduced sperm count (WMD: −35, 95% CI: −42.980 to −27.019), and other negative effects. In the subgroup analysis, most outcomes have the consistent conclusions across species, and the toxic effects vary due to different exposure time windows. For example, the testicular and the epididymal weights are sensitive to TCDD exposure during adolescence and adulthood, whereas sperm-related parameters are sensitive to TCDD exposure during pregnancy or adolescence. TCDD exposure causes a slight decrease in serum testosterone (WMD: −0.171, 95% CI: −0.269 to −0.073) and short AGD (WMD: −0.536, 95% CI: −0.269 to 0.073). The disruption of the androgenic system may partly explain the adverse effect of TCDD on the male reproductive system. Previous human studies have shown that early exposure to TCDD can cause negative changes in male reproductive outcomes. Compared with nonexposure, exposure to TCDD prior to puberty reports significantly reduced sperm concentration (53.6 × 10^6^/ml, p = 0.03), and exposure during pregnancy leads to a significant decrease in the sperm concentration (36.3 × 10^6^/mL, p = 0.002) of male offspring (6). High peripubertal blood TCDD concentration is associated with low sperm count, which is similar to the result of our analysis (72). In animal studies, no significant change in relative testis weight (WMD: −0.007, 95% CI: −0.035 to 0.021) is observed, which may be because TCDD causes developmental disorders in the male reproductive system and changes in bodyweight (14, 70).

Reports showed that a variety of compounds in the environment can induce adverse changes in the reproductive system (73). TCDD is considered a widespread environmental toxin that induces developmental and reproductive dysfunctions (74). TCDD exposure may result in numerous male reproductive toxic effects, such as spermatogenesis retardance and low testicular and sex organ weight (13). According to studies focusing on its toxicological mechanisms, TCDD is believed to exert its effects by binding to an intracellular transcription factor known as aryl hydrocarbon receptor (AhR) with high affinity (15). The treatment of wild-type rats with TCDD during pregnancy may result in gonadotropins and testicular steroid synthesis disorders in their offspring, but AhR knockout rats are not sensitive to this treatment (70). Compared with the control group, the wild-type mice treated with TCDD show a significant reduction in prostate protein markers and significantly shortened AGD, but these changes do not occur in AhR knockout mice (75). AhR activation can enhance the enzymes related to steroid metabolism, such as UDP-glucuronosyltransferase (UGT) 1A6/7, UGT1A8/9, and CYP1A2 activities, in pregnant rats and their offspring, thereby accelerating the metabolism of glucocorticoids and leading to decreased serum cortisol concentration. Decreased cortisol concentration causes growth hormone dysfunction and induces developmental disorders in the offspring (76). AhR can interfere with the synthesis of steroid hormones to indirectly damage the development of the male reproductive system. Moreover, AhR, as a nuclear receptor, can directly compete with the androgen receptor (AR), a nuclear receptor, to recruit cofactors or assemble the proteasome through the ubiquitination pathway for the direct degradation of the AR protein and the destruction of the normal physiological function of the AR pathway (77).

**Figure 3:**
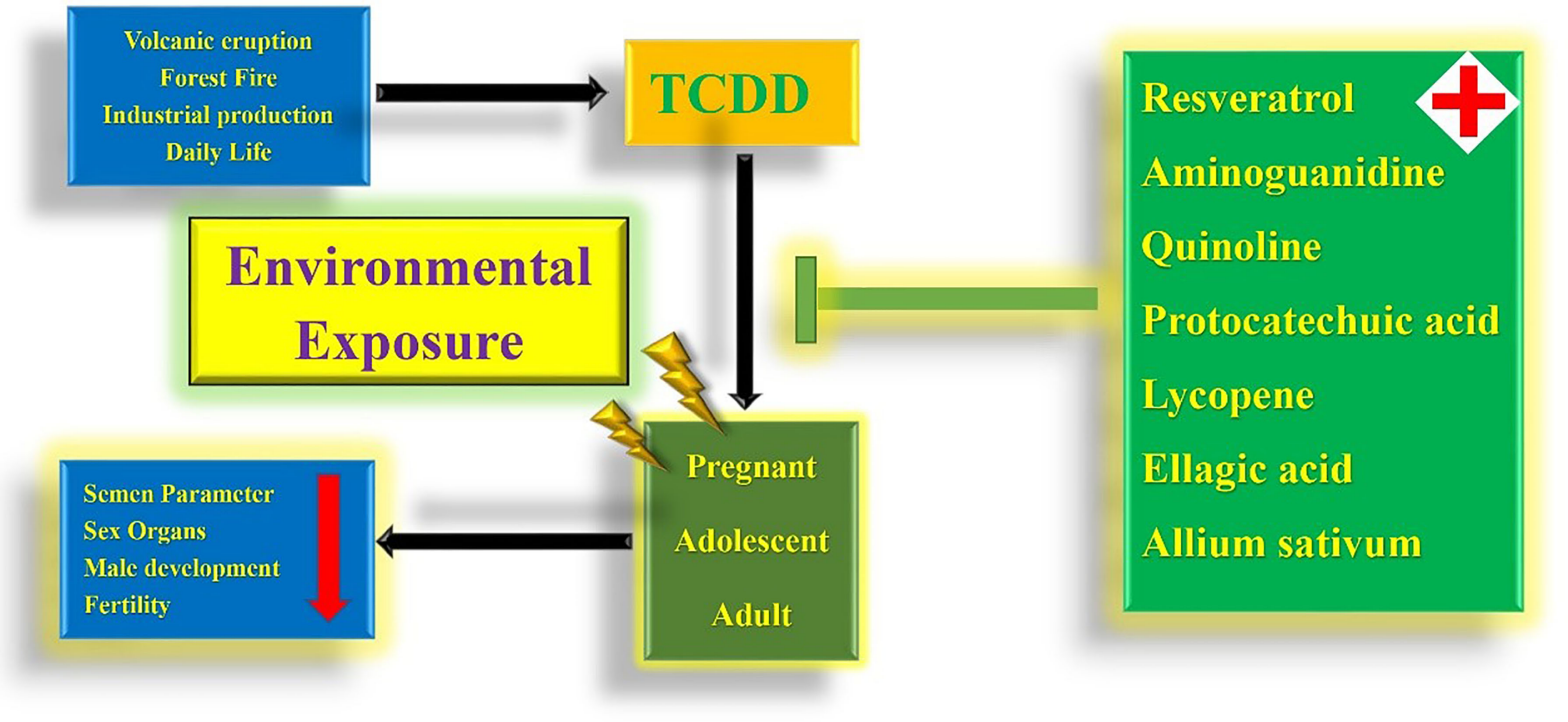
Holistic view of TCDD environmental exposure and drug remedy.

Considering that the male reproductive toxicity of TCDD is explained, researchers are also working to find possible protective agents. Recent studies found that resveratrol can rescue the toxic effects caused by TCDD to some extent. TCDD administration induces the reduction in the number of prostatic buds, which may cause prostate dysplasia, whereas this adverse effect is not significantly observed when TCDD is taken with resveratrol simultaneously (15). The reduction in Sertoli cell numbers and abnormal seminiferous tubule numbers caused by TCDD exposure can be protected by resveratrol (14). In addition to resveratrol, aminoguanidine can partially reverse the toxic effects of TCDD on male reproductive outcomes and reverse the toxic effect of TCDD on semen parameters (68). The reduction in serum testosterone concentration caused by TCDD can be rescued by the simultaneous administration of quinoline, and the histological morphology of the damaged seminiferous tubules can be remedied (13). In addition to the above agents, many plant extracts, such as protocatechuic acid, lycopene, ellagic acid, and ethanol extract of *Allium sativum*, have been shown to play a protective role in the male reproductive system damage caused by TCDD and may become potential clinical protective drugs in the future (12, 59, 61, 66) **(** **Figure 3** **)**.

The effect of environmental endocrine disruptors on development and reproductive systems has attracted worldwide attention. In the past few years, male reproductive system diseases have become serious. The exposure to chemicals, such as bisphenol, pesticides, phthalates, and dioxin, are associated with poor reproductive outcomes (78). Evidence of male reproductive system damage due to exposure to TCDD, a well-known endocrine disruptor, has been confirmed in many animal experiments. More human observational studies need to be carried out to deeply understand the reproductive toxicity of TCDD. In addition, future research should focus on the reproductive toxicity mechanism of TCDD and other possible molecular pathways or targets. Researchers should find more effective protective drugs and study their protective mechanisms to provide solutions to the possible male reproductive system damage caused by TCDD.

To our knowledge, this is the first systematic review to quantitatively assess TCDD exposure and male reproductive system damage at different life stages and dosages. This review indicates that TCDD can cause damage to the male reproductive system of rodents. Although our conclusions are supported by many studies, some shortcomings remain. For example, the amount of data included is not large enough, and the design of animal experiments is generally not rigorous, which causes the overall quality of the included literature to become low. Moreover, the publication bias of several outcomes may affect the credibility of our conclusions. More rigorously designed studies should be carried out to enrich the evidence of TCDD male reproductive toxicity. In conclusion, multiple results of animal experiments confirm the male reproductive toxicity of TCDD, which may cause the human health problems.

## 5 Conclusions

TCDD may cause male reproductive system damage during pregnancy, adolescence, and adulthood. Notably, exposure during pregnancy causes significant semen abnormalities in male offspring, which may lead to decreased fertility of the next generation. For the health of the next generation of male reproductive systems and the ethnic reproduction of human beings, exposure during pregnancy should be taken seriously. More in-depth research on environmental reproductive poisons is needed.

## Data Availability Statement

The original contributions presented in the study are included in the article/**Supplementary Material**. Further inquiries can be directed to the corresponding authors.

## Author Contributions

TZ: data curation, formal analysis, and writing–original draft. XiZ: data curation and formal analysis. XR: data curation and formal analysis. XuZ and JW: writing–review and editing. SW: funding acquisition, conceptualization, and resources. ZW: conceptualization and resources methodology. All authors contributed to the article and approved the submitted version.

## Funding

This work was supported by the National Nature Science Foundation of China (No. 81800587).

## Conflict of Interest

The authors declare that the research was conducted in the absence of any commercial or financial relationships that could be construed as a potential conflict of interest.

## Publisher’s Note

All claims expressed in this article are solely those of the authors and do not necessarily represent those of their affiliated organizations, or those of the publisher, the editors and the reviewers. Any product that may be evaluated in this article, or claim that may be made by its manufacturer, is not guaranteed or endorsed by the publisher.

## Supplementary Material

Supplementary Figure 1 Forest plots of overall effects. **(A)**: Overall effect of TCDD and sperm motility (%); **(B)**: Overall effect of TCDD and abnormal sperm (%); **(C)**: Overall effect of TCDD and anogenital distance(mm); **(D)**: Overall effect of TCDD and relative anogenital distance (%body length); **(E)**: Overall effect of TCDD and seminal vesicle weight (g); **(F)**: Overall effect of TCDD and prostate weight (g)

Supplementary Figure 2 Forest plots of overall effects.**(A)**: Overall effect of TCDD and relative ventral prostate weight (%body weight); **(B)**: Overall effect of TCDD and relative seminal vesicle weight (%body weight); **(C)**: Overall effect of TCDD and Testis Weight (g); **(D)**: Overall effect of TCDD and Epididymis weight(g)

Supplementary Figure 3 Forest plots of overall effects**. (A)**: Overall effect of TCDD and Testes Weight (g); **(B)**: Overall effect of TCDD and Relative testis weight (%body weight); **(C)**: Overall effect of TCDD and Relative testes weight (%body weight); **(D)**: Overall effect of TCDD and Relative epididymis weight (%body weight)

Supplementary Figure 4 Forest plots of Subgroup analysis.**(A)**: Effect of TCDD and testis weight (g) by Species Subgroups; **(B)**: Effect of TCDD and testis weight (g) by Species Subgroups; **(C)**: Effect of TCDD and testis weight (g) by Exposure Windows Subgroups

Supplementary Figure 5 Forest plots of Subgroup analysis. **(A)**: Effect of TCDD and testes weight (g) by Species Subgroups; **(B)**: Effect of TCDD and testes weight (g) by Exposure Windows Subgroups; **(C)**: Effect of TCDD and Testis Weight (g) by Dosage Levels Subgroups

Supplementary Figure 6 Forest plots of Subgroup analysis. **(A)**: Effect of TCDD and relative testis weight (%body weight) by Species Subgroups; **(B)**: Effect of TCDD and relative testis weight (%body weight) by Exposure Windows Subgroups; **(C)**: Effect of TCDD and relative testis weight (%body weight) by Dosage Levels Subgroups

Supplementary Figure 7 Forest plots of Subgroup analysis. **(A)**: Effect of TCDD and Relative testes weight (%body weight) by Species Subgroups; **(B)**: Effect of TCDD and Relative testes weight (%body weight) by Exposure Windows Subgroups; **(C)**: Effect of TCDD and Relative testes weight (%body weight) by Dosage Levels Subgroups

Supplementary Figure 8 Forest plots of Subgroup analysis. **(A)**: Effect of TCDD and epididymis weight (g) by Species Subgroups; **(B)**: Effect of TCDD and epididymis weight (g) by Exposure Windows Subgroups; **(C)**: Effect of TCDD and epididymis weight (g) by Dosage Levels Subgroups

Supplementary Figure 9 Forest plots of Subgroup analysis. **(A)**: Effect of TCDD and relative epididymis weight (%body weight) by Species Subgroups; (**B)**: Effect of TCDD and relative epididymis weight (%body weight) by Exposure Windows Subgroups; C: Effect of TCDD and relative epididymis weight (%body weight) by Dosage Levels Subgroups

Supplementary Figure 10 Forest plots of Subgroup analysis. **(A)**: Effect of TCDD and ventral prostate weight (g) by Species Subgroups; **(B)**: Effect of TCDD and ventral prostate weight (g) by Exposure Windows Subgroups; **(C)**: Effect of TCDD and ventral prostate weight (g) by Dosage Levels Subgroups

Supplementary Figure 11 Forest plots of Subgroup analysis. **(A)**: Effect of TCDD and prostate weight (g) by Species Subgroups; **(B)**: Effect of TCDD and prostate weight (g) by Exposure Windows Subgroups; **(C)**: Effect of TCDD and prostate weight (g) by Dosage Levels Subgroups

Supplementary Figure 12 Forest plots of Subgroup analysis. **(A)**: Effect of TCDD and relative ventral prostate weight (%body weight) by Species Subgroups; **(B)**: Effect of TCDD and relative ventral prostate weight (%body weight) by Exposure Windows Subgroups; **(C)**: Effect of TCDD and relative ventral prostate weight (%body weight) by Dosage Levels Subgroups

Supplementary Figure 13 Forest plots of Subgroup analysis. **(A)**: Effect of TCDD and seminal vesicle weight (g) by Species Subgroups; **(B)**: Effect of TCDD and seminal vesicle weight (g) by Exposure Windows Subgroups; **(C)**: Effect of TCDD and seminal vesicle weight (g) by Dosage Levels Subgroups

Supplementary Figure 14 Forest plots of Subgroup analysis. **(A)**: Effect of TCDD and anogenital distance (mm) by Species Subgroups; **(B)**: Effect of TCDD and anogenital distance (mm) by Exposure Windows Subgroups; **(C)**: Effect of TCDD and anogenital distance (mm) by Dosage Levels Subgroups

Supplementary Figure 15 Forest plots of Subgroup analysis. **(A)**: Effect of TCDD and relative anogenital distance (%body length) by Species Subgroups; **(B)**: Effect of TCDD and relative anogenital distance (%body length) by Exposure Windows Subgroups; **(C)**: Effect of TCDD and relative anogenital distance (%body length) by Dosage Levels Subgroups

Supplementary Figure 16 Forest plots of Subgroup analysis. **(A)**: Effect of TCDD and sperm count (×10⁶) by Species Subgroups; **(B)**: Effect of TCDD and sperm count (×10⁶) by Exposure Windows Subgroups; **(C)**: Effect of TCDD and sperm count (×10⁶) by Dosage Levels Subgroups

Supplementary Figure 17 Forest plots of Subgroup analysis. **(A)**: Effect of TCDD and daily sperm production (×10⁶) by Species Subgroups; **(B)**: Effect of TCDD and daily sperm production (×10⁶) by Exposure Windows Subgroups; **(C)**: Effect of TCDD and daily sperm production (×10⁶) by Dosage Levels Subgroups

Supplementary Figure 18 Forest plots of Subgroup analysis. **(A)**: Effect of TCDD and serum testosterone (ng/ml) by Species Subgroups; **(B)**: Effect of TCDD and serum testosterone (ng/ml) by Exposure Windows Subgroups; **(C)**: Effect of TCDD and serum testosterone (ng/ml) by Dosage Levels Subgroups

Supplementary Figure 19 Forest plots of Subgroup analysis. **(A)**: Effect of TCDD and sperm motility (%) by Species Subgroups; **(B)**: Effect of TCDD and sperm motility (%) by Exposure Windows Subgroups; **(C)**: Effect of TCDD and sperm motility (%) by Dosage Levels Subgroups

Supplementary Figure 20 Forest plots of Subgroup analysis. **(A)**: Effect of TCDD and abnormal sperm (%) by Species Subgroups; **(B)**: Effect of TCDD and abnormal sperm (%) by Exposure Windows Subgroups; **(C)**: Effect of TCDD and abnormal sperm (%) by Dosage Levels Subgroups

Supplementary Figure 21 Forest plots of Subgroup analysis. **(A)**: Effect of TCDD and relative seminal vesicle weight (%body weight) by Species Subgroups; **(B)**: Effect of TCDD and relative seminal vesicle weight (%body weight) by Exposure Windows Subgroups; **(C)**: Effect of TCDD and relative seminal vesicle weight (%body weight) by Dosage Levels Subgroups

Supplementary Figure 22 Egger’s test for Publication Bias. **(A)**: Egger’s test for Publication Bias of testis weight (g). Each point represents an individual comparison. **(B)**: Egger’s test for Publication Bias of testes weight (g). Each point represents an individual comparison. **(C)**: Egger’s test for Publication Bias of relative testis weight (%body weight). Each point represents an individual comparison. **(D)**: Egger’s test for Publication Bias of relative testes weight (%body weight). Each point represents an individual comparison. **(E)**: Egger’s test for Publication Bias of epididymis weight (g). Each point represents an individual comparison. **(F)**: Egger’s test for Publication Bias of relative epididymis weight (%body weight). Each point represents an individual comparison. **(G)**: Egger’s test for Publication Bias of ventral prostate weight (g). Each point represents an individual comparison. **(H)**: Egger’s test for Publication Bias of prostate weight (g). Each point represents an individual comparison. **(I)**: Egger’s test for Publication Bias of relative ventral prostate weight (%body weight). Each point represents an individual comparison. **(J)**: Egger’s test for Publication Bias of seminal vesicle weight (g). Each point represents an individual comparison. **(K)**: Egger’s test for Publication Bias of anogenital distance (mm). Each point represents an individual comparison. **(L)**: Egger’s test for Publication Bias of relative anogenital distance (%body length). Each point represents an individual comparison. **(M)**: Egger’s test for Publication Bias of sperm count (×10⁶). Each point represents an individual comparison. **(N)**: Egger’s test for Publication Bias of daily sperm production (×10⁶). Each point represents an individual comparison. **(O)**: Egger’s test for Publication Bias of serum testosterone (ng/ml). Each point represents an individual comparison. **(P)**: Egger’s test for Publication Bias of sperm motility (%). Each point represents an individual comparison. **(Q)**: Egger’s test for Publication Bias of abnormal sperm (%). Each point represents an individual comparison. **(R)**: Egger’s test for Publication Bias of relative seminal vesicle weight (%body weight). Each point represents an individual comparison.

Supplementary Table 0 Topic statement and problem formulation.

Supplementary Table 1 Studies Search Strategy and Search Outcomes.

Supplementary Table 2 Data details of overall effects. **(A)**: Overall effect of TCDD and sperm motility (%); **(B)**: Overall effect of TCDD and abnormal sperm (%); C: Overall effect of TCDD and anogenital distance (mm); **(D)**: Overall effect of TCDD and relative anogenital distance (%body length); **(E)**: Overall effect of TCDD and seminal vesicle weight (g); **(F)**: Overall effect of TCDD and prostate weight (g)

Supplementary Table 3 Data details of overall effects. **(A)**: Overall effect of TCDD and relative ventral prostate weight (%body weight); **(B)**: Overall effect of TCDD and relative seminal vesicle weight (%body weight); **(C)**: Overall effect of TCDD and Testis Weight (g); **(D)**: Overall effect of TCDD and Epididymis weight (g)

Supplementary Table 4 Data details of overall effects. **(A)**: Overall effect of TCDD and Testes Weight (g); **(B)**: Overall effect of TCDD and Relative testis weight (%body weight); **(C)**: Overall effect of TCDD and Relative testes weight (%body weight); **(D)**: Overall effect of TCDD and Relative epididymis weight (%body weight)

Supplementary Table 5 Data details of Subgroup analysis. **(A)**: Effect of TCDD and testis weight (g) by Species Subgroups; **(B)**: Effect of TCDD and testis weight (g) by Species Subgroups; **(C)**: Effect of TCDD and testis weight (g) by Exposure Windows Subgroups

Supplementary Table 6 Data details of Subgroup analysis. **(A)**: Effect of TCDD and testes weight (g) by Species Subgroups; **(B)**: Effect of TCDD and testes weight (g) by Exposure Windows Subgroups; **(C)**: Effect of TCDD and Testis Weight (g) by Dosage Levels Subgroups

Supplementary Table 7 Data details of Subgroup analysis. **(A)**: Effect of TCDD and relative testis weight (%body weight) by Species Subgroups; **(B)**: Effect of TCDD and relative testis weight (%body weight) by Exposure Windows Subgroups; **(C)**: Effect of TCDD and relative testis weight (%body weight) by Dosage Levels Subgroups

Supplementary Table 8 Data details of Subgroup analysis. **(A)**: Effect of TCDD and Relative testes weight (%body weight) by Species Subgroups; **(B)**: Effect of TCDD and Relative testes weight (%body weight) by Exposure Windows Subgroups; **(C)**: Effect of TCDD and Relative testes weight (%body weight) by Dosage Levels Subgroups

Supplementary Table 9 Data details of Subgroup analysis. **(A)**: Effect of TCDD and epididymis weight (g) by Species Subgroups; **(B)**: Effect of TCDD and epididymis weight (g) by Exposure Windows Subgroups; **(C)**: Effect of TCDD and epididymis weight (g) by Dosage Levels Subgroups

Supplementary Table 10 Data details of Subgroup analysis. **(A)**: Effect of TCDD and relative epididymis weight (%body weight) by Species Subgroups; **(B)**: Effect of TCDD and relative epididymis weight (%body weight) by Exposure Windows Subgroups; C: Effect of TCDD and relative epididymis weight (%body weight) by Dosage Levels Subgroups

Supplementary Table 11 Data details of Subgroup analysis. **(A)**: Effect of TCDD and ventral prostate weight (g) by Species Subgroups; **(B)**: Effect of TCDD and ventral prostate weight (g) by Exposure Windows Subgroups; **(C)**: Effect of TCDD and ventral prostate weight (g) by Dosage Levels Subgroups

Supplementary Table 12 Data details of Subgroup analysis. **(A)**: Effect of TCDD and prostate weight (g) by Species Subgroups; **(B)**: Effect of TCDD and prostate weight (g) by Exposure Windows Subgroups; **(C)**: Effect of TCDD and prostate weight (g) by Dosage Levels Subgroups

Supplementary Table 13 Data details of Subgroup analysis. **(A)**: Effect of TCDD and relative ventral prostate weight (%body weight) by Species Subgroups; **(B)**: Effect of TCDD and relative ventral prostate weight (%body weight) by Exposure Windows Subgroups; **(C)**: Effect of TCDD and relative ventral prostate weight (%body weight) by Dosage Levels Subgroups

Supplementary Table 14 Data details of Subgroup analysis. **(A)**: Effect of TCDD and seminal vesicle weight (g) by Species Subgroups; **(B)**: Effect of TCDD and seminal vesicle weight (g) by Exposure Windows Subgroups; **(C)**: Effect of TCDD and seminal vesicle weight (g) by Dosage Levels Subgroups

Supplementary Table 15 Data details of Subgroup analysis. **(A)**: Effect of TCDD and anogenital distance (mm) by Species Subgroups; **(B)**: Effect of TCDD and anogenital distance (mm) by Exposure Windows Subgroups; **(C)**: Effect of TCDD and anogenital distance (mm) by Dosage Levels Subgroups

Supplementary Table 16 Data details of Subgroup analysis. **(A)**: Effect of TCDD and relative anogenital distance (%body length) by Species Subgroups; **(B)**: Effect of TCDD and relative anogenital distance (%body length) by Exposure Windows Subgroups; **(C)**: Effect of TCDD and relative anogenital distance (%body length) by Dosage Levels Subgroups

Supplementary Table 17 Data details of Subgroup analysis. **(A)**: Effect of TCDD and sperm count (×10⁶) by Species Subgroups; **(B)**: Effect of TCDD and sperm count (×10⁶) by Exposure Windows Subgroups; **(C)**: Effect of TCDD and sperm count (×10⁶) by Dosage Levels Subgroups

Supplementary Table 18 Data details of Subgroup analysis. **(A)**: Effect of TCDD and daily sperm production (×10⁶) by Species Subgroups; **(B)**: Effect of TCDD and daily sperm production (×10⁶) by Exposure Windows Subgroups; **(C)**: Effect of TCDD and daily sperm production (×10⁶) by Dosage Levels Subgroups

Supplementary Table 19 Data details of Subgroup analysis. **(A)**: Effect of TCDD and serum testosterone (ng/ml) by Species Subgroups; **(B)**: Effect of TCDD and serum testosterone (ng/ml) by Exposure Windows Subgroups; **(C)**: Effect of TCDD and serum testosterone (ng/ml) by Dosage Levels Subgroups

Supplementary Table 20 Data details of Subgroup analysis. **(A)**: Effect of TCDD and sperm motility (%) by Species Subgroups; **(B)**: Effect of TCDD and sperm motility (%) by Exposure Windows Subgroups; **(C)**: Effect of TCDD and sperm motility (%) by Dosage Levels Subgroups

Supplementary Table 21 Data details of Subgroup analysis. **(A)**: Effect of TCDD and abnormal sperm (%) by Species Subgroups; **(B)**: Effect of TCDD and abnormal sperm (%) by Exposure Windows Subgroups; **(C)**: Effect of TCDD and abnormal sperm (%) by Dosage Levels Subgroups

Supplementary Table 22 Data details of Subgroup analysis. **(A)**: Effect of TCDD and relative seminal vesicle weight (%body weight) by Species Subgroups; **(B)**: Effect of TCDD and relative seminal vesicle weight (%body weight) by Exposure Windows Subgroups; **(C)**: Effect of TCDD and relative seminal vesicle weight (%body weight) by Dosage Levels Subgroups

Supplementary Table 23 Outcomes of Egger’s test and results of the adjusted details. **(A)**: Details of Egger’s test. Publication bias of testis weight (g) is detected; **(B)**: Overall effect of TCDD and testis weight (g) after 1 comparison with the lowest weight dropped; **(C)**: Details of Egger’s test for testis weight (g) after 1 comparison with the lowest weight dropped. No Publication Bias is detected; **(D)**: Details of Egger’s test. Publication Bias of testes weight (g) is detected; **(E)**: Overall effect of TCDD and testes weight (g) after 1 comparison with the lowest weight dropped; **(F)**: Details of Egger’s test for testes weight (g) after 1 comparison with the lowest weight dropped. No significant Publication Bias detected; **(G)**: Details of Egger’s test. Publication Bias of relative testis weight (%body weight) is detected; **(H)**: Overall effect of TCDD and relative testis weight (%body weight) after 50% comparisons with the lowest weight dropped; **(I)**: Details of Egger’s test for relative testis weight (%body weight) after 50% comparisons with the lowest weight dropped. Publication Bias is detected; **(J)**: Details of Egger’s test. No Publication Bias of relative testes weight (%body weight) is detected; **(K)**: Details of Egger’s test. Publication Bias of epididymis weight (g) is detected; **(L)**: Overall effect of TCDD and epididymis weight (g) after 32 comparisons with the lowest weight dropped; **(M)**: Details of Egger’s test for epididymis weight (g) after 32 comparisons with the lowest weight dropped. No Publication Bias is detected; **(N)**: Details of Egger’s test. No significant Publication Bias of relative epididymis weight (%body weight) is detected; **(O)**: Details of Egger’s test. No Publication Bias of ventral prostate weight (g) is detected; **(P)**: Details of Egger’s test. Publication Bias of prostate weight (g) is detected; **(Q)**: Overall effect of TCDD and prostate weight (g) after 5 comparisons with the lowest weight dropped; **(R)**: Details of Egger’s test for prostate weight (g) after 5 comparisons with the lowest weight dropped. No Publication Bias is detected; **(S)**: Details of Egger’s test. No Publication Bias of relative ventral prostate weight (%body weight) is detected.

Supplementary Table 24 Outcomes of Egger’s test and results of the adjusted details. **(A)**: Details of Egger’s test. Publication Bias of testes weight (g) is detected; **(B)**: Overall effect of TCDD and testes weight (g) after 50% comparisons with the lowest weight dropped; **(C)**: Details of Egger’s test for testes weight (g) after 50% comparisons with the lowest weight dropped. Publication Bias is detected; **(D)**: Details of Egger’s test. Publication Bias of anogenital distance (mm) is detected; **(E)**: Overall effect of TCDD and anogenital distance (mm) after the 50% comparisons with the lowest weight dropped; **(F)**: Details of Egger’s test for anogenital distance (mm) after the 50% comparisons with the lowest weight dropped. Publication Bias is detected; **(G)**: Details of Egger’s test. No Publication Bias of relative anogenital distance (%body length) is detected; **(H)**: Details of Egger’s test. Publication Bias of sperm count (×10⁶) is detected; **(I)**: Overall effect of TCDD and sperm count (×10⁶) after 16 comparisons with the lowest weight dropped; **(J)**: Details of Egger’s test for sperm count (×10⁶) after 16 comparisons with the lowest weight dropped. No Publication Bias is detected; **(K)**: Details of Egger’s test. Publication Bias of daily sperm production (×10⁶) is detected; **(L)**: Overall effect of TCDD and daily sperm production (×10⁶) after the 50% comparisons with the lowest weight dropped; **(M)**: Details of Egger’s test for daily sperm production (×10⁶) after the 50% comparisons with the lowest weight dropped. Publication Bias is detected; **(N)**: Details of Egger’s test. No Publication Bias of serum testosterone (ng/ml) is detected; **(O)**: Details of Egger’s test. No Publication Bias of sperm motility (%) is detected; **(P)**: Details of Egger’s test. No Publication Bias of abnormal sperm (%) is detected; **(Q)**: Details of Egger’s test. Publication Bias of relative seminal vesicle weight (%body weight) is detected; **(R)**: Overall effect of TCDD and relative seminal vesicle weight (%body weight) after the 50% comparisons with the lowest weight dropped; **(S)**: Details of Egger’s test for relative seminal vesicle weight (%body weight) after the 50% comparisons with the lowest weight dropped. Publication Bias is detected
