# Supplementary material for: Animal Toxicology Studies on the Male Reproductive Effects of 2,3,7,8-Tetrachlorodibenzo-p-Dioxin: Data Analysis and Health Effects Evaluation
Source: Front Endocrinol (Lausanne). 2021 Nov 3;12:696106. doi: 10.3389/fendo.2021.696106 (PMC8595279; doi:10.3389/fendo.2021.696106)
Supplement: Supplementary Figure 1 — Forest plots of overall effects. (A): Overall effect of TCDD and sperm motility (%); (B): Overall effect of TCDD and abnormal sperm (%); (C): Overall effect of TCDD and anogenital distance(mm); (D): Overall effect of TCDD and relative anogenital distance (%body length); (E): Overall effect of TCDD and seminal vesicle weight (g); (F): Overall effect of TCDD and prostate weight (g) [file DataSheet_1.zip › DATA sheet 1/Supplementary Figure 15.pdf]

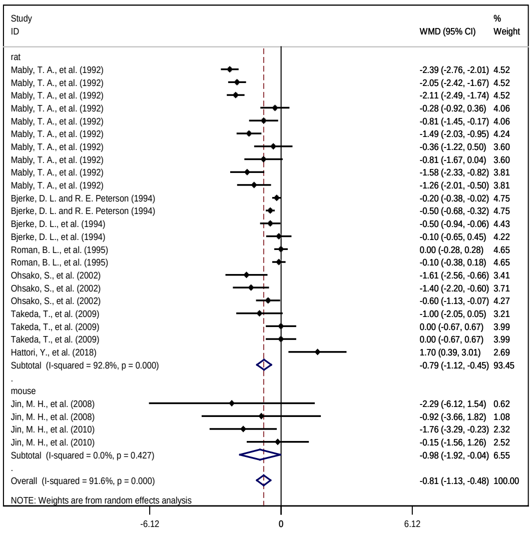
Study
ID
WMD (95% CI)
% Weight
rat
Mably, T. A., et al. (1992) -2.39 (-2.76, -2.01) 4.52
Mably, T. A., et al. (1992) -2.05 (-2.42, -1.67) 4.52
Mably, T. A., et al. (1992) -2.11 (-2.49, -1.74) 4.52
Mably, T. A., et al. (1992) -0.28 (-0.92, 0.36) 4.06
Mably, T. A., et al. (1992) -0.81 (-1.45, -0.17) 4.06
Mably, T. A., et al. (1992) -1.49 (-2.03, -0.95) 4.24
Mably, T. A., et al. (1992) -0.36 (-1.22, 0.50) 3.60
Mably, T. A., et al. (1992) -0.81 (-1.67, 0.04) 3.60
Mably, T. A., et al. (1992) -1.58 (-2.33, -0.82) 3.81
Mably, T. A., et al. (1992) -1.26 (-2.01, -0.50) 3.81
Bjerke, D. L. and R. E. Peterson (1994) -0.20 (-0.38, -0.02) 4.75
Bjerke, D. L. and R. E. Peterson (1994) -0.50 (-0.68, -0.32) 4.75
Bjerke, D. L., et al. (1994) -0.50 (-0.94, -0.06) 4.43
Bjerke, D. L., et al. (1994) -0.10 (-0.65, 0.45) 4.22
Roman, B. L., et al. (1995) 0.00 (-0.28, 0.28) 4.65
Roman, B. L., et al. (1995) -0.10 (-0.38, 0.18) 4.65
Ohsako, S., et al. (2002) -1.61 (-2.56, -0.66) 3.41
Ohsako, S., et al. (2002) -1.40 (-2.20, -0.60) 3.71
Ohsako, S., et al. (2002) -0.60 (-1.13, -0.07) 4.27
Takeda, T., et al. (2009) -1.00 (-2.05, 0.05) 3.21
Takeda, T., et al. (2009) 0.00 (-0.67, 0.67) 3.99
Takeda, T., et al. (2009) 0.00 (-0.67, 0.67) 3.99
Hattori, Y., et al. (2018) 1.70 (0.39, 3.01) 2.69
Subtotal (I-squared = 92.8%, p = 0.000) -0.79 (-1.12, -0.45) 93.45
.
mouse
Jin, M. H., et al. (2008) -2.29 (-6.12, 1.54) 0.62
Jin, M. H., et al. (2008) -0.92 (-3.66, 1.82) 1.08
Jin, M. H., et al. (2010) -1.76 (-3.29, -0.23) 2.32
Jin, M. H., et al. (2010) -0.15 (-1.56, 1.26) 2.52
Subtotal (I-squared = 0.0%, p = 0.427) -0.98 (-1.92, -0.04) 6.55
.
Overall (I-squared = 91.6%, p = 0.000) -0.81 (-1.13, -0.48) 100.00
NOTE: Weights are from random effects analysis
-6.12
0
6.12

A

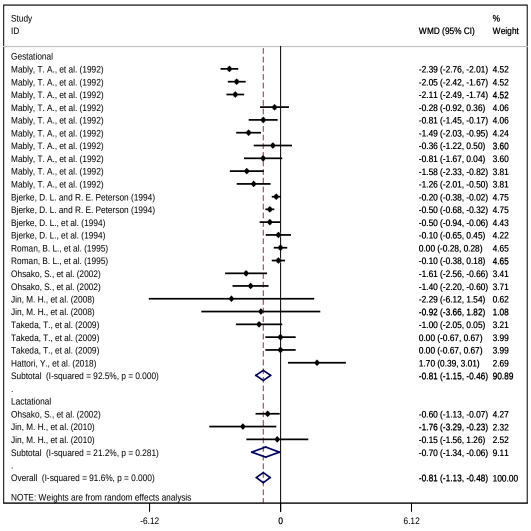
Study
ID
WMD (95% CI)
% Weight
Gestational
Mably, T. A., et al. (1992) -2.39 (-2.76, -2.01) 4.52
Mably, T. A., et al. (1992) -2.05 (-2.42, -1.67) 4.52
Mably, T. A., et al. (1992) -2.11 (-2.49, -1.74) 4.52
Mably, T. A., et al. (1992) -0.28 (-0.92, 0.36) 4.06
Mably, T. A., et al. (1992) -0.81 (-1.45, -0.17) 4.06
Mably, T. A., et al. (1992) -1.49 (-2.03, -0.95) 4.24
Mably, T. A., et al. (1992) -0.36 (-1.22, 0.50) 3.60
Mably, T. A., et al. (1992) -0.81 (-1.67, 0.04) 3.60
Mably, T. A., et al. (1992) -1.58 (-2.33, -0.82) 3.81
Mably, T. A., et al. (1992) -1.26 (-2.01, -0.50) 3.81
Bjerke, D. L. and R. E. Peterson (1994) -0.20 (-0.38, -0.02) 4.75
Bjerke, D. L. and R. E. Peterson (1994) -0.50 (-0.68, -0.32) 4.75
Bjerke, D. L., et al. (1994) -0.50 (-0.94, -0.06) 4.43
Bjerke, D. L., et al. (1994) -0.10 (-0.65, 0.45) 4.22
Roman, B. L., et al. (1995) 0.00 (-0.28, 0.28) 4.65
Roman, B. L., et al. (1995) -0.10 (-0.38, 0.18) 4.65
Ohsako, S., et al. (2002) -1.61 (-2.56, -0.66) 3.41
Ohsako, S., et al. (2002) -1.40 (-2.20, -0.60) 3.71
Jin, M. H., et al. (2008) -2.29 (-6.12, 1.54) 0.62
Jin, M. H., et al. (2008) -0.92 (-3.66, 1.82) 1.08
Takeda, T., et al. (2009) -1.00 (-2.05, 0.05) 3.21
Takeda, T., et al. (2009) 0.00 (-0.67, 0.67) 3.99
Takeda, T., et al. (2009) 0.00 (-0.67, 0.67) 3.99
Hattori, Y., et al. (2018) 1.70 (0.39, 3.01) 2.69
Subtotal (I-squared = 92.5%, p = 0.000) -0.81 (-1.15, -0.46) 90.89
.
Lactational
Ohsako, S., et al. (2002) -0.60 (-1.13, -0.07) 4.27
Jin, M. H., et al. (2010) -1.76 (-3.29, -0.23) 2.32
Jin, M. H., et al. (2010) -0.15 (-1.56, 1.26) 2.52
Subtotal (I-squared = 21.2%, p = 0.281) -0.70 (-1.34, -0.06) 9.11
.
Overall (I-squared = 91.6%, p = 0.000) -0.81 (-1.13, -0.48) 100.00
NOTE: Weights are from random effects analysis
-6.12
0
6.12

B

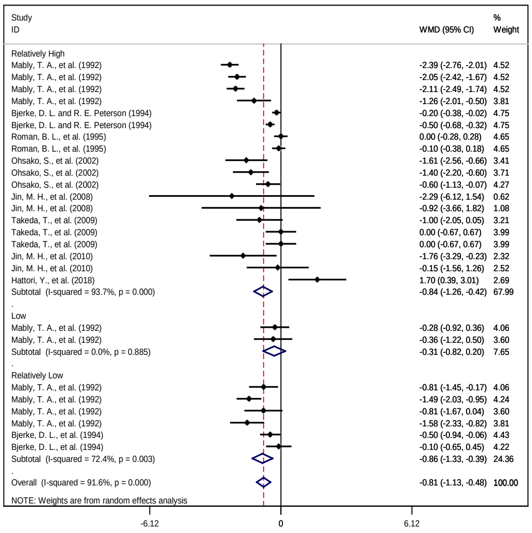
Study
ID
WMD (95% CI)
% Weight
Relatively High
Mably, T. A., et al. (1992) -2.39 (-2.76, -2.01) 4.52
Mably, T. A., et al. (1992) -2.05 (-2.42, -1.67) 4.52
Mably, T. A., et al. (1992) -2.11 (-2.49, -1.74) 4.52
Mably, T. A., et al. (1992) -1.26 (-2.01, -0.50) 3.81
Bjerke, D. L. and R. E. Peterson (1994) -0.20 (-0.38, -0.02) 4.75
Bjerke, D. L. and R. E. Peterson (1994) -0.50 (-0.68, -0.32) 4.75
Roman, B. L., et al. (1995) 0.00 (-0.28, 0.28) 4.65
Roman, B. L., et al. (1995) -0.10 (-0.38, 0.18) 4.65
Ohsako, S., et al. (2002) -1.61 (-2.56, -0.66) 3.41
Ohsako, S., et al. (2002) -1.40 (-2.20, -0.60) 3.71
Ohsako, S., et al. (2002) -0.60 (-1.13, -0.07) 4.27
Jin, M. H., et al. (2008) -2.29 (-6.12, 1.54) 0.62
Jin, M. H., et al. (2008) -0.92 (-3.66, 1.82) 1.08
Takeda, T., et al. (2009) -1.00 (-2.05, 0.05) 3.21
Takeda, T., et al. (2009) 0.00 (-0.67, 0.67) 3.99
Takeda, T., et al. (2009) 0.00 (-0.67, 0.67) 3.99
Jin, M. H., et al. (2010) -1.76 (-3.29, -0.23) 2.32
Jin, M. H., et al. (2010) -0.15 (-1.56, 1.26) 2.52
Hattori, Y., et al. (2018) 1.70 (0.39, 3.01) 2.69
Subtotal (I-squared = 93.7%, p = 0.000) -0.84 (-1.26, -0.42) 67.99
.
Low
Mably, T. A., et al. (1992) -0.28 (-0.92, 0.36) 4.06
Mably, T. A., et al. (1992) -0.36 (-1.22, 0.50) 3.60
Subtotal (I-squared = 0.0%, p = 0.885) -0.31 (-0.82, 0.20) 7.65
.
Relatively Low
Mably, T. A., et al. (1992) -0.81 (-1.45, -0.17) 4.06
Mably, T. A., et al. (1992) -1.49 (-2.03, -0.95) 4.24
Mably, T. A., et al. (1992) -0.81 (-1.67, 0.04) 3.60
Mably, T. A., et al. (1992) -1.58 (-2.33, -0.82) 3.81
Bjerke, D. L., et al. (1994) -0.50 (-0.94, -0.06) 4.43
Bjerke, D. L., et al. (1994) -0.10 (-0.65, 0.45) 4.22
Subtotal (I-squared = 72.4%, p = 0.003) -0.86 (-1.33, -0.39) 24.36
.
Overall (I-squared = 91.6%, p = 0.000) -0.81 (-1.13, -0.48) 100.00
NOTE: Weights are from random effects analysis
-6.12
0
6.12

C
