# Supplementary figures and images for: Animal Toxicology Studies on the Male Reproductive Effects of 2,3,7,8-Tetrachlorodibenzo-p-Dioxin: Data Analysis and Health Effects Evaluation
Source: Front Endocrinol (Lausanne). 2021 Nov 3;12:696106. doi: 10.3389/fendo.2021.696106 (PMC8595279; doi:10.3389/fendo.2021.696106)

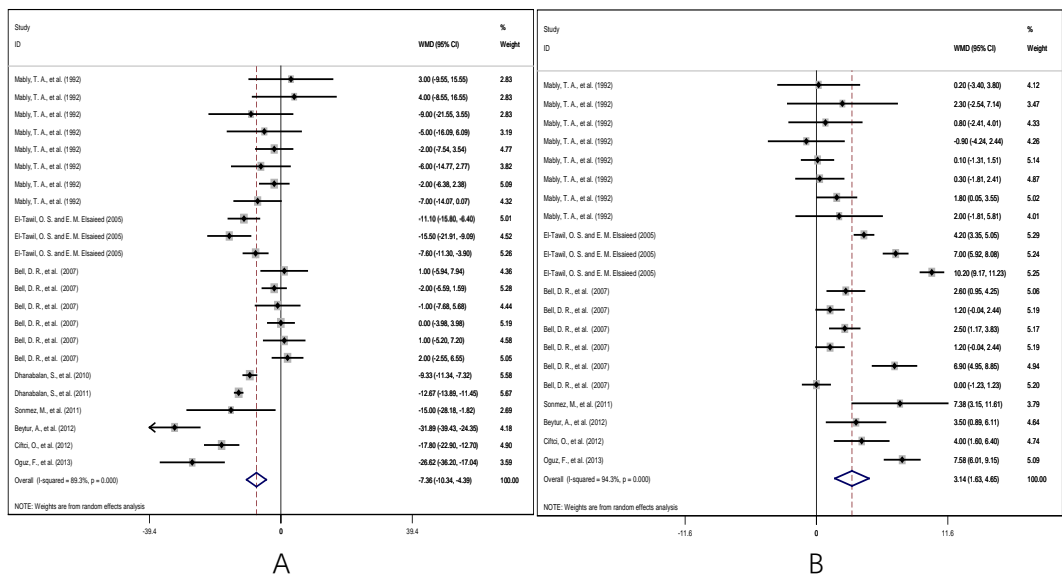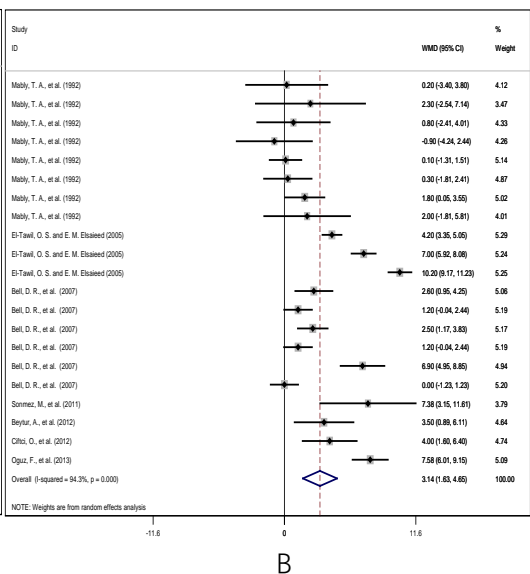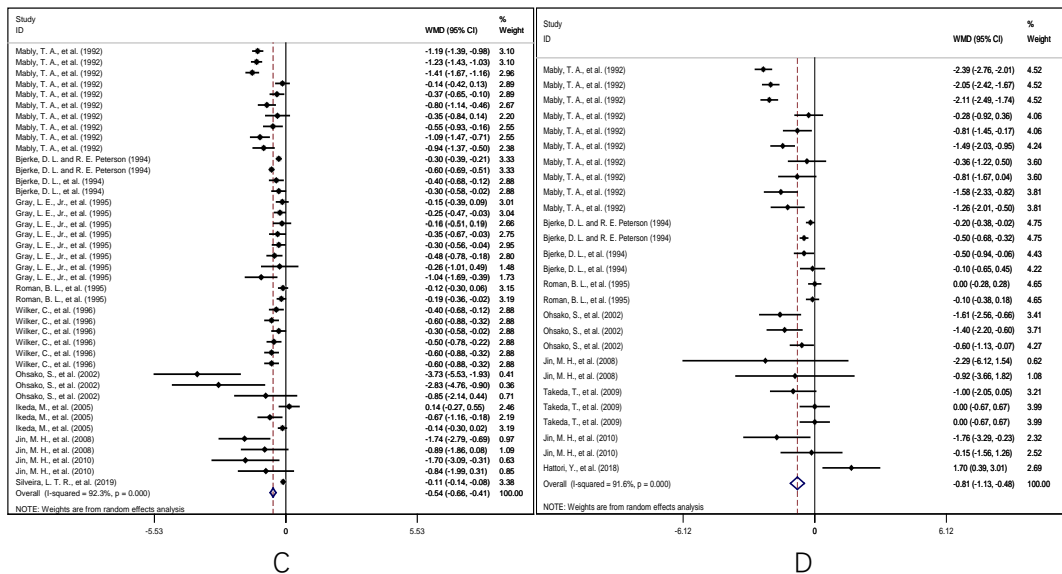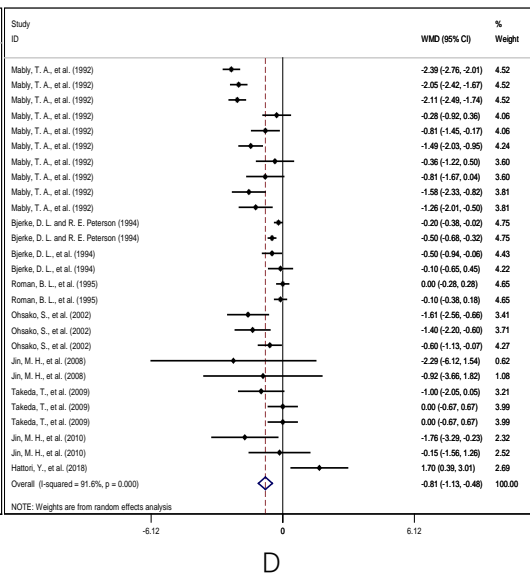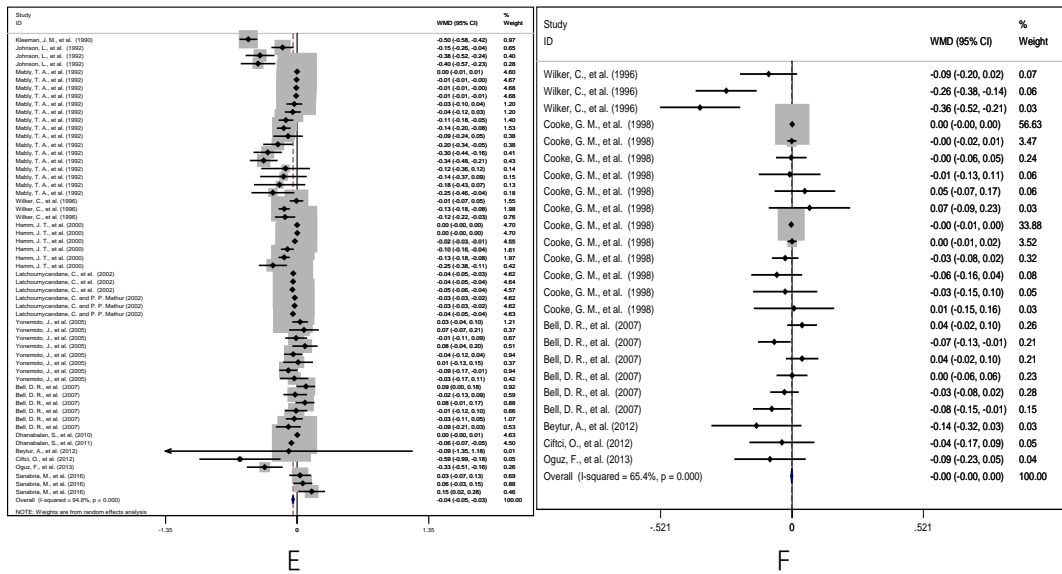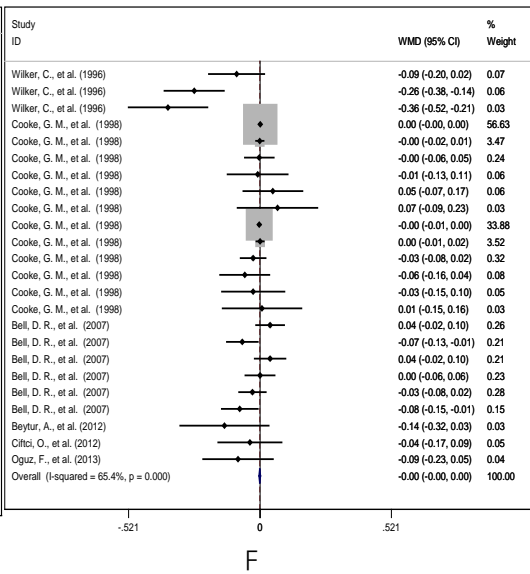

Supplement: Supplementary Figure 1 — Forest plots of overall effects. (A): Overall effect of TCDD and sperm motility (%); (B): Overall effect of TCDD and abnormal sperm (%); (C): Overall effect of TCDD and anogenital distance(mm); (D): Overall effect of TCDD and relative anogenital distance (%body length); (E): Overall effect of TCDD and seminal vesicle weight (g); (F): Overall effect of TCDD and prostate weight (g) [file DataSheet_1.zip › DATA sheet 1/Supplementary Figure 1.pdf]

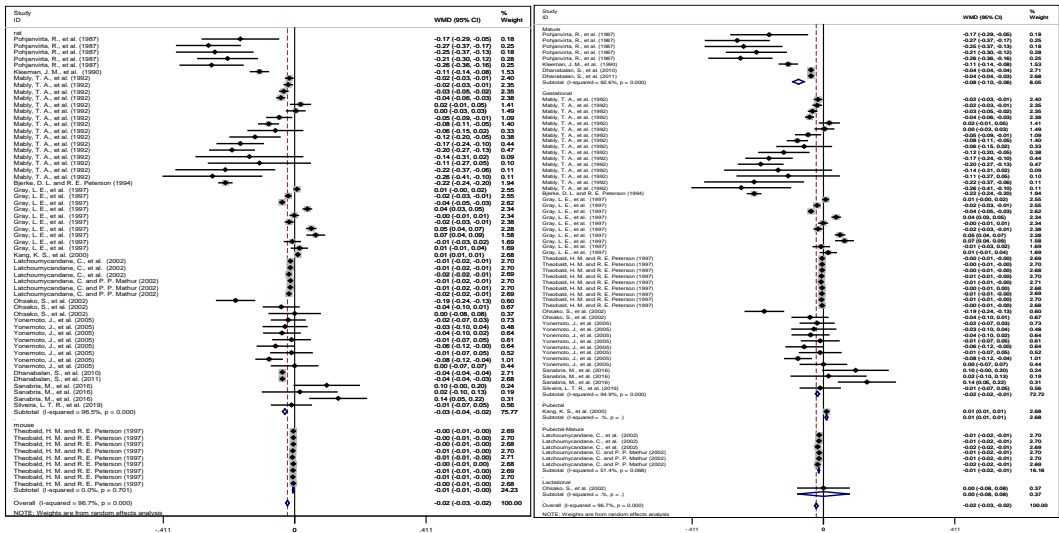

A

B

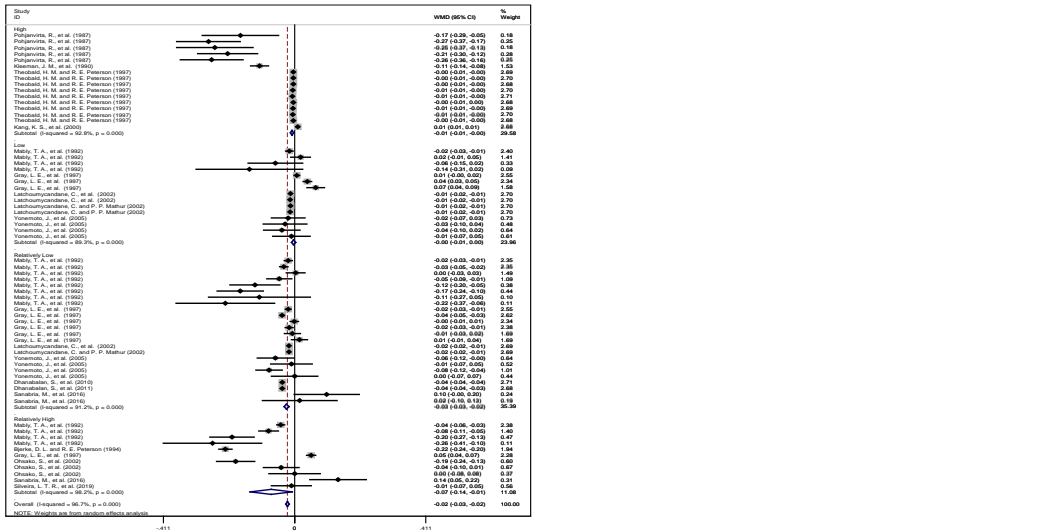

C

Supplement: Supplementary Figure 1 — Forest plots of overall effects. (A): Overall effect of TCDD and sperm motility (%); (B): Overall effect of TCDD and abnormal sperm (%); (C): Overall effect of TCDD and anogenital distance(mm); (D): Overall effect of TCDD and relative anogenital distance (%body length); (E): Overall effect of TCDD and seminal vesicle weight (g); (F): Overall effect of TCDD and prostate weight (g) [file DataSheet_1.zip › DATA sheet 1/Supplementary Figure 10.pdf]

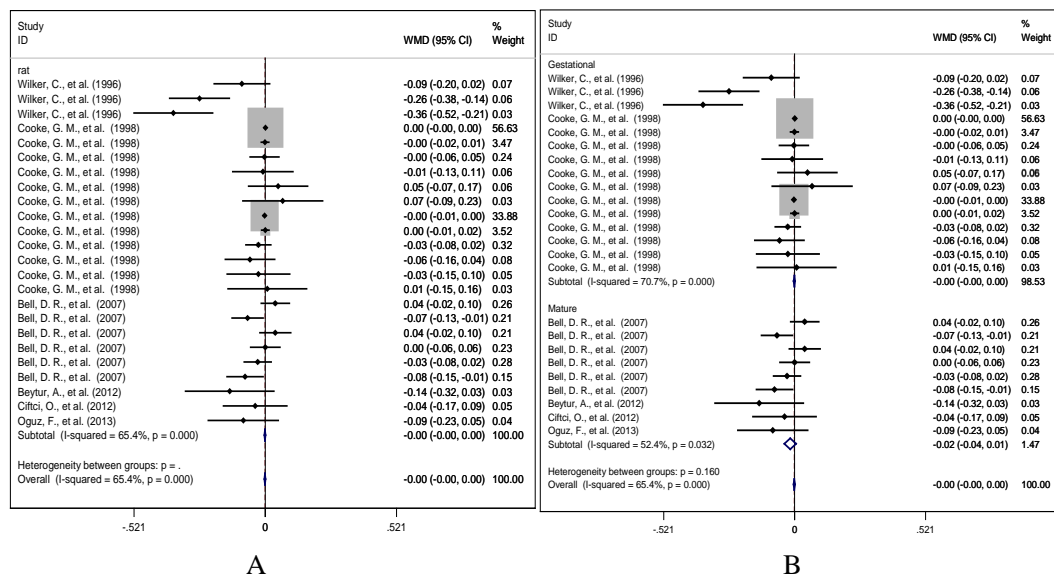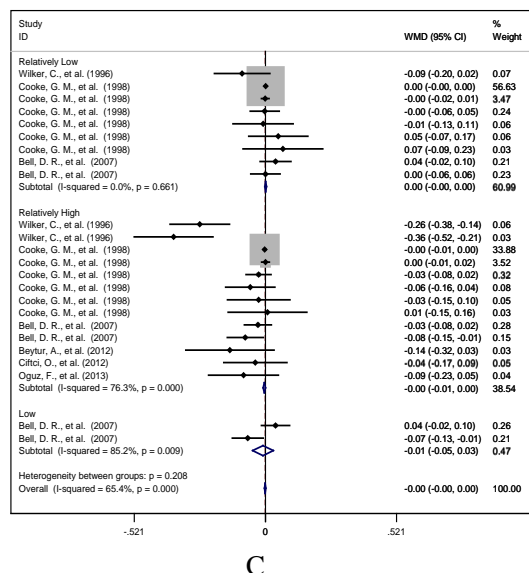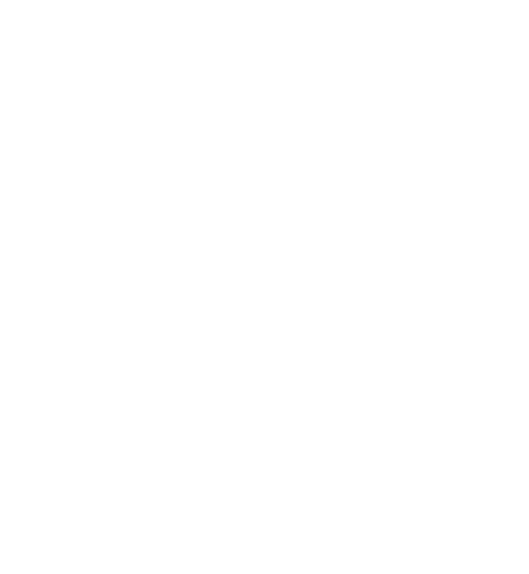

Supplement: Supplementary Figure 1 — Forest plots of overall effects. (A): Overall effect of TCDD and sperm motility (%); (B): Overall effect of TCDD and abnormal sperm (%); (C): Overall effect of TCDD and anogenital distance(mm); (D): Overall effect of TCDD and relative anogenital distance (%body length); (E): Overall effect of TCDD and seminal vesicle weight (g); (F): Overall effect of TCDD and prostate weight (g) [file DataSheet_1.zip › DATA sheet 1/Supplementary Figure 11.pdf]

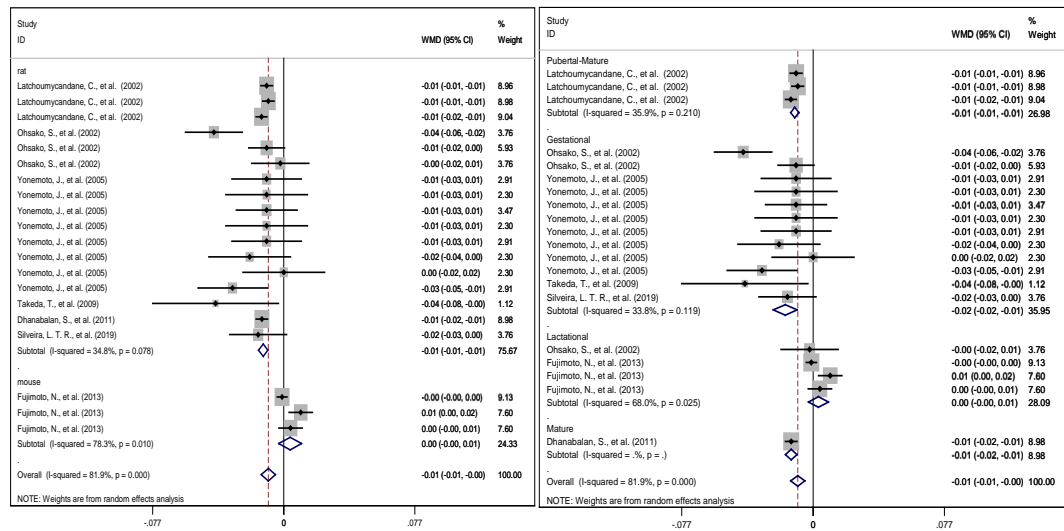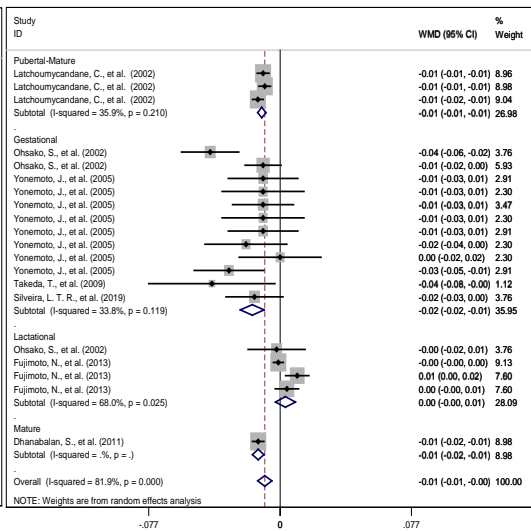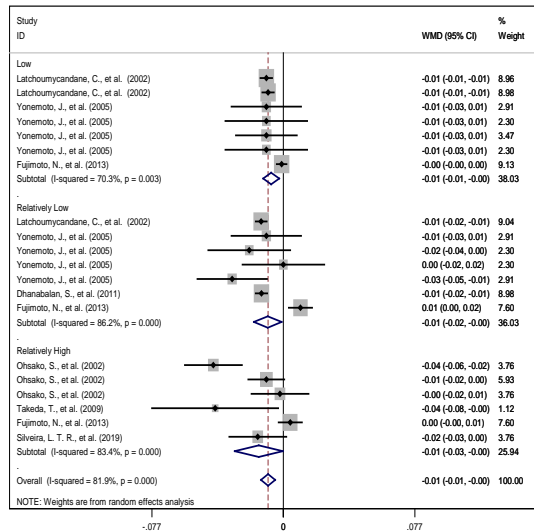

Supplement: Supplementary Figure 1 — Forest plots of overall effects. (A): Overall effect of TCDD and sperm motility (%); (B): Overall effect of TCDD and abnormal sperm (%); (C): Overall effect of TCDD and anogenital distance(mm); (D): Overall effect of TCDD and relative anogenital distance (%body length); (E): Overall effect of TCDD and seminal vesicle weight (g); (F): Overall effect of TCDD and prostate weight (g) [file DataSheet_1.zip › DATA sheet 1/Supplementary Figure 12.pdf]

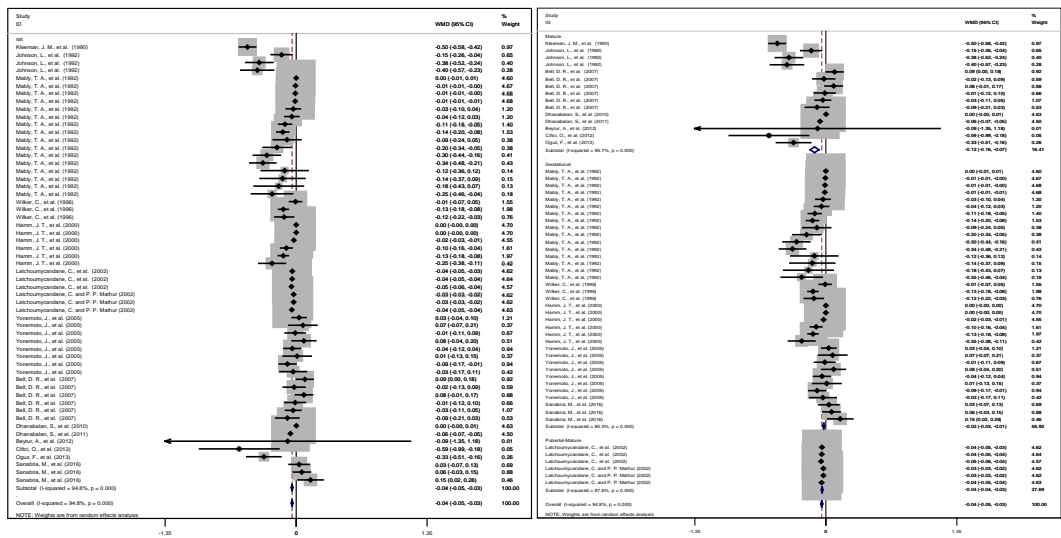

A

B

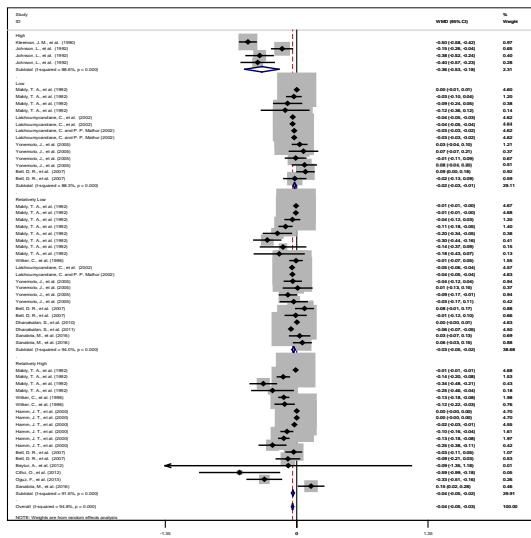

C

Supplement: Supplementary Figure 1 — Forest plots of overall effects. (A): Overall effect of TCDD and sperm motility (%); (B): Overall effect of TCDD and abnormal sperm (%); (C): Overall effect of TCDD and anogenital distance(mm); (D): Overall effect of TCDD and relative anogenital distance (%body length); (E): Overall effect of TCDD and seminal vesicle weight (g); (F): Overall effect of TCDD and prostate weight (g) [file DataSheet_1.zip › DATA sheet 1/Supplementary Figure 13.pdf]

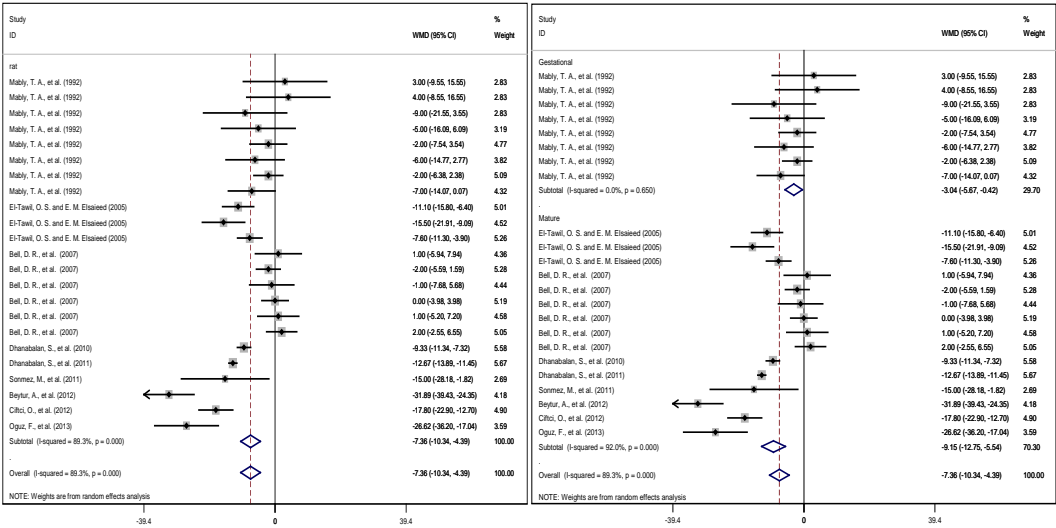

A

B

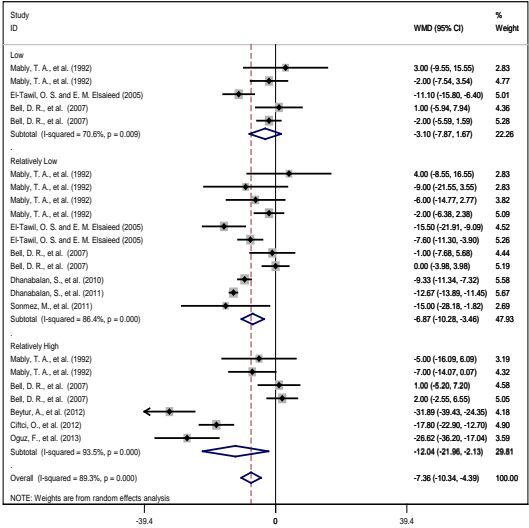

C

Supplement: Supplementary Figure 1 — Forest plots of overall effects. (A): Overall effect of TCDD and sperm motility (%); (B): Overall effect of TCDD and abnormal sperm (%); (C): Overall effect of TCDD and anogenital distance(mm); (D): Overall effect of TCDD and relative anogenital distance (%body length); (E): Overall effect of TCDD and seminal vesicle weight (g); (F): Overall effect of TCDD and prostate weight (g) [file DataSheet_1.zip › DATA sheet 1/Supplementary Figure 19.pdf]

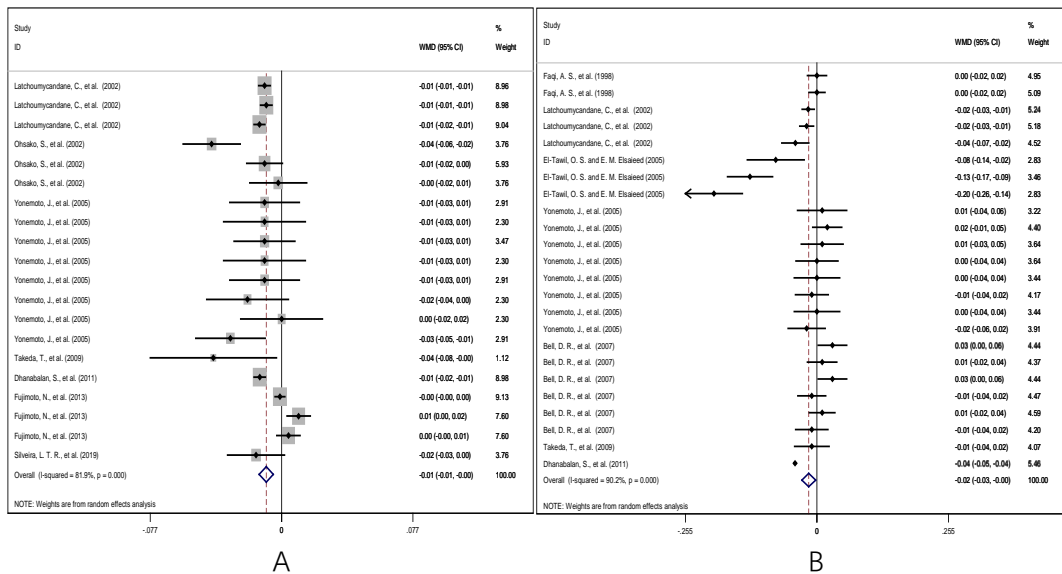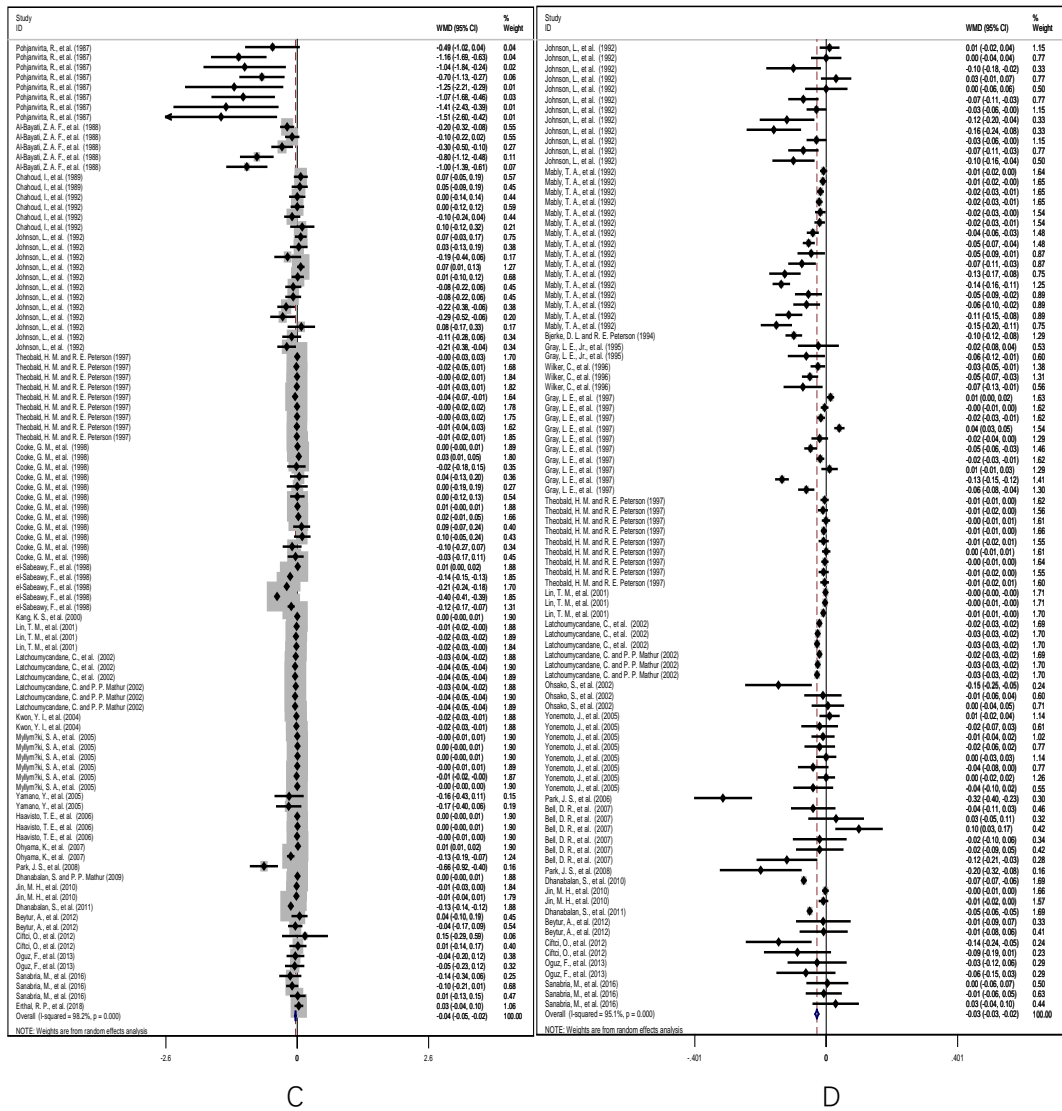

Supplement: Supplementary Figure 1 — Forest plots of overall effects. (A): Overall effect of TCDD and sperm motility (%); (B): Overall effect of TCDD and abnormal sperm (%); (C): Overall effect of TCDD and anogenital distance(mm); (D): Overall effect of TCDD and relative anogenital distance (%body length); (E): Overall effect of TCDD and seminal vesicle weight (g); (F): Overall effect of TCDD and prostate weight (g) [file DataSheet_1.zip › DATA sheet 1/Supplementary Figure 2.pdf]

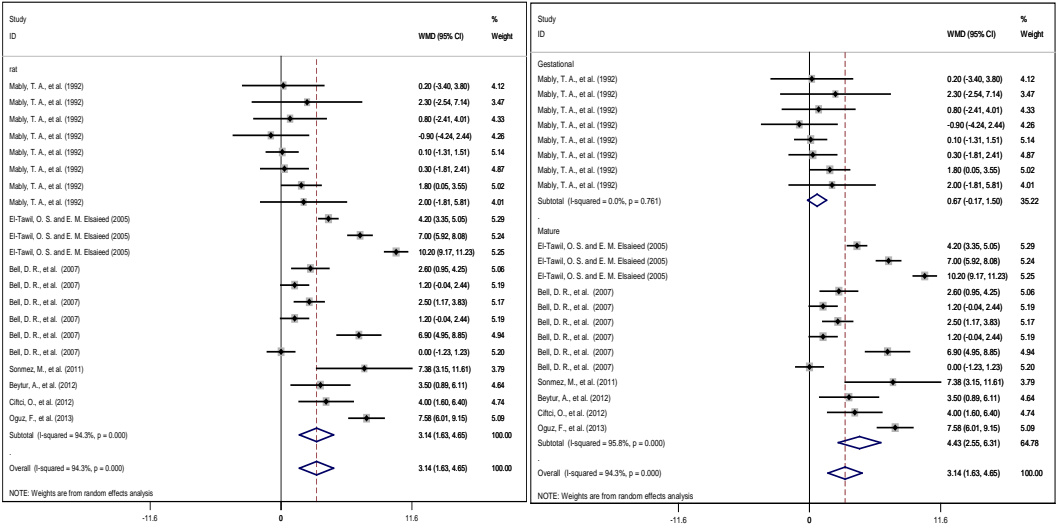

Supplement: Supplementary Figure 1 — Forest plots of overall effects. (A): Overall effect of TCDD and sperm motility (%); (B): Overall effect of TCDD and abnormal sperm (%); (C): Overall effect of TCDD and anogenital distance(mm); (D): Overall effect of TCDD and relative anogenital distance (%body length); (E): Overall effect of TCDD and seminal vesicle weight (g); (F): Overall effect of TCDD and prostate weight (g) [file DataSheet_1.zip › DATA sheet 1/Supplementary Figure 20.pdf]

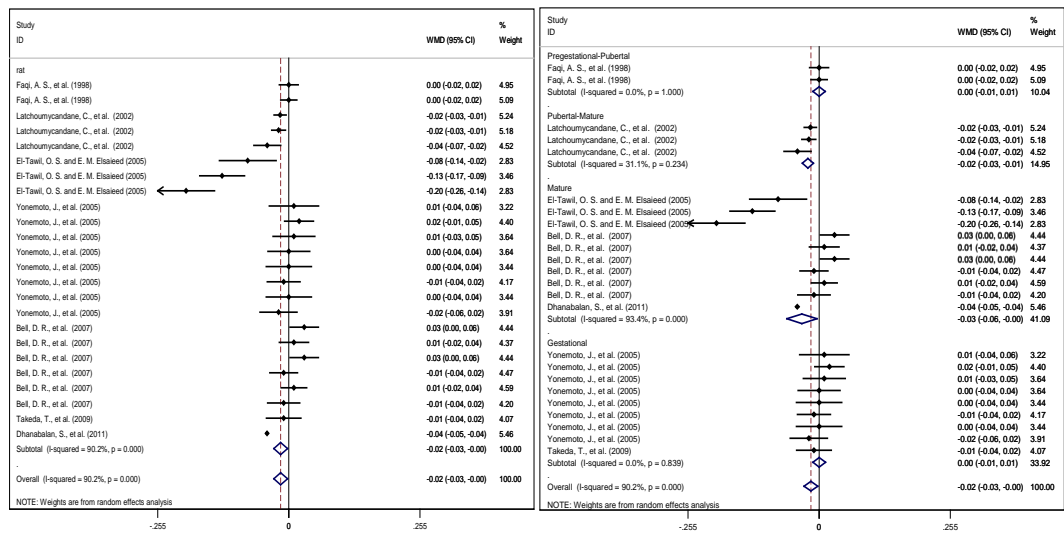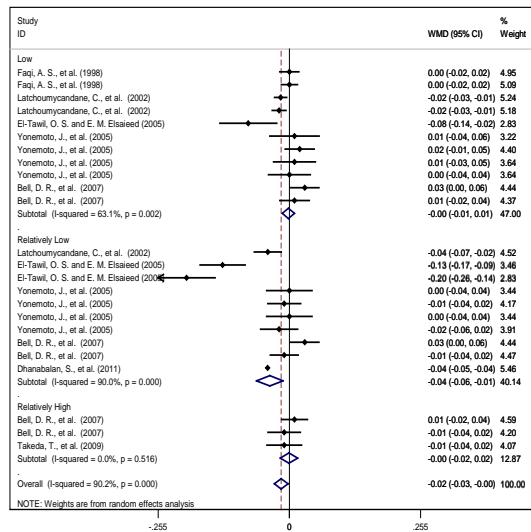

Supplement: Supplementary Figure 1 — Forest plots of overall effects. (A): Overall effect of TCDD and sperm motility (%); (B): Overall effect of TCDD and abnormal sperm (%); (C): Overall effect of TCDD and anogenital distance(mm); (D): Overall effect of TCDD and relative anogenital distance (%body length); (E): Overall effect of TCDD and seminal vesicle weight (g); (F): Overall effect of TCDD and prostate weight (g) [file DataSheet_1.zip › DATA sheet 1/Supplementary Figure 21.pdf]

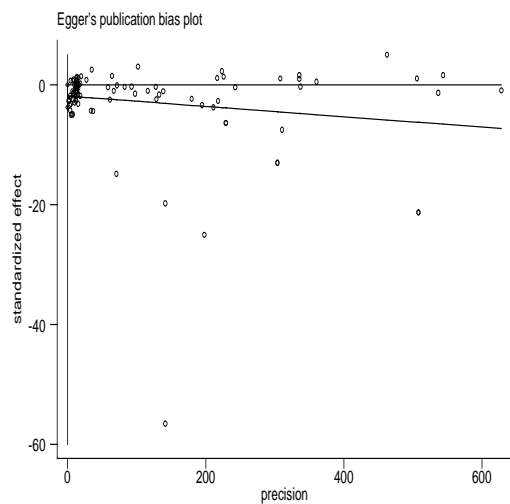

A

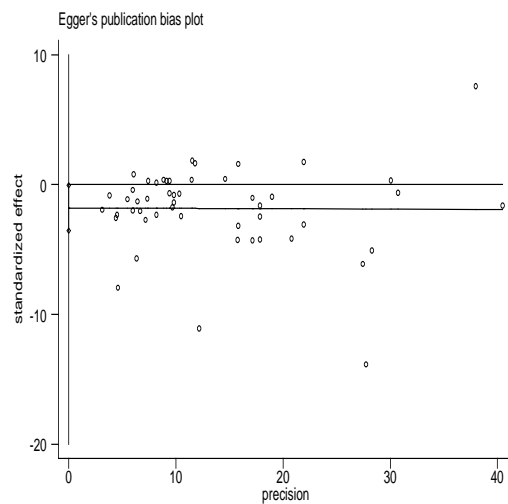

B

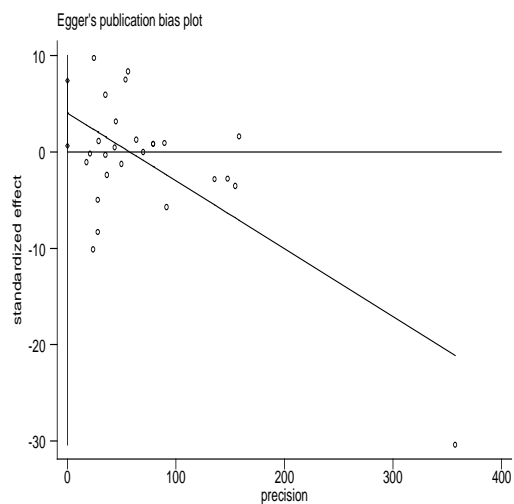

C

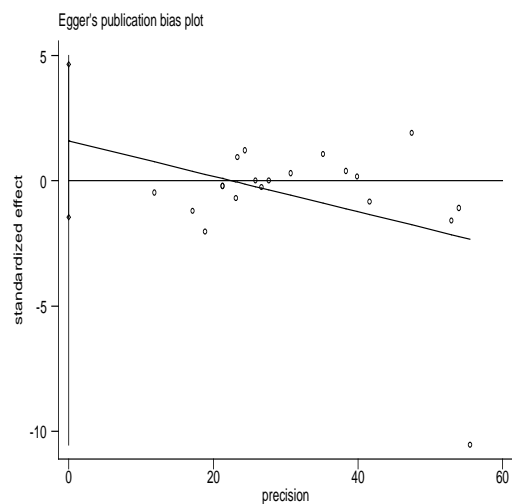

D

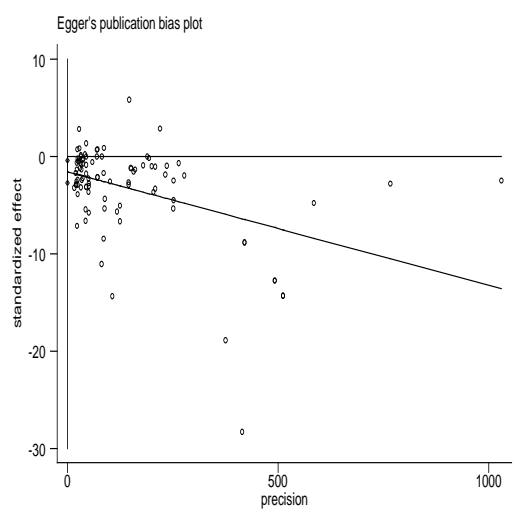

E

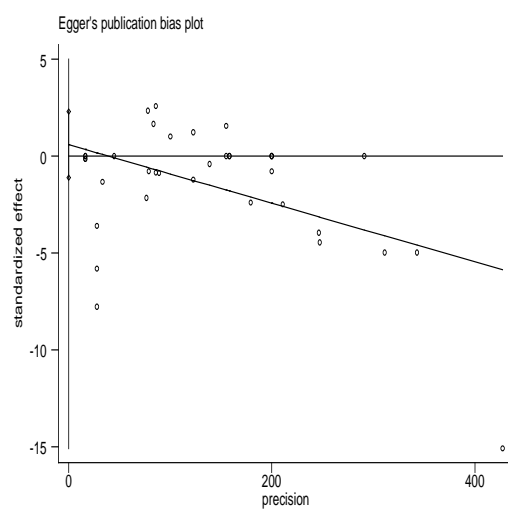

F

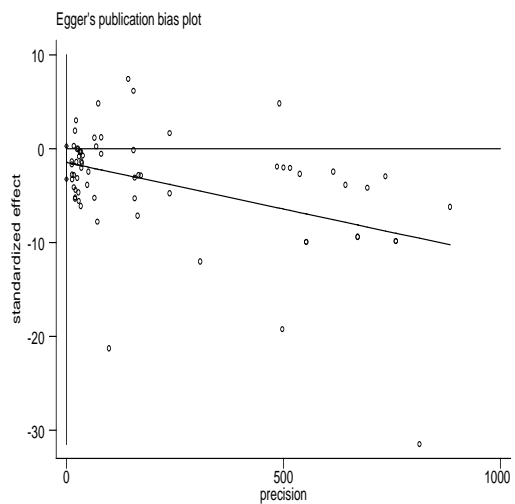

G

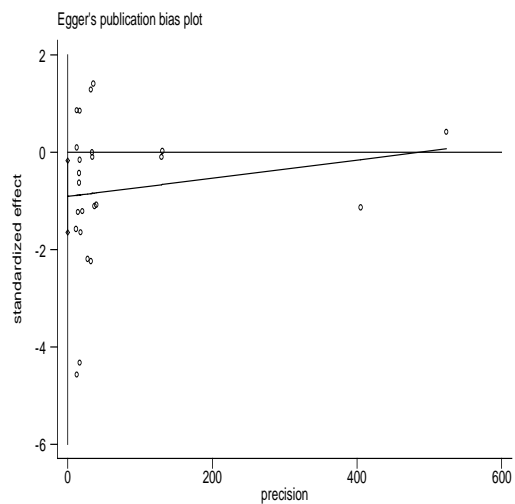

H

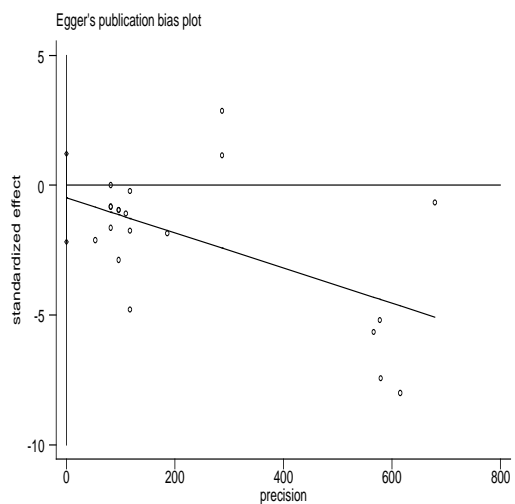

I

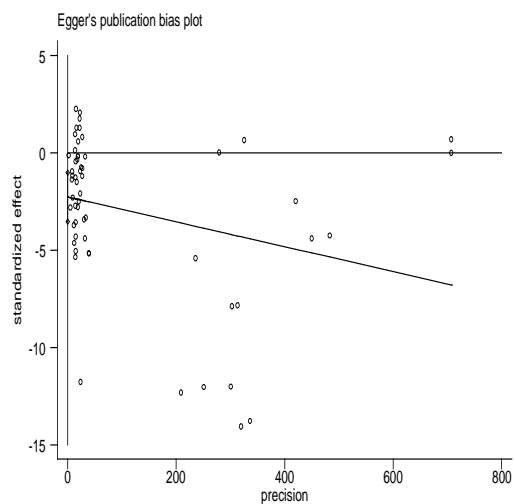

J

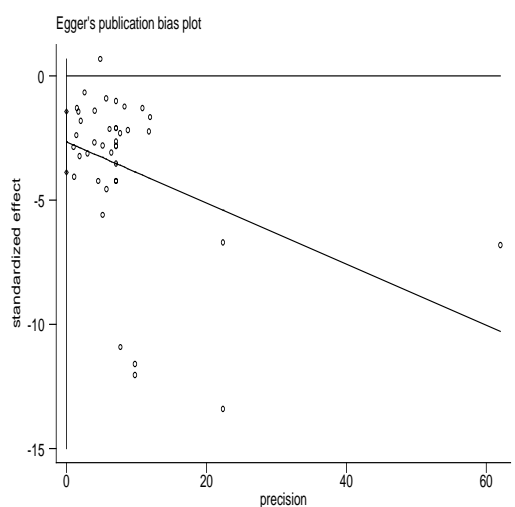

K

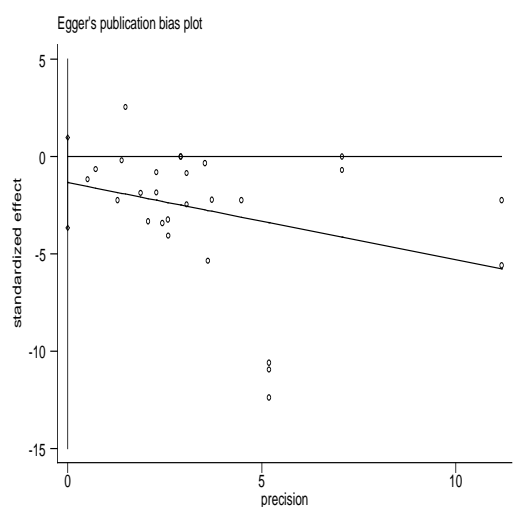

L

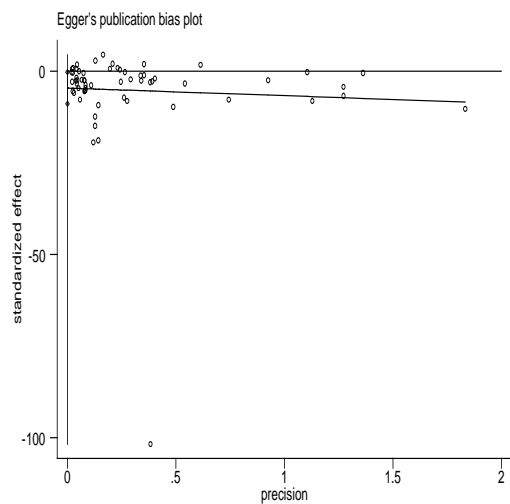

M

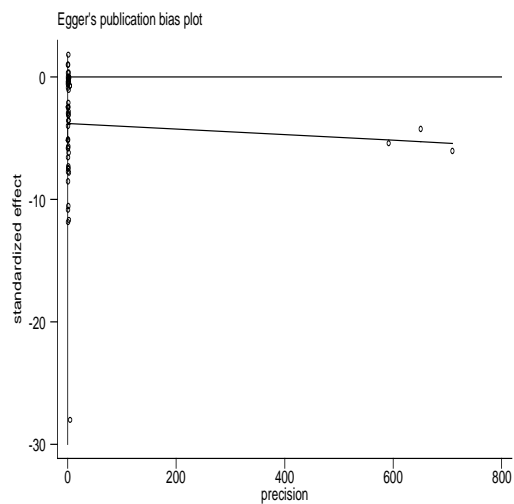

N

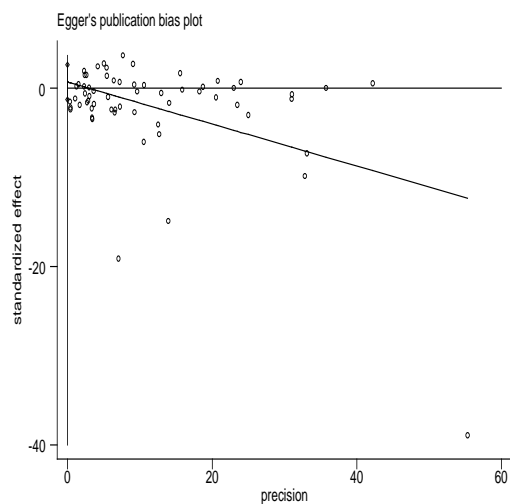

O

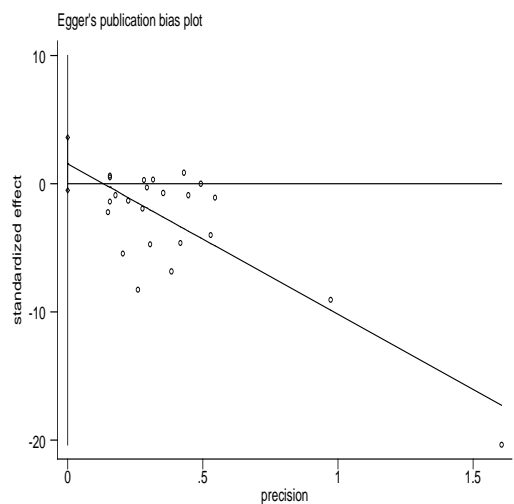

P

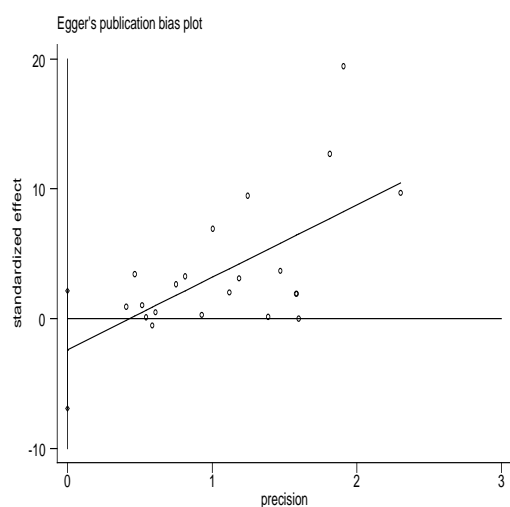

Q

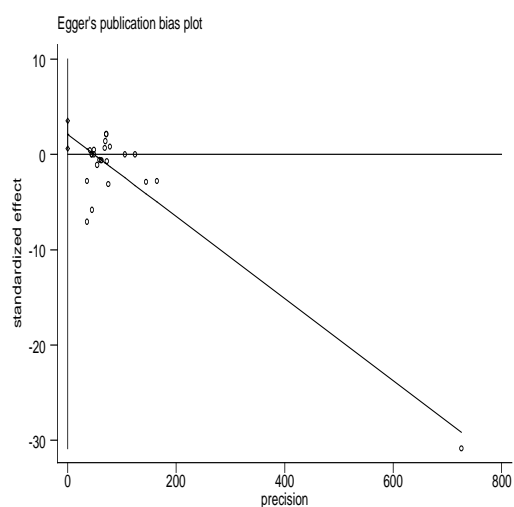

R

Supplement: Supplementary Figure 1 — Forest plots of overall effects. (A): Overall effect of TCDD and sperm motility (%); (B): Overall effect of TCDD and abnormal sperm (%); (C): Overall effect of TCDD and anogenital distance(mm); (D): Overall effect of TCDD and relative anogenital distance (%body length); (E): Overall effect of TCDD and seminal vesicle weight (g); (F): Overall effect of TCDD and prostate weight (g) [file DataSheet_1.zip › DATA sheet 1/Supplementary Figure 22.pdf]

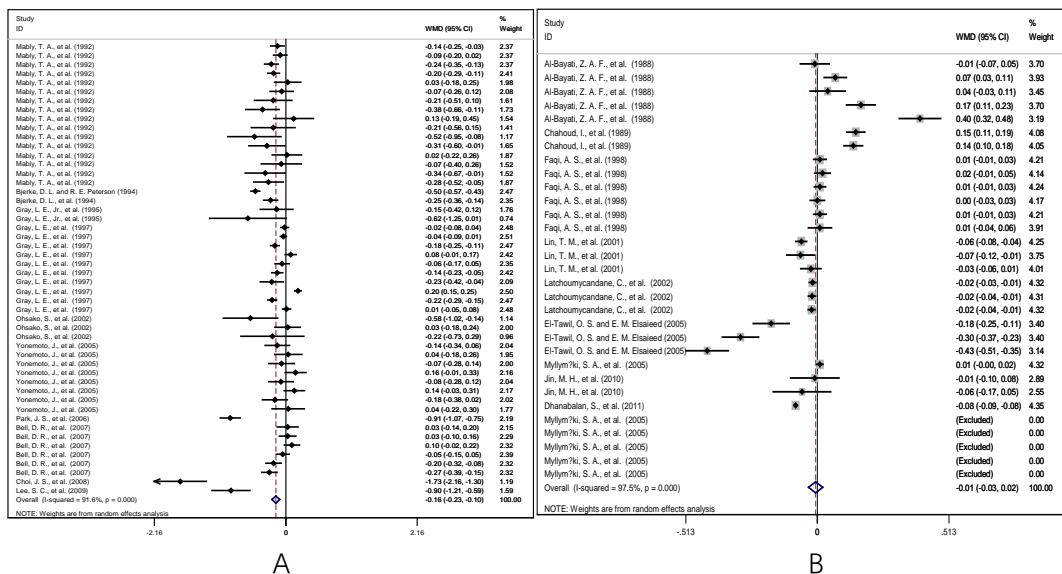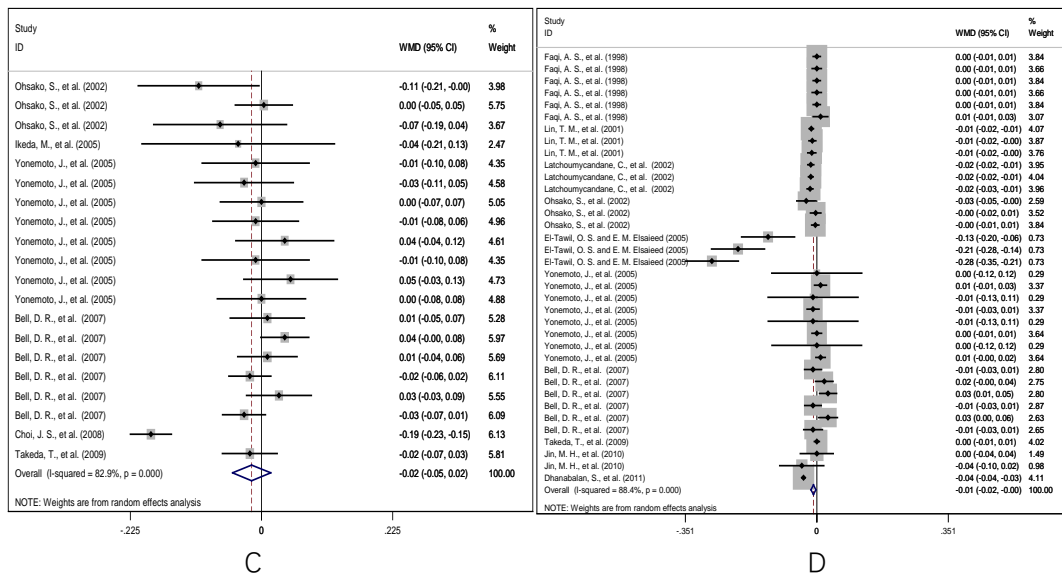

Supplement: Supplementary Figure 1 — Forest plots of overall effects. (A): Overall effect of TCDD and sperm motility (%); (B): Overall effect of TCDD and abnormal sperm (%); (C): Overall effect of TCDD and anogenital distance(mm); (D): Overall effect of TCDD and relative anogenital distance (%body length); (E): Overall effect of TCDD and seminal vesicle weight (g); (F): Overall effect of TCDD and prostate weight (g) [file DataSheet_1.zip › DATA sheet 1/Supplementary Figure 3.pdf]

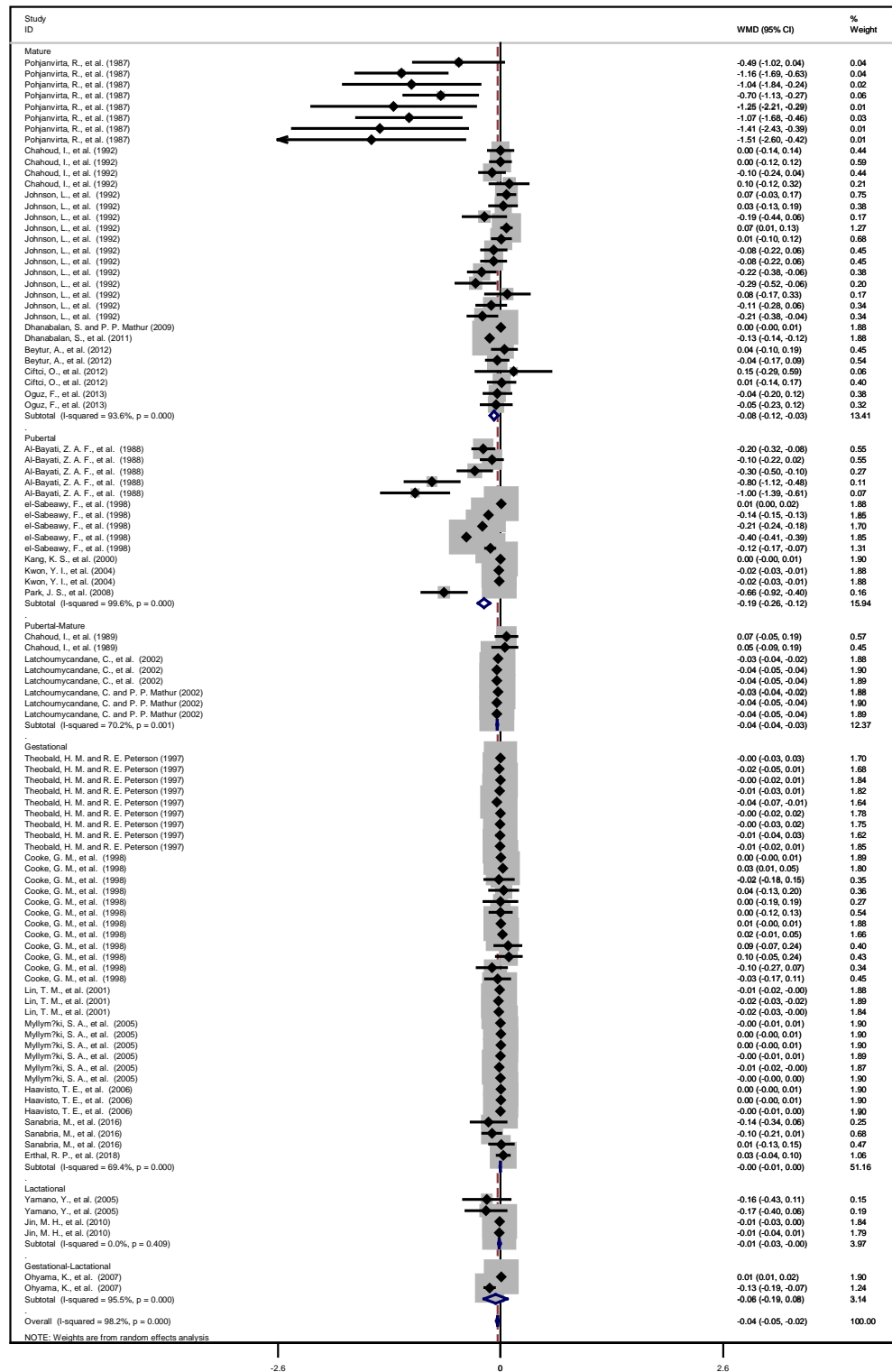

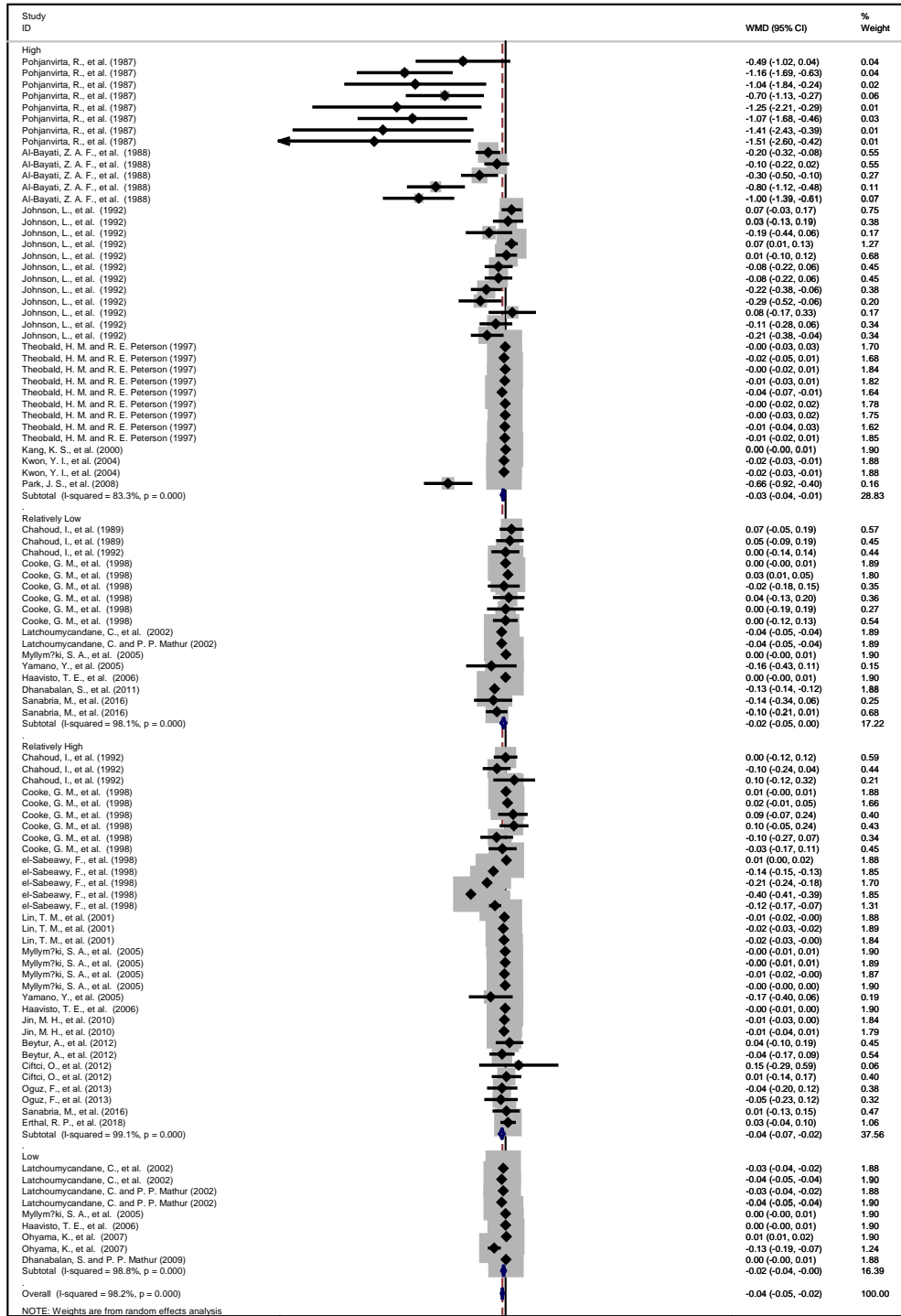

Supplement: Supplementary Figure 1 — Forest plots of overall effects. (A): Overall effect of TCDD and sperm motility (%); (B): Overall effect of TCDD and abnormal sperm (%); (C): Overall effect of TCDD and anogenital distance(mm); (D): Overall effect of TCDD and relative anogenital distance (%body length); (E): Overall effect of TCDD and seminal vesicle weight (g); (F): Overall effect of TCDD and prostate weight (g) [file DataSheet_1.zip › DATA sheet 1/Supplementary Figure 4.pdf]

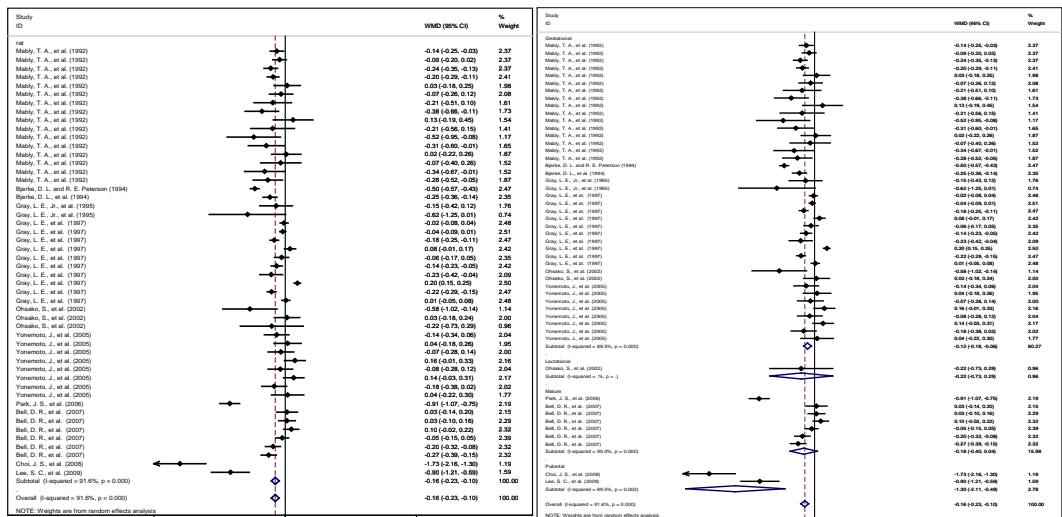

A

B

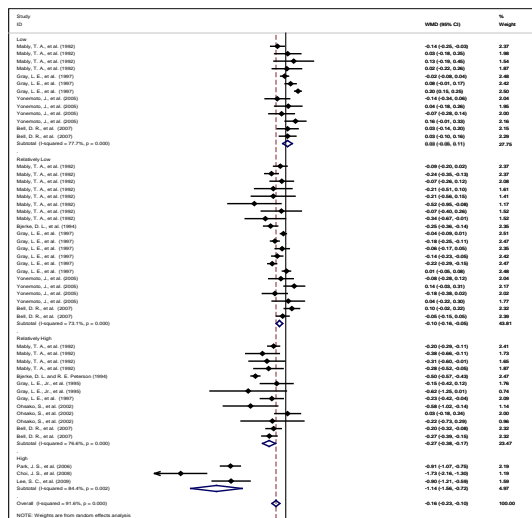

C

Supplement: Supplementary Figure 1 — Forest plots of overall effects. (A): Overall effect of TCDD and sperm motility (%); (B): Overall effect of TCDD and abnormal sperm (%); (C): Overall effect of TCDD and anogenital distance(mm); (D): Overall effect of TCDD and relative anogenital distance (%body length); (E): Overall effect of TCDD and seminal vesicle weight (g); (F): Overall effect of TCDD and prostate weight (g) [file DataSheet_1.zip › DATA sheet 1/Supplementary Figure 5.pdf]

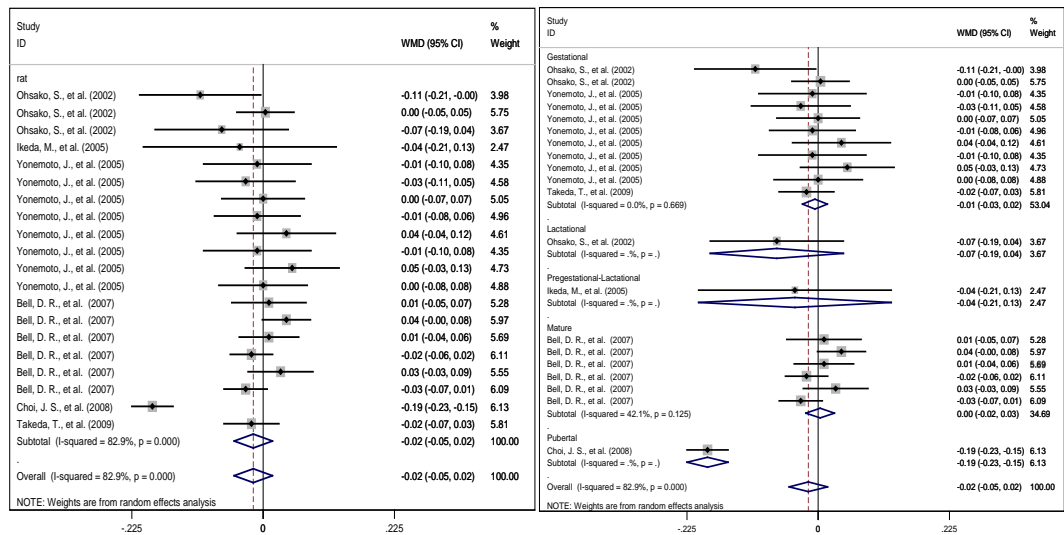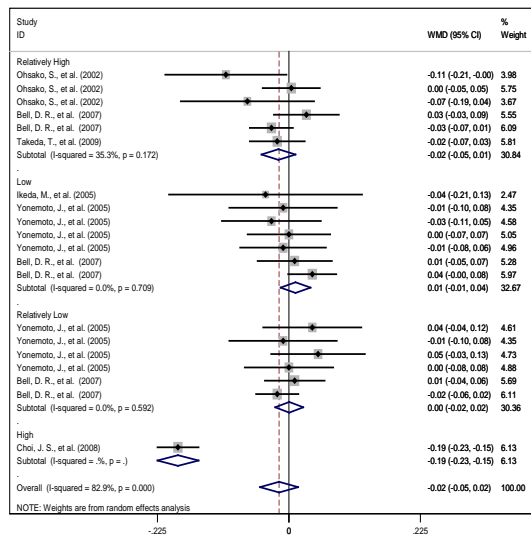

Supplement: Supplementary Figure 1 — Forest plots of overall effects. (A): Overall effect of TCDD and sperm motility (%); (B): Overall effect of TCDD and abnormal sperm (%); (C): Overall effect of TCDD and anogenital distance(mm); (D): Overall effect of TCDD and relative anogenital distance (%body length); (E): Overall effect of TCDD and seminal vesicle weight (g); (F): Overall effect of TCDD and prostate weight (g) [file DataSheet_1.zip › DATA sheet 1/Supplementary Figure 7.pdf]

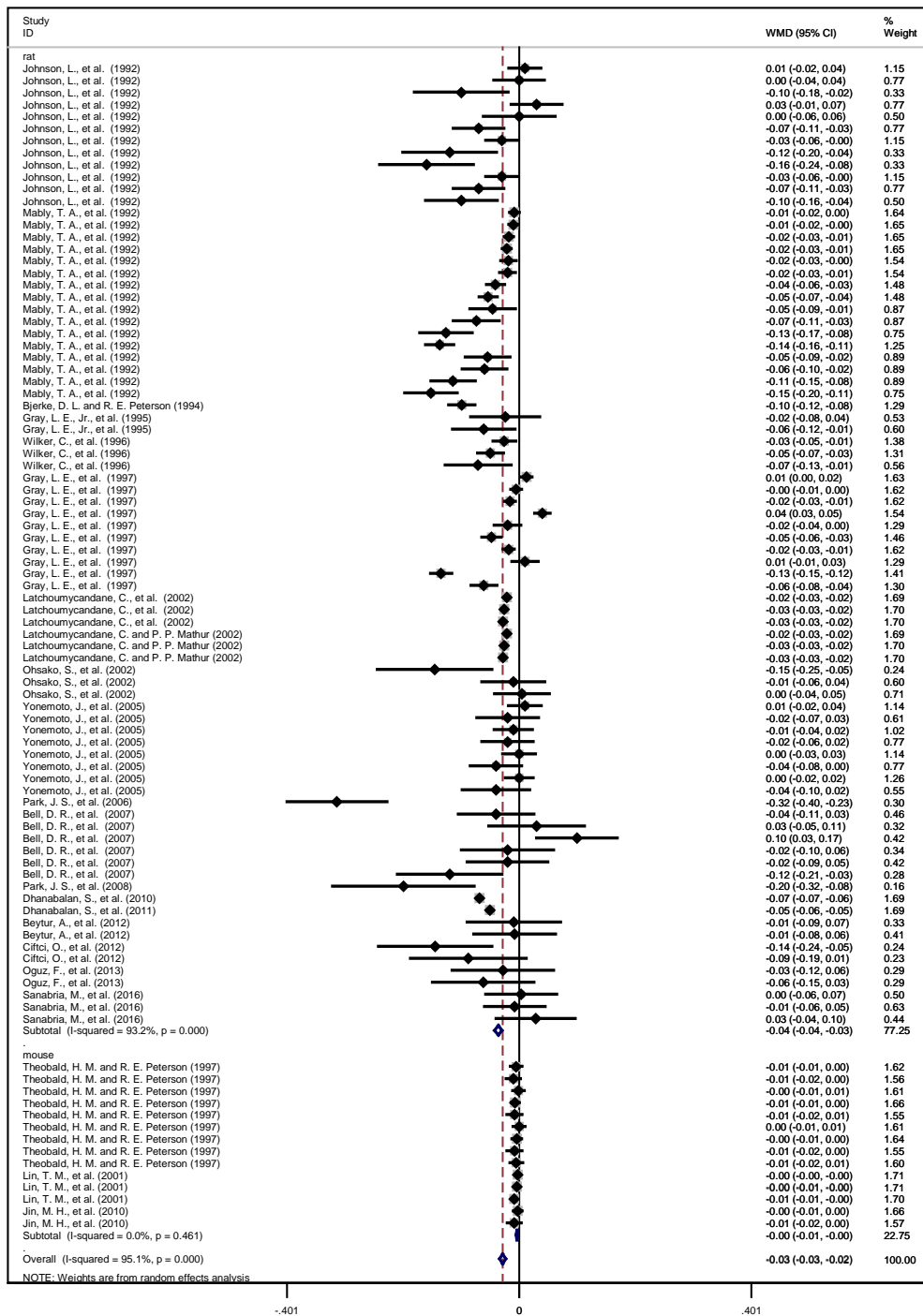

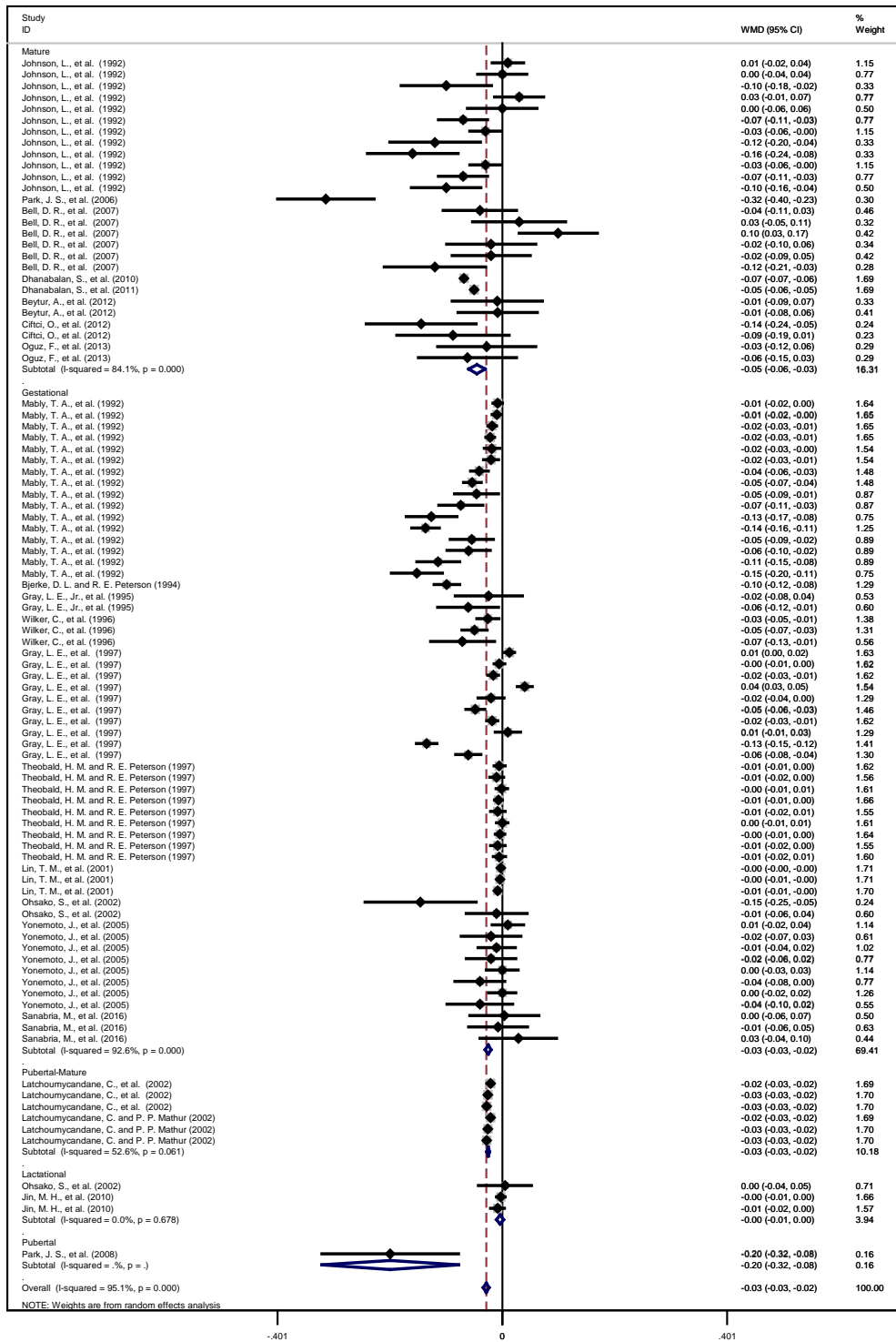

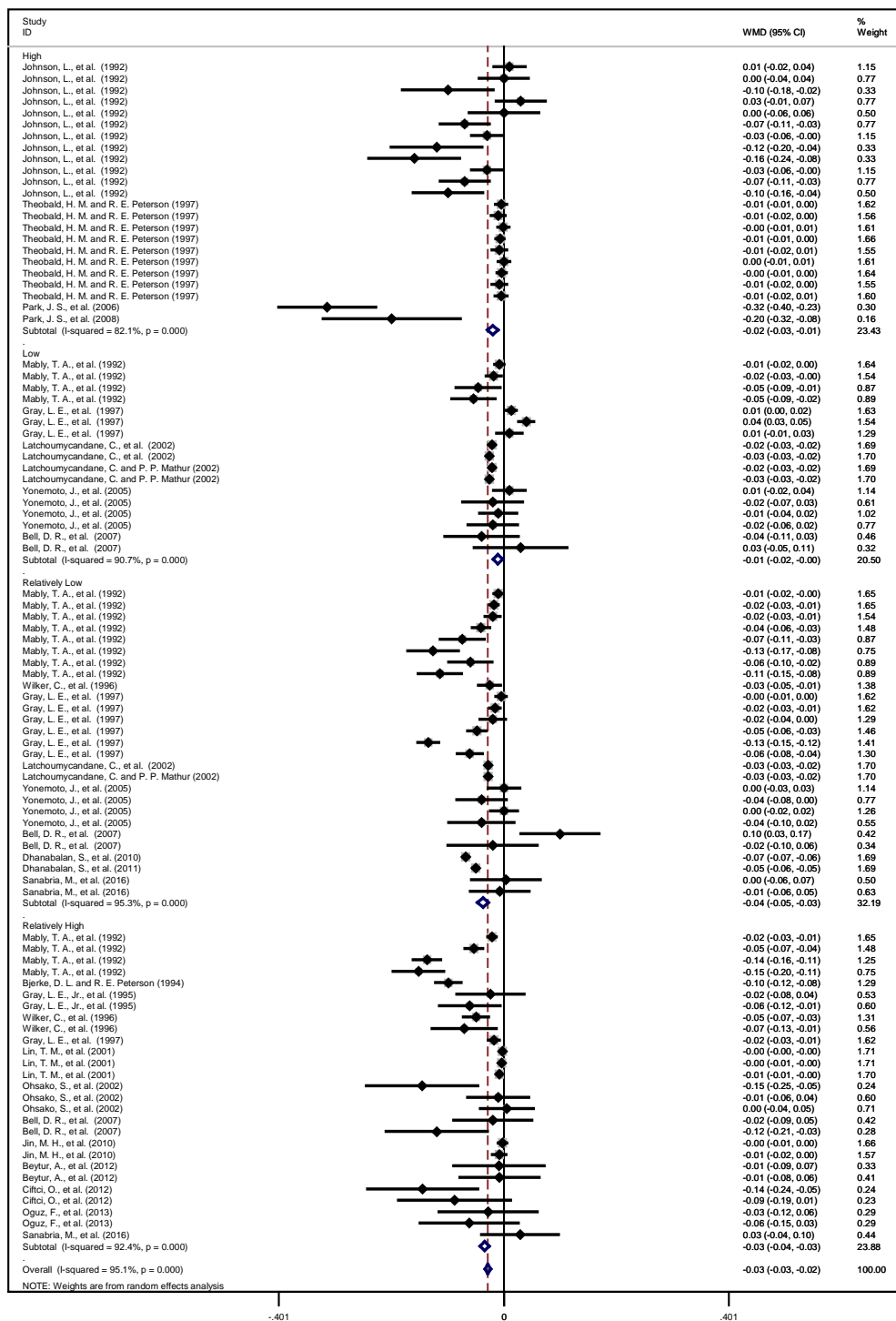

Supplement: Supplementary Figure 1 — Forest plots of overall effects. (A): Overall effect of TCDD and sperm motility (%); (B): Overall effect of TCDD and abnormal sperm (%); (C): Overall effect of TCDD and anogenital distance(mm); (D): Overall effect of TCDD and relative anogenital distance (%body length); (E): Overall effect of TCDD and seminal vesicle weight (g); (F): Overall effect of TCDD and prostate weight (g) [file DataSheet_1.zip › DATA sheet 1/Supplementary Figure 8.pdf]

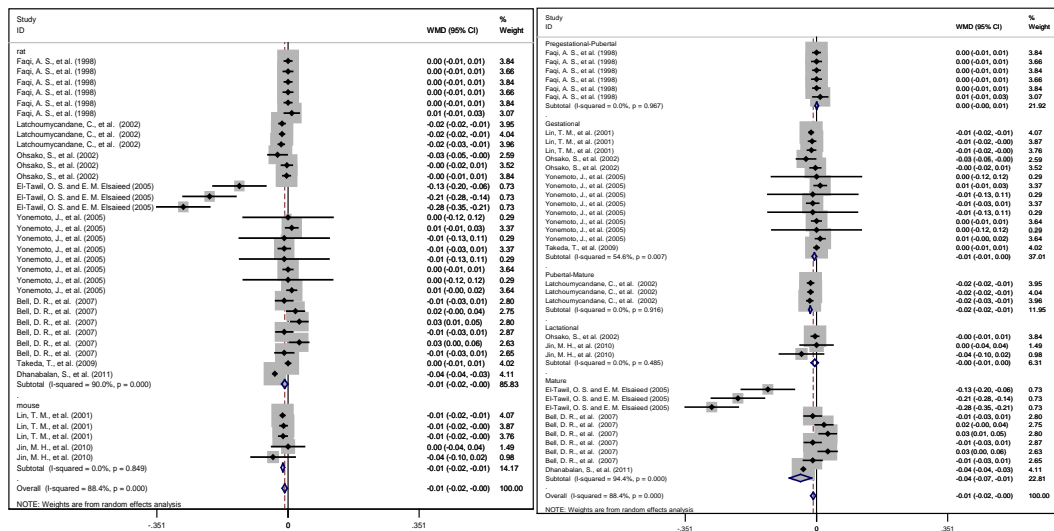

A

B

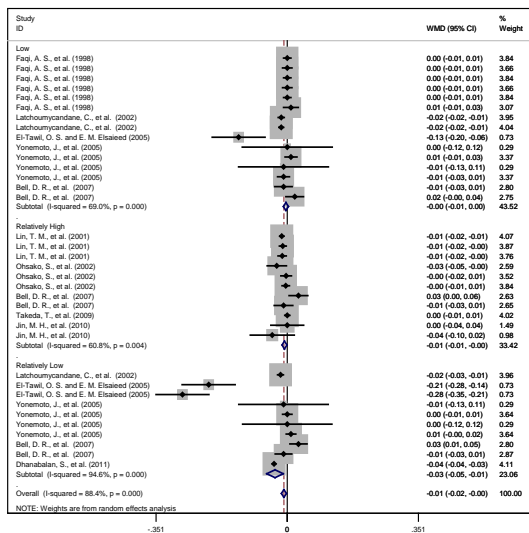

C

Supplement: Supplementary Figure 1 — Forest plots of overall effects. (A): Overall effect of TCDD and sperm motility (%); (B): Overall effect of TCDD and abnormal sperm (%); (C): Overall effect of TCDD and anogenital distance(mm); (D): Overall effect of TCDD and relative anogenital distance (%body length); (E): Overall effect of TCDD and seminal vesicle weight (g); (F): Overall effect of TCDD and prostate weight (g) [file DataSheet_1.zip › DATA sheet 1/Supplementary Figure 9.pdf]
