# Supplementary material for: Animal Toxicology Studies on the Male Reproductive Effects of 2,3,7,8-Tetrachlorodibenzo-p-Dioxin: Data Analysis and Health Effects Evaluation
Source: Front Endocrinol (Lausanne). 2021 Nov 3;12:696106. doi: 10.3389/fendo.2021.696106 (PMC8595279; doi:10.3389/fendo.2021.696106)
Supplement: Supplementary Table 0 — Topic statement and problem formulation. [file DataSheet_2.zip › DATA sheet 2/Supplementary Table 0.docx]

| **Titles** | **Details** |
| --- | --- |
| Topic statement | Is exposure to 2,3,7,8-tetrachlorodibenzo-p-dioxin (TCDD) associated with adverse rodent male reproductive system outcomes? |
| Population | Male rat or mouse species |
| Exposure | Exposure to TCDD (CAS Number: 1746-01-6) during pregestational, lactational, pubertal, or adult life stage by direct or indirect ways |
| Comparator | Male rat or mouse species exposed to different dosages of TCDD or vehicle only |
| Outcome | Male reproductive system-related parameters: testicular weight, prostate weight, serum testosterone, and sperm motility |
