# Supplementary material for: Animal Toxicology Studies on the Male Reproductive Effects of 2,3,7,8-Tetrachlorodibenzo-p-Dioxin: Data Analysis and Health Effects Evaluation
Source: Front Endocrinol (Lausanne). 2021 Nov 3;12:696106. doi: 10.3389/fendo.2021.696106 (PMC8595279; doi:10.3389/fendo.2021.696106)
Supplement: Supplementary Table 0 — Topic statement and problem formulation. [file DataSheet_2.zip › DATA sheet 2/Supplementary Table 1.docx]

Free terms used in strategy

| Free terms of chemical: | Dibenzodioxin* OR Dibenzo-p-dioxin* OR dioxin* OR Polychlorodibenzo-4-dioxin* OR Polychlorodibenzo* OR Polychlorodibenzo-p-dioxin* OR Tetrachlorodibenzodioxin* OR Tetrachlorodibenzo* OR Tetrachlorodibenzo-p-dioxin* OR TCDD OR PCDD |
| --- | --- |
| Free terms of male reproductive system: | Male OR Genital* OR Reproductive OR Sex OR sexual* OR Penis OR penile OR ejacula* OR erecti* OR prepuce OR foreskin OR testis OR testes OR testic* OR testosterone OR androgen* OR andrology* OR urogenit* OR sperm OR spermi* OR spermati* OR spermato* OR Leydig OR Sertoli OR cryptorchidism OR semen* OR semin* OR Prostate OR epididym* OR hypospadias* OR gonocyt* |
| Free terms of species | mouse or mice or rat or rats |

Database, Search Strategy and Outcomes:

| Database | Search Strategy | Outcomes |
| --- | --- | --- |
| Pubmed | Search (((rat or rats or mice or mouse)) AND ((((Dibenzodioxinx OR Dibenzo-p-dioxin* OR dioxin* OR Polychlorodibenzo-4-dioxin* OR Polychlorodibenzo* OR Polychlorodibenzo-p-dioxin* OR Tetrachlorodibenzodioxin* OR Tetrachlorodibenzo* OR Tetrachlorodibenzo-p-dioxin* OR TCDD OR PCDD))) OR "Polychlorinated Dibenzodioxins"[Mesh])) AND ((((Male OR Genital* OR Reproductive OR Sex OR sexual* OR Penis OR penile OR ejacula* OR erecti* OR prepuce OR foreskin OR testis OR testes OR testic* OR testosterone OR androgen* OR andrology* OR urogenit* OR sperm OR spermi* OR spermati* OR spermato* OR Leydig OR Sertoli OR cryptorchidism OR semen* OR semin* OR Prostate OR epididym* OR hypospadias* OR gonocyt*))) OR "Genitalia, Male"[Mesh]) | 2609 |
| Cochrane | Male OR Genital* OR Reproductive OR Sex OR sexual* OR Penis OR penile OR ejacula* OR erecti* OR prepuce OR foreskin OR testis OR testes OR testic* OR testosterone OR androgen* OR andrology* OR urogenit* OR sperm OR spermi* OR spermati* OR spermato* OR Leydig OR Sertoli OR cryptorchidism OR semen* OR semin* OR Prostate OR epididym* OR hypospadias* OR gonocyt* in All Text AND Dibenzodioxin* OR dioxin* OR Polychlorodibenzo* OR Tetrachlorodibenzodioxin* OR Tetrachlorodibenzo* OR TCDD OR PCDD in All Text AND rat or rats or mice or mouse in All Text • (Word variations have been searched) | 3 |
| Embase | (dibenzodioxin* OR 'dibenzo p dioxin*' OR dioxin* OR 'polychlorodibenzo 4 dioxin*' OR polychlorodibenzo* OR 'polychlorodibenzo p dioxin*' OR tetrachlorodibenzodioxin* OR tetrachlorodibenzo* OR 'tetrachlorodibenzo p dioxin*' OR tcdd OR pcdd) AND (male OR genital* OR reproductive OR sex OR sexual* OR penis OR penile OR ejacula* OR erecti* OR prepuce OR foreskin OR testis OR testes OR testic* OR testosterone OR androgen* OR andrology* OR urogenit* OR sperm OR spermi* OR spermati* OR spermato* OR leydig OR sertoli OR cryptorchidism OR semen* OR semin* OR prostate OR epididym* OR hypospadias* OR gonocyt*) AND (rat OR rats OR mice OR mouse) | 2284 |
