# Supplementary material for: Animal Toxicology Studies on the Male Reproductive Effects of 2,3,7,8-Tetrachlorodibenzo-p-Dioxin: Data Analysis and Health Effects Evaluation
Source: Front Endocrinol (Lausanne). 2021 Nov 3;12:696106. doi: 10.3389/fendo.2021.696106 (PMC8595279; doi:10.3389/fendo.2021.696106)
Supplement: Supplementary Table 0 — Topic statement and problem formulation. [file DataSheet_2.zip › DATA sheet 2/Supplementary Table 10.docx]

| Species | D+L pooled WMD | [95% Conf. Interval] | % Weight | I-squared** | p |
| --- | --- | --- | --- | --- | --- |
| Rat | -0.009 | (-0.017, -0.001) | 85.83 | 90.0% | 0.000 |
| Mouse | -0.014 | (-0.018, -0.009) | 14.17 | 0.0% | 0.849 |

A

| Exposure Windows | D+L pooled WMD | [95% Conf. Interval] | % Weight | I-squared** | p |
| --- | --- | --- | --- | --- | --- |
| Pregestational-Pubertal | 0.001 | (-0.004, 0.005) | 21.92 | 0.0% | 0.967 |
| Gestational | -0.006 | (-0.011, 0.000) | 37.01 | 54.6% | 0.007 |
| Pubertal-Mature | -0.017 | (-0.021, -0.012) | 11.95 | 0.0% | 0.916 |
| Lactational | -0.005 | (-0.014, 0.005) | 6.31 | 0.0% | 0.485 |
| Mature | -0.043 | (-0.073, -0.013) | 22.81 | 94.4% | 0.000 |

B

| Dosage Levels | D+L pooled WMD | [95% Conf. Interval] | % Weight | I-squared** | p |
| --- | --- | --- | --- | --- | --- |
| Low | -0.004 | (-0.01, 0.003) | 43.52 | 69.0% | 0.000 |
| Relatively High | -0.007 | (-0.014, -0.001) | 33.42 | 60.8% | 0.004 |
| Relatively Low | -0.033 | (-0.055, -0.011) | 23.06 | 94.6% | 0.000 |

C
