# Supplementary material for: Animal Toxicology Studies on the Male Reproductive Effects of 2,3,7,8-Tetrachlorodibenzo-p-Dioxin: Data Analysis and Health Effects Evaluation
Source: Front Endocrinol (Lausanne). 2021 Nov 3;12:696106. doi: 10.3389/fendo.2021.696106 (PMC8595279; doi:10.3389/fendo.2021.696106)
Supplement: Supplementary Table 0 — Topic statement and problem formulation. [file DataSheet_2.zip › DATA sheet 2/Supplementary Table 11.docx]

| Species | D+L pooled WMD | [95% Conf. Interval] | % Weight | I-squared** | p |
| --- | --- | --- | --- | --- | --- |
| Rat | -0.03 | (-0.038, -0.023) | 75.77 | 96.5% | 0.000 |
| Mouse | -0.005 | (-0.006, -0.004) | 24.23 | 0.0% | 0.701 |

A

| Exposure Windows | D+L pooled WMD | [95% Conf. Interval] | % Weight | I-squared** | p |
| --- | --- | --- | --- | --- | --- |
| Mature | -0.078 | (-0.096, -0.060) | 8.05 | 92.6% | 0.000 |
| Gestational | -0.019 | (-0.025, -0.013) | 72.72 | 94.9% | 0.000 |
| Pubertal | 0.01 | (0.006, 0.014) | 2.68 | / | / |
| Pubertal-Mature | -0.015 | (-0.016, -0.013) | 16.18 | 51.4% | 0.068 |
| Lactational | 0.000 | (-0.08, 0.08) | 0.37 | / | / |

B

| Dosage Levels | D+L pooled WMD | [95% Conf. Interval] | % Weight | I-squared** | p |
| --- | --- | --- | --- | --- | --- |
| High | -0.008 | (-0.013, -0.003) | 29.58 | 92.8% | 0.000 |
| Low | -0.003 | (-0.009, 0.003) | 23.96 | 89.3% | 0.000 |
| Relatively Low | -0.025 | (-0.033, -0.018) | 35.39 | 91.2% | 0.000 |
| Relatively High | -0.073 | (-0.139, -0.007) | 11.08 | 98.2% | 0.000 |

C
