# Supplementary material for: Animal Toxicology Studies on the Male Reproductive Effects of 2,3,7,8-Tetrachlorodibenzo-p-Dioxin: Data Analysis and Health Effects Evaluation
Source: Front Endocrinol (Lausanne). 2021 Nov 3;12:696106. doi: 10.3389/fendo.2021.696106 (PMC8595279; doi:10.3389/fendo.2021.696106)
Supplement: Supplementary Table 0 — Topic statement and problem formulation. [file DataSheet_2.zip › DATA sheet 2/Supplementary Table 12.docx]

| Species | D+L pooled WMD | [95% Conf. Interval] | % Weight | I-squared** | p |
| --- | --- | --- | --- | --- | --- |
| Rat | -0.001 | (-0.004, 0.002) | 100 | 65.4% | 0.000 |
| / | / | / | / | / | / |

A

| Exposure Windows | D+L pooled WMD | [95% Conf. Interval] | % Weight | I-squared** | p |
| --- | --- | --- | --- | --- | --- |
| Gestational | -0.001 | (-0.004, 0.002) | 98.53 | 70.7% | 0.000 |
| Mature | -0.018 | (-0.041, 0.006) | 1.47 | 52.4% | 0.032 |

B

| Dosage Levels | D+L pooled WMD | [95% Conf. Interval] | % Weight | I-squared** | p |
| --- | --- | --- | --- | --- | --- |
| Relatively Low | 0.001 | (-0.003, 0.004) | 60.99 | 0.0% | 0.661 |
| Relatively High | -0.004 | (-0.009, 0.000) | 38.54 | 76.3% | 0.000 |
| Low | -0.01 | (-0.004, 0.031) | 0.47 | 85.2% | 0.009 |

C
