# Supplementary material for: Animal Toxicology Studies on the Male Reproductive Effects of 2,3,7,8-Tetrachlorodibenzo-p-Dioxin: Data Analysis and Health Effects Evaluation
Source: Front Endocrinol (Lausanne). 2021 Nov 3;12:696106. doi: 10.3389/fendo.2021.696106 (PMC8595279; doi:10.3389/fendo.2021.696106)
Supplement: Supplementary Table 0 — Topic statement and problem formulation. [file DataSheet_2.zip › DATA sheet 2/Supplementary Table 13.docx]

| Species | D+L pooled WMD | [95% Conf. Interval] | % Weight | I-squared** | p |
| --- | --- | --- | --- | --- | --- |
| Rat | -0.012 | (-0.015, -0.009) | 75.67 | 34.8% | 0.078 |
| Mouse | 0.004 | (-0.003, 0.011) | 24.33 | 78.3% | 0.01 |

A

| Exposure Windows | D+L pooled WMD | [95% Conf. Interval] | % Weight | I-squared** | p |
| --- | --- | --- | --- | --- | --- |
| Pubertal-Mature | -0.011 | (-0.013, -0.008) | 26.98 | 35.9% | 0.21 |
| Gestational | -0.016 | (-0.023, -0.009) | 35.95 | 33.8% | 0.119 |
| Lactation | 0.003 | (-0.003, 0.009) | 28.09 | 68.0% | 0.025 |
| Mature | -0.013 | (-0.016, -0.009) | 8.98 | / | / |

B

| Dosage Levels | D+L pooled WMD | [95% Conf. Interval] | % Weight | I-squared** | p |
| --- | --- | --- | --- | --- | --- |
| Low | -0.007 | (-0.012, -0.002) | 38.03 | 70.3% | 0.003 |
| Relatively Low | -0.009 | (-0.017, -0.001) | 36.03 | 86.2% | 0.000 |
| Relatively High | -0.014 | (-0.028, -0.000) | 25.94 | 83.4% | 0.000 |

C
