# Supplementary material for: Animal Toxicology Studies on the Male Reproductive Effects of 2,3,7,8-Tetrachlorodibenzo-p-Dioxin: Data Analysis and Health Effects Evaluation
Source: Front Endocrinol (Lausanne). 2021 Nov 3;12:696106. doi: 10.3389/fendo.2021.696106 (PMC8595279; doi:10.3389/fendo.2021.696106)
Supplement: Supplementary Table 0 — Topic statement and problem formulation. [file DataSheet_2.zip › DATA sheet 2/Supplementary Table 14.docx]

| Species | D+L pooled WMD | [95% Conf. Interval] | % Weight | I-squared** | p |
| --- | --- | --- | --- | --- | --- |
| Rat | -0.041 | (-0.051, -0.032) | 100 | 94.8% | 0.000 |
| / | / | / | / | / | / |

A

| Exposure Windows | D+L pooled WMD | [95% Conf. Interval] | % Weight | I-squared** | p |
| --- | --- | --- | --- | --- | --- |
| Mature | -0.116 | (-0.162, -0.071) | 16.41 | 95.7% | 0.000 |
| Gestational | -0.022 | (-0.030, -0.014) | 55.90 | 85.5% | 0.000 |
| Pubertal-Mature | -0.037 | (-0.045, -0.030) | 27.69 | 87.6% | 0.000 |

B

| Dosage Levels | D+L pooled WMD | [95% Conf. Interval] | % Weight | I-squared** | p |
| --- | --- | --- | --- | --- | --- |
| High | -0.357 | (-0.532, -0.183) | 2.31 | 88.6% | 0.000 |
| Low | -0.021 | (-0.034, -0.008) | 29.11 | 88.3% | 0.000 |
| Relatively Low | -0.032 | (-0.047, -0.016) | 38.68 | 94.0% | 0.000 |
| Relatively High | -0.038 | (-0.050, -0.025) | 29.91 | 91.6% | 0.000 |

C
