# Supplementary material for: Animal Toxicology Studies on the Male Reproductive Effects of 2,3,7,8-Tetrachlorodibenzo-p-Dioxin: Data Analysis and Health Effects Evaluation
Source: Front Endocrinol (Lausanne). 2021 Nov 3;12:696106. doi: 10.3389/fendo.2021.696106 (PMC8595279; doi:10.3389/fendo.2021.696106)
Supplement: Supplementary Table 0 — Topic statement and problem formulation. [file DataSheet_2.zip › DATA sheet 2/Supplementary Table 15.docx]

| Species | D+L pooled WMD | [95% Conf. Interval] | % Weight | I-squared** | p |
| --- | --- | --- | --- | --- | --- |
| Rat | -0.510 | (-0.633, -0.386) | 96.47 | 92.8% | 0.000 |
| Mouse | -1.243 | (-1.798, -0.688) | 3.53 | 0.0% | 0.521 |

A

| Exposure Windows | D+L pooled WMD | [95% Conf. Interval] | % Weight | I-squared** | p |
| --- | --- | --- | --- | --- | --- |
| Gestational | -0.538 | (-0.666, -0.410) | 94.62 | 92.9% | 0.000 |
| Lactational | -1.079 | (-1.810, -0.349) | 2.19 | 0.0% | 0.591 |
| Pregestational-Lactational | -0.140 | (-0.304, 0.024) | 3.19 | / | / |

B

| Dosage Levels | D+L pooled WMD | [95% Conf. Interval] | % Weight | I-squared** | p |
| --- | --- | --- | --- | --- | --- |
| Relatively High | -0.608 | (-0.769, -0.446) | 64.89 | 94.3% | 0.000 |
| Low | -0.157 | (-0.293, -0.021) | 8.27 | 0.0% | 0.722 |
| Relatively Low | -0.485 | (-0.659, -0.31) | 26.84 | 64.7% | 0.002 |

C
