# Supplementary material for: Animal Toxicology Studies on the Male Reproductive Effects of 2,3,7,8-Tetrachlorodibenzo-p-Dioxin: Data Analysis and Health Effects Evaluation
Source: Front Endocrinol (Lausanne). 2021 Nov 3;12:696106. doi: 10.3389/fendo.2021.696106 (PMC8595279; doi:10.3389/fendo.2021.696106)
Supplement: Supplementary Table 0 — Topic statement and problem formulation. [file DataSheet_2.zip › DATA sheet 2/Supplementary Table 16.docx]

| Species | D+L pooled WMD | [95% Conf. Interval] | % Weight | I-squared** | p |
| --- | --- | --- | --- | --- | --- |
| Rat | -0.788 | (-1.125, -0.451) | 93.45 | 92.8% | 0.000 |
| Mouse | -0.979 | (-1.919, -0.039) | 6.55 | 0.0% | 0.427 |

A

| Exposure Windows | D+L pooled WMD | [95% Conf. Interval] | % Weight | I-squared** | p |
| --- | --- | --- | --- | --- | --- |
| Gestational | -0.809 | (-1.154, -0.463) | 90.89 | 92.5% | 0.000 |
| Lactational | -0.700 | (-1.344, -0.055) | 9.11 | 21.2% | 0.281 |

B

| Dosage Levels | D+L pooled WMD | [95% Conf. Interval] | % Weight | I-squared** | p |
| --- | --- | --- | --- | --- | --- |
| Relatively High | -0.836 | (-1.255, -0.418) | 67.66 | 93.7% | 0.000 |
| Low | -0.310 | (-0.823, 0.203) | 7.65 | 0.0% | 0.885 |
| Relatively Low | -0.858 | (-1.330, -0.385) | 24.36 | 72.4% | 0.003 |

C
