# Supplementary material for: Animal Toxicology Studies on the Male Reproductive Effects of 2,3,7,8-Tetrachlorodibenzo-p-Dioxin: Data Analysis and Health Effects Evaluation
Source: Front Endocrinol (Lausanne). 2021 Nov 3;12:696106. doi: 10.3389/fendo.2021.696106 (PMC8595279; doi:10.3389/fendo.2021.696106)
Supplement: Supplementary Table 0 — Topic statement and problem formulation. [file DataSheet_2.zip › DATA sheet 2/Supplementary Table 17.docx]

| Species | D+L pooled WMD | [95% Conf. Interval] | % Weight | I-squared** | p |
| --- | --- | --- | --- | --- | --- |
| Rat | -41.058 | (-51.468, -30.648) | 84.62 | 99.5% | 0.000 |
| Mouse | -5.218 | (-6.659, -3.777) | 15.38 | 28.6% | 0.200 |

A

| Exposure Windows | D+L pooled WMD | [95% Conf. Interval] | % Weight | I-squared** | p |
| --- | --- | --- | --- | --- | --- |
| Mature | -62.746 | (-137.088, 11.595) | 11.595 | 99.8% | 0.000 |
| Gestational | -25.584 | (-30.255, -20.914) | 59.24 | 96.7% | 0.000 |
| Pregestational-Pubertal | -45.126 | (-69.125, -21.127) | 8.45 | 67.6% | 0.009 |
| Pubertal | -83.959 | (-181.238, 13.320) | 3.2 | 96.1% | 0.000 |
| Lactational | -0.801 | (-4.528, 2.926) | 7.72 | 94.5% | 0.000 |

B

| Dosage Levels | D+L pooled WMD | [95% Conf. Interval] | % Weight | I-squared** | p |
| --- | --- | --- | --- | --- | --- |
| High | -11.891 | (-19.238, -4.544) | 17.54 | 86.2% | 0.000 |
| Low | -19.706 | (-28.226, -11.185) | 21.97 | 91.7% | 0.000 |
| Relatively Low | -60.904 | (-85.387, -36.421) | 30.63 | 99.8% | 0.000 |
| Relatively High | -20.932 | (-27.036, -14.828) | 29.86 | 96.8% | 0.000 |

C
