# Supplementary material for: Animal Toxicology Studies on the Male Reproductive Effects of 2,3,7,8-Tetrachlorodibenzo-p-Dioxin: Data Analysis and Health Effects Evaluation
Source: Front Endocrinol (Lausanne). 2021 Nov 3;12:696106. doi: 10.3389/fendo.2021.696106 (PMC8595279; doi:10.3389/fendo.2021.696106)
Supplement: Supplementary Table 0 — Topic statement and problem formulation. [file DataSheet_2.zip › DATA sheet 2/Supplementary Table 18.docx]

| Species | D+L pooled WMD | [95% Conf. Interval] | % Weight | I-squared** | p |
| --- | --- | --- | --- | --- | --- |
| Rat | -0.203 | (-0.256, -0.150) | 96.71 | 97.7% | 0.000 |
| Mouse | -0.171 | (-0.254, -0.150) | 3.29 | 0.0% | 0.987 |

A

| Exposure Windows | D+L pooled WMD | [95% Conf. Interval] | % Weight | I-squared** | p |
| --- | --- | --- | --- | --- | --- |
| Mature | -2.483 | (-5.558, 0.592) | 1.23 | 83.6% | 0.000 |
| Gestational | -3.180 | (-4.115, -2.245) | 5.21 | 92.6% | 0.000 |
| Pubertal | -3.153 | (-6.154, -0.152) | 0.83 | 96.3% | 0.000 |
| Pregestational-Pubertal | -14.578 | (-19.763, -9.393) | 0.15 | 92.9% | 0.000 |
| Pubertal-Mature | -0.008 | (-0.01, -0.006) | 92.52 | 0.0% | 0.456 |
| Lactational | -0.250 | (-2.222, 1.722) | 0.07 | / | / |

B

| Dosage Levels | D+L pooled WMD | [95% Conf. Interval] | % Weight | I-squared** | p |
| --- | --- | --- | --- | --- | --- |
| High | -0.244 | (-0.546, 0.059) | 2.92 | 0.0% | 0.791 |
| Low | -0.029 | (-0.073, 0.015) | 62.51 | 97.5% | 0.000 |
| Relatively Low | -3.758 | (-5.871, -1.644) | 33.1 | 98.7% | 0.000 |
| Relatively High | -4.467 | (-6.437, -2.496) | 1.47 | 93.6% | 0.000 |

C
