# Supplementary material for: Animal Toxicology Studies on the Male Reproductive Effects of 2,3,7,8-Tetrachlorodibenzo-p-Dioxin: Data Analysis and Health Effects Evaluation
Source: Front Endocrinol (Lausanne). 2021 Nov 3;12:696106. doi: 10.3389/fendo.2021.696106 (PMC8595279; doi:10.3389/fendo.2021.696106)
Supplement: Supplementary Table 0 — Topic statement and problem formulation. [file DataSheet_2.zip › DATA sheet 2/Supplementary Table 19.docx]

| Species | D+L pooled WMD | [95% Conf. Interval] | % Weight | I-squared** | p |
| --- | --- | --- | --- | --- | --- |
| Rat | -0.168 | (-0.267, -0.068) | 97.9 | 96.6% | 0.000 |
| Mouse | -0.326 | (-0.482, -0.168) | 2.1 | / | / |

A

| Exposure Windows | D+L pooled WMD | [95% Conf. Interval] | % Weight | I-squared** | p |
| --- | --- | --- | --- | --- | --- |
| Gestational | -0.062 | (-0.118, -0.005) | 78.8 | 79.0% | 0.000 |
| Pregestational-Pubertal | -0.030 | (-0.602, 0.542) | 6 | 73.3% | 0.002 |
| Pubertal | -1.510 | (-3.837, 0.817) | 3.99 | 99.5% | 0.000 |
| Mature | -0.434 | (-0.784, -0.084) | 11.21 | 99.4% | 0.000 |

B

| Dosage Levels | D+L pooled WMD | [95% Conf. Interval] | % Weight | I-squared** | p |
| --- | --- | --- | --- | --- | --- |
| Low | -0.023 | (-0.111, 0.065) | 25.74 | 66.5% | 0.000 |
| Relatively Low | -0.168 | (-0.370, 0.034) | 36.51 | 97.5% | 0.000 |
| Relatively High | -0.109 | (-0.199, -0.019) | 33.76 | 87.5% | 0.000 |
| High | -1.510 | (-3.837, 0.817) | 3.99 | 99.5% | 0.000 |

C
