# Supplementary material for: Animal Toxicology Studies on the Male Reproductive Effects of 2,3,7,8-Tetrachlorodibenzo-p-Dioxin: Data Analysis and Health Effects Evaluation
Source: Front Endocrinol (Lausanne). 2021 Nov 3;12:696106. doi: 10.3389/fendo.2021.696106 (PMC8595279; doi:10.3389/fendo.2021.696106)
Supplement: Supplementary Table 0 — Topic statement and problem formulation. [file DataSheet_2.zip › DATA sheet 2/Supplementary Table 2.docx]

| D+L pooled WMD | [95% Conf. Interval] | % Weight |
| --- | --- | --- |
| -7.365 | (-10.338, -4.391) | 100 |
| Heterogeneity chi-squared = 205.02 (d.f. = 22) p = 0.000 | | |
| I-squared (variation in WMD attributable to heterogeneity) = 89.3% | | |

A

| D+L pooled WMD | [95% Conf. Interval] | % Weight |
| --- | --- | --- |
| 3.142 | (1.632, 4.653) | 100 |
| Heterogeneity chi-squared = 352.73 (d.f. = 20) p = 0.000 | | |
| I-squared (variation in WMD attributable to heterogeneity) = 94.3% | | |

B

| D+L pooled WMD | [95% Conf. Interval] | % Weight |
| --- | --- | --- |
| -0.536 | (-0.659, -0.414) | 100 |
| Heterogeneity chi-squared = 516.42 (d.f. = 40) p = 0.000 | | |
| I-squared (variation in WMD attributable to heterogeneity) = 92.3% | | |

C

| D+L pooled WMD | [95% Conf. Interval] | % Weight |
| --- | --- | --- |
| -0.806 | (-1.130, -0.482) | 100 |
| Heterogeneity chi-squared = 309.36 (d.f. = 26) p = 0.000 | | |
| I-squared (variation in WMD attributable to heterogeneity) = 91.6% | | |

D

| D+L pooled WMD | [95% Conf. Interval] | % Weight |
| --- | --- | --- |
| -0.041 | (-0.051, -0.032) | 100 |
| Heterogeneity chi-squared = 1076.68 (d.f. = 56) p = 0.000 | | |
| I-squared (variation in WMD attributable to heterogeneity) = 94.8% | | |

E

| D+L pooled WMD | [95% Conf. Interval] | % Weight |
| --- | --- | --- |
| -0.001 | (-0.004, 0.002) | 100 |
| Heterogeneity chi-squared = 66.49 (d.f. = 23) p = 0.000 | | |
| I-squared (variation in WMD attributable to heterogeneity) = 65.4% | | |

F
