# Supplementary material for: Animal Toxicology Studies on the Male Reproductive Effects of 2,3,7,8-Tetrachlorodibenzo-p-Dioxin: Data Analysis and Health Effects Evaluation
Source: Front Endocrinol (Lausanne). 2021 Nov 3;12:696106. doi: 10.3389/fendo.2021.696106 (PMC8595279; doi:10.3389/fendo.2021.696106)
Supplement: Supplementary Table 0 — Topic statement and problem formulation. [file DataSheet_2.zip › DATA sheet 2/Supplementary Table 20.docx]

| Species | D+L pooled WMD | [95% Conf. Interval] | % Weight | I-squared** | p |
| --- | --- | --- | --- | --- | --- |
| Rat | -7.365 | (-10.338, -4.391) | 100 | 89.3% | 0.000 |
| Mouse | / | / | / | / | / |

A

| Exposure Windows | D+L pooled WMD | [95% Conf. Interval] | % Weight | I-squared** | p |
| --- | --- | --- | --- | --- | --- |
| Gestational | -3.044 | (-5.672, -0.416) | 29.70 | 0.0% | 0.650 |
| Mature | -9.147 | (-12.752, -5.542) | 70.30 | 92.0% | 0.000 |

B

| Dosage Levels | D+L pooled WMD | [95% Conf. Interval] | % Weight | I-squared** | p |
| --- | --- | --- | --- | --- | --- |
| Low | -3.104 | (-7.874, 1.665) | 22.26 | 70.6% | 0.009 |
| Relatively Low | -6.873 | (-10.282, -3.464) | 47.93 | 86.4% | 0.000 |
| Relatively High | -12.043 | (-21.960, -2.127) | 29.81 | 93.5% | 0.000 |

C
