# Supplementary material for: Animal Toxicology Studies on the Male Reproductive Effects of 2,3,7,8-Tetrachlorodibenzo-p-Dioxin: Data Analysis and Health Effects Evaluation
Source: Front Endocrinol (Lausanne). 2021 Nov 3;12:696106. doi: 10.3389/fendo.2021.696106 (PMC8595279; doi:10.3389/fendo.2021.696106)
Supplement: Supplementary Table 0 — Topic statement and problem formulation. [file DataSheet_2.zip › DATA sheet 2/Supplementary Table 21.docx]

| Species | D+L pooled WMD | [95% Conf. Interval] | % Weight | I-squared** | p |
| --- | --- | --- | --- | --- | --- |
| Rat | 3.142 | (1.632, 4.653) | 100 | 94.3% | 0.000 |
| Mouse | / | / | / | / | / |

A

| Exposure Windows | D+L pooled WMD | [95% Conf. Interval] | % Weight | I-squared** | p |
| --- | --- | --- | --- | --- | --- |
| Gestational | 0.667 | (-0.170, 1.505) | 35.22 | 0.0% | 0.761 |
| Mature | 4.427 | (2.545, 6.308) | 64.78 | 95.8% | 0.000 |

B

| Dosage Levels | D+L pooled WMD | [95% Conf. Interval] | % Weight | I-squared** | p |
| --- | --- | --- | --- | --- | --- |
| Low | 1.825 | (-0.008, 3.659) | 24.80 | 87.6% | 0.000 |
| Relatively Low | 3.748 | (0.975, 6.522) | 42.32 | 96.0% | 0.000 |
| Relatively High | 3.393 | (0.558, 6.229) | 32.87 | 92.1% | 0.000 |

C
