# Supplementary material for: Animal Toxicology Studies on the Male Reproductive Effects of 2,3,7,8-Tetrachlorodibenzo-p-Dioxin: Data Analysis and Health Effects Evaluation
Source: Front Endocrinol (Lausanne). 2021 Nov 3;12:696106. doi: 10.3389/fendo.2021.696106 (PMC8595279; doi:10.3389/fendo.2021.696106)
Supplement: Supplementary Table 0 — Topic statement and problem formulation. [file DataSheet_2.zip › DATA sheet 2/Supplementary Table 22.docx]

| Species | D+L pooled WMD | [95% Conf. Interval] | % Weight | I-squared** | p |
| --- | --- | --- | --- | --- | --- |
| Rat | -0.016 | (-0.029, -0.002) | 100 | 90.2% | 0.000 |
| Mouse | / | / | / | / | / |

A

| Exposure Windows | D+L pooled WMD | [95% Conf. Interval] | % Weight | I-squared** | p |
| --- | --- | --- | --- | --- | --- |
| Pregestational-Pubertal | 0.000 | (-0.012, 0.012) | 10.04 | 0.0% | 1.000 |
| Pubertal-Mature | -0.022 | (-0.033, -0.011) | 14.95 | 31.1% | 0.234 |
| Mature | -0.033 | (-0.063, -0.004) | 41.09 | 93.4% | 0.000 |
| Gestational | 0.000 | (-0.012, 0.013) | 33.92 | 0.0% | 0.839 |

B

| Dosage Levels | D+L pooled WMD | [95% Conf. Interval] | % Weight | I-squared** | p |
| --- | --- | --- | --- | --- | --- |
| Low | -0.002 | (-0.014, 0.010) | 47 | 63.1% | 0.002 |
| Relatively Low | -0.038 | (-0.064, -0.012) | 40.14 | 90.0% | 0.000 |
| Relatively High | -0.001 | (-0.018, 0.016) | 12.87 | 0.0% | 0.516 |

C
