# Supplementary material for: Animal Toxicology Studies on the Male Reproductive Effects of 2,3,7,8-Tetrachlorodibenzo-p-Dioxin: Data Analysis and Health Effects Evaluation
Source: Front Endocrinol (Lausanne). 2021 Nov 3;12:696106. doi: 10.3389/fendo.2021.696106 (PMC8595279; doi:10.3389/fendo.2021.696106)
Supplement: Supplementary Table 0 — Topic statement and problem formulation. [file DataSheet_2.zip › DATA sheet 2/Supplementary Table 23.docx]

|  | Coef. | t | [95% Conf. Interval] |
| --- | --- | --- | --- |
| Bias | -1.880505 | -2.00 | (-3.746683, -0.143281) |

A

| D+L pooled WMD | [95% Conf. Interval] | % Weight |
| --- | --- | --- |
| -0.035 | (--0.046, -0.024) | 100 |
| Heterogeneity chi-squared = 5457.79 (d.f. = 95) p = 0.000 | | |
| I-squared (variation in WMD attributable to heterogeneity) = 98.3% | | |

B

|  | Coef. | t | [95% Conf. Interval] |
| --- | --- | --- | --- |
| Bias | -1.867368 | -1.96 | (-3.758611, 0.023876) |

C

|  | Coef. | t | [95% Conf. Interval] |
| --- | --- | --- | --- |
| Bias | -1.800707 | -2.07 | (-3.551304, -0.0501108) |

D

| D+L pooled WMD | [95% Conf. Interval] | % Weight |
| --- | --- | --- |
| -0.159 | (-0.223, -0.095) | 100 |
| Heterogeneity chi-squared = 579.49 (d.f. = 48) p = 0.000 | | |
| I-squared (variation in WMD attributable to heterogeneity) = 91.7% | | |

E

|  | Coef. | t | [95% Conf. Interval] |
| --- | --- | --- | --- |
| Bias | -1.792854 | -1.99 | (-3.607077, 0.0213695) |

F

|  | Coef. | t | [95% Conf. Interval] |
| --- | --- | --- | --- |
| Bias | 4.018609 | 2.45 | (0.6347726, 7.402445) |

G

| D+L pooled WMD | [95% Conf. Interval] | % Weight |
| --- | --- | --- |
| 0.007 | (-0.024, 0.037) | 100.00 |
| Heterogeneity chi-squared = 625.05 (d.f. = 13) p = 0.000 | | |
| I-squared (variation in WMD attributable to heterogeneity) = 97.9% | | |

H

|  | Coef. | t | [95% Conf. Interval] |
| --- | --- | --- | --- |
| Bias | 9.704451 | 4.95 | (5.435688, 13.97321) |

I

|  | Coef. | t | [95% Conf. Interval] |
| --- | --- | --- | --- |
| Bias | 1.591881 | 1.10 | (-1.454689, 4.638452) |

J

|  | Coef. | t | [95% Conf. Interval] |
| --- | --- | --- | --- |
| Bias | -1.576558 | -2.71 | (-2.734006, -0.4191103) |

K

| D+L pooled WMD | [95% Conf. Interval] | % Weight |
| --- | --- | --- |
| -0.024 | (-0.030, -0.019) | 100 |
| Heterogeneity chi-squared = 1707.86 (d.f. = 61) p = 0.000 | | |
| I-squared (variation in WMD attributable to heterogeneity) = 96.4% | | |

L

|  | Coef. | t | [95% Conf. Interval] |
| --- | --- | --- | --- |
| Bias | -1.834178 | -1.91 | (-3.759479, 0.0911233) |

M

|  | Coef. | t | [95% Conf. Interval] |
| --- | --- | --- | --- |
| Bias | 0.5865215 | 0.7 | (-1.114021, 2.287064) |

N

|  | Coef. | t | [95% Conf. Interval] |
| --- | --- | --- | --- |
| Bias | -1.46355 | -1.66 | (-3.222761, 0.2956613) |

O

|  | Coef. | t | [95% Conf. Interval] |
| --- | --- | --- | --- |
| Bias | -0.7722261 | -2.07 | (-1.55911, 0.0146582) |

P

|  | Coef. | t | [95% Conf. Interval] |
| --- | --- | --- | --- |
| Bias | -0.4853741 | -0.6 | (-2.184644, 1.213896) |

Q
