# Supplementary material for: Animal Toxicology Studies on the Male Reproductive Effects of 2,3,7,8-Tetrachlorodibenzo-p-Dioxin: Data Analysis and Health Effects Evaluation
Source: Front Endocrinol (Lausanne). 2021 Nov 3;12:696106. doi: 10.3389/fendo.2021.696106 (PMC8595279; doi:10.3389/fendo.2021.696106)
Supplement: Supplementary Table 0 — Topic statement and problem formulation. [file DataSheet_2.zip › DATA sheet 2/Supplementary Table 24.docx]

|  | Coef. | t | [95% Conf. Interval] |
| --- | --- | --- | --- |
| Bias | -2.272364 | -3.63 | (-3.528329, -1.016399) |

A

| D+L pooled WMD | [95% Conf. Interval] | % Weight |
| --- | --- | --- |
| -0.036 | (-0.045, -0.027) | 100 |
| Heterogeneity chi-squared = 904.92 (d.f. = 27) p = 0.000 | | |
| I-squared (variation in WMD attributable to heterogeneity) = 97.0% | | |

B

|  | Coef. | t | [95% Conf. Interval] |
| --- | --- | --- | --- |
| Bias | -4.540063 | -3.36 | (-7.317793, -1.762333) |

C

|  | Coef. | t | [95% Conf. Interval] |
| --- | --- | --- | --- |
| Bias | -2.662488 | -4.39 | (-3.889973, -1.435003) |

D

| D+L pooled WMD | [95% Conf. Interval] | % Weight |
| --- | --- | --- |
| -0.446 | (-0.604, -0.288) | 100 |
| Heterogeneity chi-squared = 408.19 (d.f. = 18) p = 0.000 | | |
| I-squared (variation in WMD attributable to heterogeneity) = 95.6% | | |

E

|  | Coef. | t | [95% Conf. Interval] |
| --- | --- | --- | --- |
| Bias | -3.585472 | -2.64 | (-6.446312, -0.7246332) |

F

|  | Coef. | t | [95% Conf. Interval] |
| --- | --- | --- | --- |
| Bias | -1.347966 | -1.19 | (-3.677457, 0.9815253) |

G

|  | Coef. | t | [95% Conf. Interval] |
| --- | --- | --- | --- |
| Bias | -4.637481 | -2.14 | (-8.963868, -0.3110935) |

H

| D+L pooled WMD | [95% Conf. Interval] | % Weight |
| --- | --- | --- |
| -30.752 | (-39.390, -22.115) | 100 |
| Heterogeneity chi-squared = 11455.29 (d.f. = 45) p = 0.000 | | |
| I-squared (variation in WMD attributable to heterogeneity) = 99.6% | | |

I

|  | Coef. | t | [95% Conf. Interval] |
| --- | --- | --- | --- |
| Bias | -6.298971 | -1.99 | (-12.6752, 0.0762611) |

J

|  | Coef. | t | [95% Conf. Interval] |
| --- | --- | --- | --- |
| Bias | -3.809927 | -5.53 | (-5.192483, -2.42737) |

K

| D+L pooled WMD | [95% Conf. Interval] | % Weight |
| --- | --- | --- |
| -0.111 | (-0.154, -0.068) | 100 |
| Heterogeneity chi-squared = 1298.70 (d.f. = 26) p = 0.000 | | |
| I-squared (variation in WMD attributable to heterogeneity) = 98.0% | | |

L

|  | Coef. | t | [95% Conf. Interval] |
| --- | --- | --- | --- |
| Bias | -4.023638 | -3.26 | (-6.569367, -1.477908) |

M

|  | Coef. | t | [95% Conf. Interval] |
| --- | --- | --- | --- |
| Bias | 0.6946631 | -0.71 | (-1.263714, 2.65304) |

N

|  | Coef. | t | [95% Conf. Interval] |
| --- | --- | --- | --- |
| Bias | 1.541941 | 1.55 | (-0.5267192, 3.6106) |

O

|  | Coef. | t | [95% Conf. Interval] |
| --- | --- | --- | --- |
| Bias | -2.39686 | -1.11 | (-6.923891, 2.13017) |

P

|  | Coef. | t | [95% Conf. Interval] |
| --- | --- | --- | --- |
| Bias | -2.075963 | 2.99 | (0.6353882, 3.516539) |

Q

| D+L pooled WMD | [95% Conf. Interval] | % Weight |
| --- | --- | --- |
| -0.004 | (-0.021, 0.013) | 100 |
| Heterogeneity chi-squared = 156.69 (d.f. = 11) p = 0.000 | | |
| I-squared (variation in WMD attributable to heterogeneity) = 93.0% | | |

R

|  | Coef. | t | [95% Conf. Interval] |
| --- | --- | --- | --- |
| Bias | 4.313072 | 7.32 | (2.999851, 5.626292) |

S
