# Supplementary material for: Animal Toxicology Studies on the Male Reproductive Effects of 2,3,7,8-Tetrachlorodibenzo-p-Dioxin: Data Analysis and Health Effects Evaluation
Source: Front Endocrinol (Lausanne). 2021 Nov 3;12:696106. doi: 10.3389/fendo.2021.696106 (PMC8595279; doi:10.3389/fendo.2021.696106)
Supplement: Supplementary Table 0 — Topic statement and problem formulation. [file DataSheet_2.zip › DATA sheet 2/Supplementary Table 3.docx]

| D+L pooled WMD | [95% Conf. Interval] | % Weight |
| --- | --- | --- |
| -0.009 | (-0.013, -0.005) | 100 |
| Heterogeneity chi-squared = 105.13 (d.f. = 19) p = 0.000 | | |
| I-squared (variation in WMD attributable to heterogeneity) = 81.9% | | |

A

| D+L pooled WMD | [95% Conf. Interval] | % Weight |
| --- | --- | --- |
| -0.016 | (-0.029, -0.002) | 100 |
| Heterogeneity chi-squared = 235.04 (d.f. = 23) p = 0.000 | | |
| I-squared (variation in WMD attributable to heterogeneity) = 90.2% | | |

B

| D+L pooled WMD | [95% Conf. Interval] | % Weight |
| --- | --- | --- |
| -0.035 | (-0.046, -0.025) | 100 |
| Heterogeneity chi-squared = 5464.98 (d.f. = 96) p = 0.000 | | |
| I-squared (variation in WMD attributable to heterogeneity) = 98.2% | | |

C

| D+L pooled WMD | [95% Conf. Interval] | % Weight |
| --- | --- | --- |
| -0.029 | (-0.034, -0.023) | 100 |
| Heterogeneity chi-squared = 1894.23 (d.f. = 93) p = 0.000 | | |
| I-squared (variation in WMD attributable to heterogeneity) = 95.1% | | |

D
