# Supplementary material for: Animal Toxicology Studies on the Male Reproductive Effects of 2,3,7,8-Tetrachlorodibenzo-p-Dioxin: Data Analysis and Health Effects Evaluation
Source: Front Endocrinol (Lausanne). 2021 Nov 3;12:696106. doi: 10.3389/fendo.2021.696106 (PMC8595279; doi:10.3389/fendo.2021.696106)
Supplement: Supplementary Table 0 — Topic statement and problem formulation. [file DataSheet_2.zip › DATA sheet 2/Supplementary Table 4.docx]

| D+L pooled WMD | [95% Conf. Interval] | % Weight |
| --- | --- | --- |
| -0.162 | (-0.226, -0.098) | 100 |
| Heterogeneity chi-squared = 582.19 (d.f. = 49) p = 0.000 | | |
| I-squared (variation in WMD attributable to heterogeneity) = 91.6% | | |

A

| D+L pooled WMD | [95% Conf. Interval] | % Weight |
| --- | --- | --- |
| -0.007 | (-0.035, 0.021) | 100 |
| Heterogeneity chi-squared = 982.96 (d.f. = 25) p = 0.000 | | |
| I-squared (variation in WMD attributable to heterogeneity) = 97.5% | | |

B

| D+L pooled WMD | [95% Conf. Interval] | % Weight |
| --- | --- | --- |
| -0.017 | (-0.05, 0.016) | 100 |
| Heterogeneity chi-squared = 111.35 (d.f. = 19) p = 0.000 | | |
| I-squared (variation in WMD attributable to heterogeneity) = 82.9% | | |

C

| D+L pooled WMD | [95% Conf. Interval] | % Weight |
| --- | --- | --- |
| -0.009 | （-0.016, -0.002） | 100 |
| Heterogeneity chi-squared = 302.13 (d.f. = 35) p = 0.000 | | |
| I-squared (variation in WMD attributable to heterogeneity) = 88.4% | | |

D
