# Supplementary material for: Animal Toxicology Studies on the Male Reproductive Effects of 2,3,7,8-Tetrachlorodibenzo-p-Dioxin: Data Analysis and Health Effects Evaluation
Source: Front Endocrinol (Lausanne). 2021 Nov 3;12:696106. doi: 10.3389/fendo.2021.696106 (PMC8595279; doi:10.3389/fendo.2021.696106)
Supplement: Supplementary Table 0 — Topic statement and problem formulation. [file DataSheet_2.zip › DATA sheet 2/Supplementary Table 5.docx]

| Species | D+L pooled WMD | [95% Conf. Interval] | % Weight | I-squared** | p |
| --- | --- | --- | --- | --- | --- |
| Rat | -0.045 | (-0.029, -0.032) | 71.34 | 98.5% | 0.000 |
| Mouse | -0.035 | (-0.046, -0.025) | 28.66 | 18.2% | 0.246 |

A

| Exposure Windows | D+L pooled WMD | [95% Conf. Interval] | % Weight | I-squared** | p |
| --- | --- | --- | --- | --- | --- |
| Mature | -0.075 | (-0.123, -0.027) | 13.41 | 93.6% | 0.000 |
| Pubertal | -0.192 | (-0.263, -0.122) | 15.94 | 99.6% | 0.000 |
| Pubertal-Mature | -0.038 | (-0.043, -0.033) | 12.37 | 70.2% | 0.001 |
| Gestational | -0.003 | (-0.006, 0.001) | 51.16 | 69.4% | 0.000 |
| Lactational | -0.014 | (-0.026, -0.002) | 3.97 | 0.0% | 0.409 |
| Gestational-Lactational | -0.056 | (-0.192, 0.081) | 3.14 | 95.5% | 0.000 |

B

| Dosage Levels | D+L pooled WMD | [95% Conf. Interval] | % Weight | I-squared** | p |
| --- | --- | --- | --- | --- | --- |
| High | -0.027 | (-0.043, -0.012) | 28.83 | 83.3% | 0.000 |
| Relatively Low | -0.022 | (-0.048, 0.003) | 17.22 | 98.1% | 0.000 |
| Relatively High | -0.040 | (-0.065, -0.015) | 37.56 | 99.1% | 0.000 |
| Low | -0.021 | (-0.038, -0.004) | 16.39 | 98.8% | 0.000 |

C
