# Supplementary material for: Animal Toxicology Studies on the Male Reproductive Effects of 2,3,7,8-Tetrachlorodibenzo-p-Dioxin: Data Analysis and Health Effects Evaluation
Source: Front Endocrinol (Lausanne). 2021 Nov 3;12:696106. doi: 10.3389/fendo.2021.696106 (PMC8595279; doi:10.3389/fendo.2021.696106)
Supplement: Supplementary Table 0 — Topic statement and problem formulation. [file DataSheet_2.zip › DATA sheet 2/Supplementary Table 6.docx]

| Species | D+L pooled WMD | [95% Conf. Interval] | % Weight | I-squared** | p |
| --- | --- | --- | --- | --- | --- |
| Rat | -0.162 | (-0.226, -0.098) | 100 | 91.6% | 0.000 |
| Mouse | / | / | / | / | / |

A

| Exposure Windows | D+L pooled WMD | [95% Conf. Interval] | % Weight | I-squared** | p |
| --- | --- | --- | --- | --- | --- |
| Gestational | -0.118 | (-0.18, -0.055) | 80.27 | 89.5% | 0.000 |
| Lactational | -0.22 | (-0.734, 0.294) | 0.96 | / | / |
| Mature | -0.180 | (-0.401, 0.042) | 15.98 | 95.0% | 0.000 |
| Pubertal | -1.302 | (-2.115, -0.489) | 2.78 | 89.5% | 0.002 |

B

| Dosage Levels | D+L pooled WMD | [95% Conf. Interval] | % Weight | I-squared** | p |
| --- | --- | --- | --- | --- | --- |
| Low | 0.029 | (-0.048, 0.105) | 27.75 | 77.7% | 0.000 |
| Relatively Low | -0.104 | (-0.157, -0.052) | 43.81 | 73.1% | 0.000 |
| Relatively High | -0.274 | (-0.380, -0.169) | 23.47 | 76.6% | 0.000 |
| High | -1.142 | (-1.564, -0.72) | 4.97 | 84.4% | 0.002 |

C
