# Supplementary material for: Animal Toxicology Studies on the Male Reproductive Effects of 2,3,7,8-Tetrachlorodibenzo-p-Dioxin: Data Analysis and Health Effects Evaluation
Source: Front Endocrinol (Lausanne). 2021 Nov 3;12:696106. doi: 10.3389/fendo.2021.696106 (PMC8595279; doi:10.3389/fendo.2021.696106)
Supplement: Supplementary Table 0 — Topic statement and problem formulation. [file DataSheet_2.zip › DATA sheet 2/Supplementary Table 7.docx]

| Species | D+L pooled WMD | [95% Conf. Interval] | % Weight | I-squared** | p |
| --- | --- | --- | --- | --- | --- |
| Rat | 0.001 | (-0.030, 0.033) | 82.55 | 98.0% | 0.000 |
| Mouse | -0.054 | (-0.035, 0.021) | 17.45 | 0.0% | 0.453 |

A

| Exposure Windows | D+L pooled WMD | [95% Conf. Interval] | % Weight | I-squared** | p |
| --- | --- | --- | --- | --- | --- |
| Pubertal | 0.132 | (0.015, 0.248) | 17.97 | 94.9% | 0.000 |
| Pubertal-Mature | 0.042 | (-0.007, 0.091) | 21.07 | 97.4% | 0.000 |
| Pregestational-Pubertal | 0.01 | (-0.001, 0.021) | 24.89 | 0.0% | 0.971 |
| Gestational | -0.034 | (-0.08, 0.012) | 16.32 | 92.0% | 0.000 |
| Mature | -0.246 | (-0.405, -0.087) | 14.29 | 97.2% | 0.000 |
| Lactational | -0.031 | (-0.103, 0.042) | 5.44 | 0.0% | 0.505 |

B

| Dosage Levels | D+L pooled WMD | [95% Conf. Interval] | % Weight | I-squared** | p |
| --- | --- | --- | --- | --- | --- |
| High | 0.132 | (0.015, 0.248) | 17.97 | 94.9% | 0.000 |
| Relatively Low | -0.080 | (-0.157, -0.002) | 23.34 | 98.9% | 0.000 |
| Low | -0.007 | (-0.025, 0.011) | 36.92 | 79.6% | 0.000 |
| Relatively High | -0.033 | (-0.073, 0.006) | 21.77 | 87.0% | 0.000 |

C
