# Supplementary material for: Animal Toxicology Studies on the Male Reproductive Effects of 2,3,7,8-Tetrachlorodibenzo-p-Dioxin: Data Analysis and Health Effects Evaluation
Source: Front Endocrinol (Lausanne). 2021 Nov 3;12:696106. doi: 10.3389/fendo.2021.696106 (PMC8595279; doi:10.3389/fendo.2021.696106)
Supplement: Supplementary Table 0 — Topic statement and problem formulation. [file DataSheet_2.zip › DATA sheet 2/Supplementary Table 8.docx]

| Species | D+L pooled WMD | [95% Conf. Interval] | % Weight | I-squared** | p |
| --- | --- | --- | --- | --- | --- |
| Rat | -0.017 | (-0.05, 0.016) | 100 | 82.9% | 0.000 |
| / | / | / | / | / | / |

A

| Exposure Windows | D+L pooled WMD | [95% Conf. Interval] | % Weight | I-squared** | p |
| --- | --- | --- | --- | --- | --- |
| Gestational | -0.006 | (-0.027, 0.016) | 53.04 | 0.0% | 0.669 |
| Lactational | -0.071 | (-0.185, 0.043) | 3.67 | / | / |
| Pregestational-Lactational | -0.040 | (-0.205, 0.125) | 2.47 | / | / |
| Mature | 0.003 | (-0.021, 0.028)) | 34.69 | 42.1% | 0.125 |
| Pubertal | -0.190 | (-0.225, 0.155) | 6.13 | / | / |

B

| Dosage Levels | D+L pooled WMD | [95% Conf. Interval] | % Weight | I-squared** | p |
| --- | --- | --- | --- | --- | --- |
| Relatively High | -0.018 | (-0.047, 0.011) | 30.84 | 35.3% | 0.172 |
| Low | 0.011 | (-0.014, 0.037) | 32.67 | 0.0% | 0.709 |
| Relatively Low | 0.001 | (-0.024, 0.025) | 30.36 | 0.0% | 0.592 |
| High | -0.190 | (-0.225, -0.155) | 6.13 | / | / |

C
