# Supplementary material for: Animal Toxicology Studies on the Male Reproductive Effects of 2,3,7,8-Tetrachlorodibenzo-p-Dioxin: Data Analysis and Health Effects Evaluation
Source: Front Endocrinol (Lausanne). 2021 Nov 3;12:696106. doi: 10.3389/fendo.2021.696106 (PMC8595279; doi:10.3389/fendo.2021.696106)
Supplement: Supplementary Table 0 — Topic statement and problem formulation. [file DataSheet_2.zip › DATA sheet 2/Supplementary Table 9.docx]

| Species | D+L pooled WMD | [95% Conf. Interval] | % Weight | I-squared** | p |
| --- | --- | --- | --- | --- | --- |
| Rat | -0.036 | (-0.042, -0.030) | 77.25 | 92.3% | 0.000 |
| Mouse | -0.004 | (-0.005, -0.003) | 22.75 | 0.0% | 0.461 |

A

| Exposure Windows | D+L pooled WMD | [95% Conf. Interval] | % Weight | I-squared** | p |
| --- | --- | --- | --- | --- | --- |
| Mature | -0.045 | (-0.060, -0.030) | 16.31 | 81.4% | 0.000 |
| Gestational | -0.025 | (-0.031, -0.020) | 69.41 | 92.6% | 0.000 |
| Pubertal-Mature | -0.025 | (-0.028, -0.023) | 10.18 | 52.6% | 0.061 |
| Lactational | -0.004 | (-0.010, 0.002) | 3.94 | 0% | 0.678 |
| Pubertal | -0.200 | (-0.322, -0.078) | 0.16 | / | / |

B

| Dosage Levels | D+L pooled WMD | [95% Conf. Interval] | % Weight | I-squared** | p |
| --- | --- | --- | --- | --- | --- |
| High | -0.020 | (-0.030, -0.010) | 23.43 | 82.1% | 0.000 |
| Low | -0.011 | (-0.019, -0.002) | 20.50 | 90.7% | 0.000 |
| Relatively Low | -0.037 | (-0.048, -0.027) | 32.19 | 95.3% | 0.000 |
| Relatively High | -0.034 | (-0.042, -0.026) | 23.88 | 92.4% | 0.000 |

C
