# Supplementary material for: Animal Toxicology Studies on the Male Reproductive Effects of 2,3,7,8-Tetrachlorodibenzo-p-Dioxin: Data Analysis and Health Effects Evaluation
Source: Front Endocrinol (Lausanne). 2021 Nov 3;12:696106. doi: 10.3389/fendo.2021.696106 (PMC8595279; doi:10.3389/fendo.2021.696106)
Supplement: Supplementary Figure 1 — Forest plots of overall effects. (A): Overall effect of TCDD and sperm motility (%); (B): Overall effect of TCDD and abnormal sperm (%); (C): Overall effect of TCDD and anogenital distance(mm); (D): Overall effect of TCDD and relative anogenital distance (%body length); (E): Overall effect of TCDD and seminal vesicle weight (g); (F): Overall effect of TCDD and prostate weight (g) [file Supplementary Figure 14.pdf › DATA sheet 1/Supplementary Figure 14.pdf]

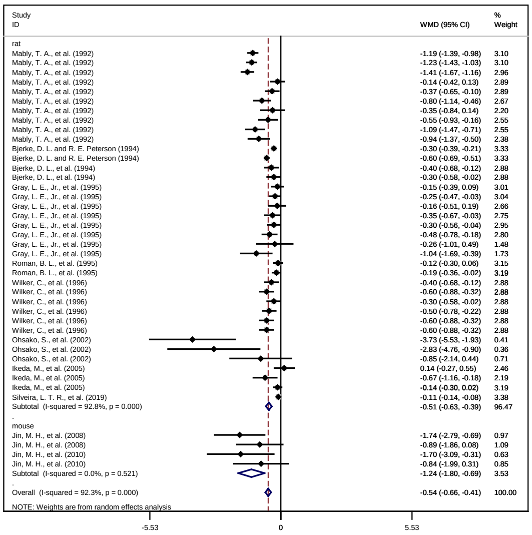
Study
ID
WMD (95% CI)
%
Weight
rat
Mably, T. A., et al. (1992) -1.19 (-1.39, -0.98) 3.10
Mably, T. A., et al. (1992) -1.23 (-1.43, -1.03) 3.10
Mably, T. A., et al. (1992) -1.41 (-1.67, -1.16) 2.96
Mably, T. A., et al. (1992) -0.14 (-0.42, 0.13) 2.89
Mably, T. A., et al. (1992) -0.37 (-0.65, -0.10) 2.89
Mably, T. A., et al. (1992) -0.80 (-1.14, -0.46) 2.67
Mably, T. A., et al. (1992) -0.35 (-0.84, 0.14) 2.20
Mably, T. A., et al. (1992) -0.55 (-0.93, -0.16) 2.55
Mably, T. A., et al. (1992) -1.09 (-1.47, -0.71) 2.55
Mably, T. A., et al. (1992) -0.94 (-1.37, -0.50) 2.38
Bjerke, D. L. and R. E. Peterson (1994) -0.30 (-0.39, -0.21) 3.33
Bjerke, D. L. and R. E. Peterson (1994) -0.60 (-0.69, -0.51) 3.33
Bjerke, D. L., et al. (1994) -0.40 (-0.68, -0.12) 2.88
Bjerke, D. L., et al. (1994) -0.30 (-0.58, -0.02) 2.88
Gray, L. E., Jr., et al. (1995) -0.15 (-0.39, 0.09) 3.01
Gray, L. E., Jr., et al. (1995) -0.25 (-0.47, -0.03) 3.04
Gray, L. E., Jr., et al. (1995) -0.16 (-0.51, 0.19) 2.66
Gray, L. E., Jr., et al. (1995) -0.35 (-0.67, -0.03) 2.75
Gray, L. E., Jr., et al. (1995) -0.30 (-0.56, -0.04) 2.95
Gray, L. E., Jr., et al. (1995) -0.48 (-0.78, -0.18) 2.80
Gray, L. E., Jr., et al. (1995) -0.26 (-1.01, 0.49) 1.48
Gray, L. E., Jr., et al. (1995) -1.04 (-1.69, -0.39) 1.73
Roman, B. L., et al. (1995) -0.12 (-0.30, 0.06) 3.15
Roman, B. L., et al. (1995) -0.19 (-0.36, -0.02) 3.19
Wilker, C., et al. (1996) -0.40 (-0.68, -0.12) 2.88
Wilker, C., et al. (1996) -0.60 (-0.88, -0.32) 2.88
Wilker, C., et al. (1996) -0.30 (-0.58, -0.02) 2.88
Wilker, C., et al. (1996) -0.50 (-0.78, -0.22) 2.88
Wilker, C., et al. (1996) -0.60 (-0.88, -0.32) 2.88
Wilker, C., et al. (1996) -0.60 (-0.88, -0.32) 2.88
Ohsako, S., et al. (2002) -3.73 (-5.53, -1.93) 0.41
Ohsako, S., et al. (2002) -2.83 (-4.76, -0.90) 0.36
Ohsako, S., et al. (2002) -0.85 (-2.14, 0.44) 0.71
Ikeda, M., et al. (2005) 0.14 (-0.27, 0.55) 2.46
Ikeda, M., et al. (2005) -0.67 (-1.16, -0.18) 2.19
Ikeda, M., et al. (2005) -0.14 (-0.30, 0.02) 3.19
Silveira, L. T. R., et al. (2019) -0.11 (-0.14, -0.08) 3.38
Subtotal (I-squared = 92.8%, p = 0.000) -0.51 (-0.63, -0.39) 96.47
mouse
Jin, M. H., et al. (2008) -1.74 (-2.79, -0.69) 0.97
Jin, M. H., et al. (2008) -0.89 (-1.86, 0.08) 1.09
Jin, M. H., et al. (2010) -1.70 (-3.09, -0.31) 0.63
Jin, M. H., et al. (2010) -0.84 (-1.99, 0.31) 0.85
Subtotal (I-squared = 0.0%, p = 0.521) -1.24 (-1.80, -0.69) 3.53
Overall (I-squared = 92.3%, p = 0.000) -0.54 (-0.66, -0.41) 100.00
NOTE: Weights are from random effects analysis
-5.53
0
5.53

A

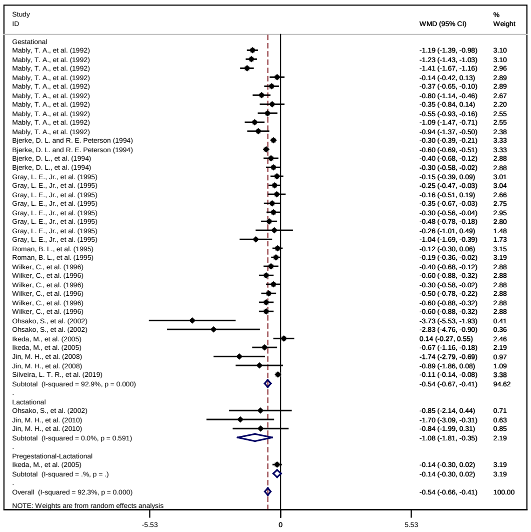
Study
ID
WMD (95% CI)
%
Weight
Gestational
Mably, T. A., et al. (1992) -1.19 (-1.39, -0.98) 3.10
Mably, T. A., et al. (1992) -1.23 (-1.43, -1.03) 3.10
Mably, T. A., et al. (1992) -1.41 (-1.67, -1.16) 2.96
Mably, T. A., et al. (1992) -0.14 (-0.42, 0.13) 2.89
Mably, T. A., et al. (1992) -0.37 (-0.65, -0.10) 2.89
Mably, T. A., et al. (1992) -0.80 (-1.14, -0.46) 2.67
Mably, T. A., et al. (1992) -0.35 (-0.84, 0.14) 2.20
Mably, T. A., et al. (1992) -0.55 (-0.93, -0.16) 2.55
Mably, T. A., et al. (1992) -1.09 (-1.47, -0.71) 2.55
Mably, T. A., et al. (1992) -0.94 (-1.37, -0.50) 2.38
Bjerke, D. L. and R. E. Peterson (1994) -0.30 (-0.39, -0.21) 3.33
Bjerke, D. L. and R. E. Peterson (1994) -0.60 (-0.69, -0.51) 3.33
Bjerke, D. L., et al. (1994) -0.40 (-0.68, -0.12) 2.88
Bjerke, D. L., et al. (1994) -0.30 (-0.58, -0.02) 2.88
Gray, L. E., Jr., et al. (1995) -0.15 (-0.39, 0.09) 3.01
Gray, L. E., Jr., et al. (1995) -0.25 (-0.47, -0.03) 3.04
Gray, L. E., Jr., et al. (1995) -0.16 (-0.51, 0.19) 2.66
Gray, L. E., Jr., et al. (1995) -0.35 (-0.67, -0.03) 2.75
Gray, L. E., Jr., et al. (1995) -0.30 (-0.56, -0.04) 2.95
Gray, L. E., Jr., et al. (1995) -0.48 (-0.78, -0.18) 2.80
Gray, L. E., Jr., et al. (1995) -0.26 (-1.01, 0.49) 1.48
Gray, L. E., Jr., et al. (1995) -1.04 (-1.69, -0.39) 1.73
Roman, B. L., et al. (1995) -0.12 (-0.30, 0.06) 3.15
Roman, B. L., et al. (1995) -0.19 (-0.36, -0.02) 3.19
Wilker, C., et al. (1996) -0.40 (-0.68, -0.12) 2.88
Wilker, C., et al. (1996) -0.60 (-0.88, -0.32) 2.88
Wilker, C., et al. (1996) -0.30 (-0.58, -0.02) 2.88
Wilker, C., et al. (1996) -0.50 (-0.78, -0.22) 2.88
Wilker, C., et al. (1996) -0.60 (-0.88, -0.32) 2.88
Wilker, C., et al. (1996) -0.60 (-0.88, -0.32) 2.88
Ohsako, S., et al. (2002) -3.73 (-5.53, -1.93) 0.41
Ohsako, S., et al. (2002) -2.83 (-4.76, -0.90) 0.36
Ikeda, M., et al. (2005) 0.14 (-0.27, 0.55) 2.46
Ikeda, M., et al. (2005) -0.67 (-1.16, -0.18) 2.19
Jin, M. H., et al. (2008) -1.74 (-2.79, -0.69) 0.97
Jin, M. H., et al. (2008) -0.89 (-1.86, 0.08) 1.09
Silveira, L. T. R., et al. (2019) -0.11 (-0.14, -0.08) 3.38
Subtotal (I-squared = 92.9%, p = 0.000) -0.54 (-0.67, -0.41) 94.62
Lactational
Ohsako, S., et al. (2002) -0.85 (-2.14, 0.44) 0.71
Jin, M. H., et al. (2010) -1.70 (-3.09, -0.31) 0.63
Jin, M. H., et al. (2010) -0.84 (-1.99, 0.31) 0.85
Subtotal (I-squared = 0.0%, p = 0.591) -1.08 (-1.81, -0.35) 2.19
Pregestational-Lactational
Ikeda, M., et al. (2005) -0.14 (-0.30, 0.02) 3.19
Subtotal (I-squared = .%, p = .) -0.14 (-0.30, 0.02) 3.19
Overall (I-squared = 92.3%, p = 0.000) -0.54 (-0.66, -0.41) 100.00
NOTE: Weights are from random effects analysis
-5.53
0
5.53

B

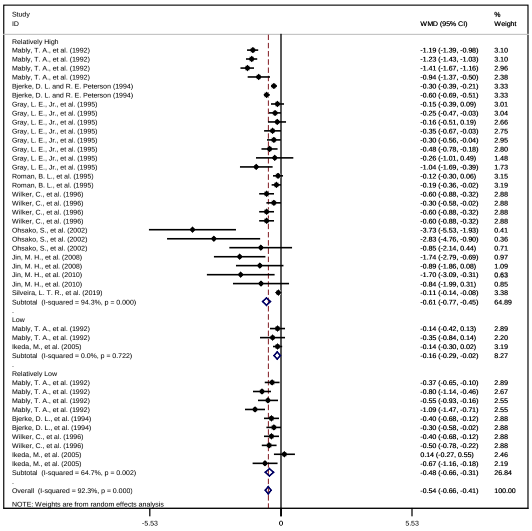
Study
ID
WMD (95% CI)
%
Weight
Relatively High
Mably, T. A., et al. (1992) -1.19 (-1.39, -0.98) 3.10
Mably, T. A., et al. (1992) -1.23 (-1.43, -1.03) 3.10
Mably, T. A., et al. (1992) -1.41 (-1.67, -1.16) 2.96
Mably, T. A., et al. (1992) -0.94 (-1.37, -0.50) 2.38
Bjerke, D. L. and R. E. Peterson (1994) -0.30 (-0.39, -0.21) 3.33
Bjerke, D. L. and R. E. Peterson (1994) -0.60 (-0.69, -0.51) 3.33
Gray, L. E., Jr., et al. (1995) -0.15 (-0.39, 0.09) 3.01
Gray, L. E., Jr., et al. (1995) -0.25 (-0.47, -0.03) 3.04
Gray, L. E., Jr., et al. (1995) -0.16 (-0.51, 0.19) 2.66
Gray, L. E., Jr., et al. (1995) -0.35 (-0.67, -0.03) 2.75
Gray, L. E., Jr., et al. (1995) -0.30 (-0.56, -0.04) 2.95
Gray, L. E., Jr., et al. (1995) -0.48 (-0.78, -0.18) 2.80
Gray, L. E., Jr., et al. (1995) -0.26 (-1.01, 0.49) 1.48
Gray, L. E., Jr., et al. (1995) -1.04 (-1.69, -0.39) 1.73
Roman, B. L., et al. (1995) -0.12 (-0.30, 0.06) 3.15
Roman, B. L., et al. (1995) -0.19 (-0.36, -0.02) 3.19
Wilker, C., et al. (1996) -0.60 (-0.88, -0.32) 2.88
Wilker, C., et al. (1996) -0.30 (-0.58, -0.02) 2.88
Wilker, C., et al. (1996) -0.60 (-0.88, -0.32) 2.88
Wilker, C., et al. (1996) -0.60 (-0.88, -0.32) 2.88
Ohsako, S., et al. (2002) -3.73 (-5.53, -1.93) 0.41
Ohsako, S., et al. (2002) -2.83 (-4.76, -0.90) 0.36
Ohsako, S., et al. (2002) -0.85 (-2.14, 0.44) 0.71
Jin, M. H., et al. (2008) -1.74 (-2.79, -0.69) 0.97
Jin, M. H., et al. (2008) -0.89 (-1.86, 0.08) 1.09
Jin, M. H., et al. (2010) -1.70 (-3.09, -0.31) 0.63
Jin, M. H., et al. (2010) -0.84 (-1.99, 0.31) 0.85
Silveira, L. T. R., et al. (2019) -0.11 (-0.14, -0.08) 3.38
Subtotal (I-squared = 94.3%, p = 0.000) -0.61 (-0.77, -0.45) 64.89
Low
Mably, T. A., et al. (1992) -0.14 (-0.42, 0.13) 2.89
Mably, T. A., et al. (1992) -0.35 (-0.84, 0.14) 2.20
Ikeda, M., et al. (2005) -0.14 (-0.30, 0.02) 3.19
Subtotal (I-squared = 0.0%, p = 0.722) -0.16 (-0.29, -0.02) 8.27
Relatively Low
Mably, T. A., et al. (1992) -0.37 (-0.65, -0.10) 2.89
Mably, T. A., et al. (1992) -0.80 (-1.14, -0.46) 2.67
Mably, T. A., et al. (1992) -0.55 (-0.93, -0.16) 2.55
Mably, T. A., et al. (1992) -1.09 (-1.47, -0.71) 2.55
Bjerke, D. L., et al. (1994) -0.40 (-0.68, -0.12) 2.88
Bjerke, D. L., et al. (1994) -0.30 (-0.58, -0.02) 2.88
Wilker, C., et al. (1996) -0.40 (-0.68, -0.12) 2.88
Wilker, C., et al. (1996) -0.50 (-0.78, -0.22) 2.88
Ikeda, M., et al. (2005) 0.14 (-0.27, 0.55) 2.46
Ikeda, M., et al. (2005) -0.67 (-1.16, -0.18) 2.19
Subtotal (I-squared = 64.7%, p = 0.002) -0.48 (-0.66, -0.31) 26.84
Overall (I-squared = 92.3%, p = 0.000) -0.54 (-0.66, -0.41) 100.00
NOTE: Weights are from random effects analysis
-5.53
0
5.53

C
