# Supplementary material for: Animal Toxicology Studies on the Male Reproductive Effects of 2,3,7,8-Tetrachlorodibenzo-p-Dioxin: Data Analysis and Health Effects Evaluation
Source: Front Endocrinol (Lausanne). 2021 Nov 3;12:696106. doi: 10.3389/fendo.2021.696106 (PMC8595279; doi:10.3389/fendo.2021.696106)
Supplement: Supplementary Figure 1 — Forest plots of overall effects. (A): Overall effect of TCDD and sperm motility (%); (B): Overall effect of TCDD and abnormal sperm (%); (C): Overall effect of TCDD and anogenital distance(mm); (D): Overall effect of TCDD and relative anogenital distance (%body length); (E): Overall effect of TCDD and seminal vesicle weight (g); (F): Overall effect of TCDD and prostate weight (g) [file Supplementary Figure 17.pdf › DATA sheet 1/Supplementary Figure 17.pdf]

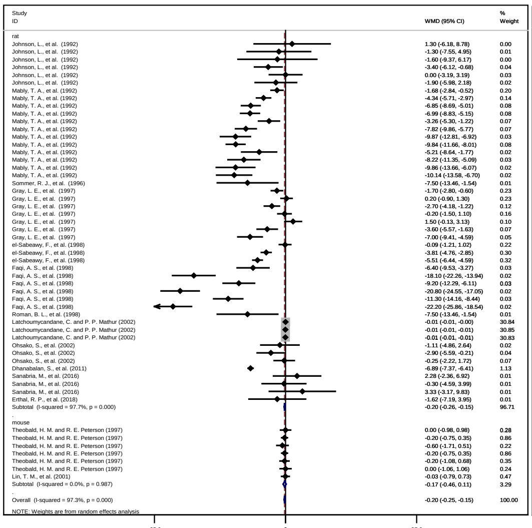
Study ID
WMD (95% CI)
% Weight
rat
Johnson, L., et al. (1992) 1.30 (-6.18, 8.78) 0.00
Johnson, L., et al. (1992) -1.30 (-7.55, 4.95) 0.01
Johnson, L., et al. (1992) -1.60 (-9.37, 6.17) 0.00
Johnson, L., et al. (1992) -3.40 (-6.12, -0.68) 0.04
Johnson, L., et al. (1992) 0.00 (-3.19, 3.19) 0.03
Johnson, L., et al. (1992) -1.90 (-5.98, 2.18) 0.02
Mably, T. A., et al. (1992) -1.68 (-2.84, -0.52) 0.20
Mably, T. A., et al. (1992) -4.34 (-5.71, -2.97) 0.14
Mably, T. A., et al. (1992) -6.85 (-8.69, -5.01) 0.08
Mably, T. A., et al. (1992) -6.99 (-8.83, -5.15) 0.08
Mably, T. A., et al. (1992) -3.26 (-5.30, -1.22) 0.07
Mably, T. A., et al. (1992) -7.82 (-9.86, -5.77) 0.07
Mably, T. A., et al. (1992) -9.87 (-12.81, -6.92) 0.03
Mably, T. A., et al. (1992) -9.84 (-11.66, -8.01) 0.08
Mably, T. A., et al. (1992) -5.21 (-8.64, -1.77) 0.02
Mably, T. A., et al. (1992) -8.22 (-11.35, -5.09) 0.03
Mably, T. A., et al. (1992) -9.86 (-13.66, -6.07) 0.02
Mably, T. A., et al. (1992) -10.14 (-13.58, -6.70) 0.02
Sommer, R. J., et al. (1996) -7.50 (-13.46, -1.54) 0.01
Gray, L. E., et al. (1997) -1.70 (-2.80, -0.60) 0.23
Gray, L. E., et al. (1997) 0.20 (-0.90, 1.30) 0.23
Gray, L. E., et al. (1997) -2.70 (-4.18, -1.22) 0.12
Gray, L. E., et al. (1997) -0.20 (-1.50, 1.10) 0.16
Gray, L. E., et al. (1997) 1.50 (-0.13, 3.13) 0.10
Gray, L. E., et al. (1997) -3.60 (-5.57, -1.63) 0.07
Gray, L. E., et al. (1997) -7.00 (-9.41, -4.59) 0.05
el-Sabeawy, F., et al. (1998) -0.09 (-1.21, 1.02) 0.22
el-Sabeawy, F., et al. (1998) -3.81 (-4.76, -2.85) 0.30
el-Sabeawy, F., et al. (1998) -5.51 (-6.44, -4.59) 0.32
Faqi, A. S., et al. (1998) -6.40 (-9.53, -3.27) 0.03
Faqi, A. S., et al. (1998) -18.10 (-22.26, -13.94) 0.02
Faqi, A. S., et al. (1998) -9.20 (-12.29, -6.11) 0.03
Faqi, A. S., et al. (1998) -20.80 (-24.55, -17.05) 0.02
Faqi, A. S., et al. (1998) -11.30 (-14.16, -8.44) 0.03
Faqi, A. S., et al. (1998) -22.20 (-25.86, -18.54) 0.02
Roman, B. L., et al. (1998) -7.50 (-13.46, -1.54) 0.01
Latchoumycandane, C. and P. P. Mathur (2002) -0.01 (-0.01, -0.00) 30.84
Latchoumycandane, C. and P. P. Mathur (2002) -0.01 (-0.01, -0.01) 30.85
Latchoumycandane, C. and P. P. Mathur (2002) -0.01 (-0.01, -0.01) 30.83
Ohsako, S., et al. (2002) -1.11 (-4.86, 2.64) 0.02
Ohsako, S., et al. (2002) -2.90 (-5.59, -0.21) 0.04
Ohsako, S., et al. (2002) -0.25 (-2.22, 1.72) 0.07
Dhanabalan, S., et al. (2011) -6.89 (-7.37, -6.41) 1.13
Sanabria, M., et al. (2016) 2.28 (-2.36, 6.92) 0.01
Sanabria, M., et al. (2016) -0.30 (-4.59, 3.99) 0.01
Sanabria, M., et al. (2016) 3.33 (-3.17, 9.83) 0.01
Erthal, R. P., et al. (2018) -1.62 (-7.19, 3.95) 0.01
Subtotal (I-squared = 97.7%, p = 0.000) -0.20 (-0.26, -0.15) 96.71
mouse
Theobald, H. M. and R. E. Peterson (1997) 0.00 (-0.98, 0.98) 0.28
Theobald, H. M. and R. E. Peterson (1997) -0.20 (-0.75, 0.35) 0.86
Theobald, H. M. and R. E. Peterson (1997) -0.60 (-1.71, 0.51) 0.22
Theobald, H. M. and R. E. Peterson (1997) -0.20 (-0.75, 0.35) 0.86
Theobald, H. M. and R. E. Peterson (1997) -0.20 (-1.08, 0.68) 0.35
Theobald, H. M. and R. E. Peterson (1997) 0.00 (-1.06, 1.06) 0.24
Lin, T. M., et al. (2001) -0.03 (-0.79, 0.73) 0.47
Subtotal (I-squared = 0.0%, p = 0.987) -0.17 (-0.46, 0.11) 3.29
Overall (I-squared = 97.3%, p = 0.000) -0.20 (-0.25, -0.15) 100.00
NOTE: Weights are from random effects analysis
-25.9
0
25.9

A

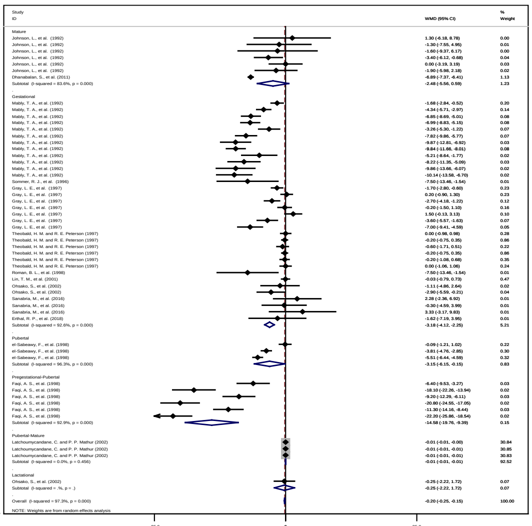

B

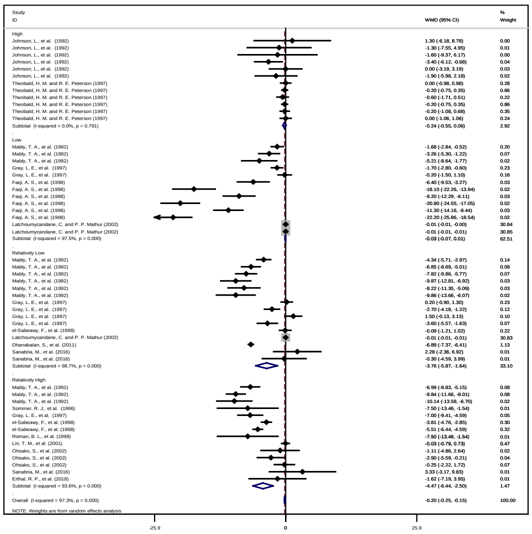

C
