# Supplementary material for: Animal Toxicology Studies on the Male Reproductive Effects of 2,3,7,8-Tetrachlorodibenzo-p-Dioxin: Data Analysis and Health Effects Evaluation
Source: Front Endocrinol (Lausanne). 2021 Nov 3;12:696106. doi: 10.3389/fendo.2021.696106 (PMC8595279; doi:10.3389/fendo.2021.696106)
Supplement: Supplementary Figure 1 — Forest plots of overall effects. (A): Overall effect of TCDD and sperm motility (%); (B): Overall effect of TCDD and abnormal sperm (%); (C): Overall effect of TCDD and anogenital distance(mm); (D): Overall effect of TCDD and relative anogenital distance (%body length); (E): Overall effect of TCDD and seminal vesicle weight (g); (F): Overall effect of TCDD and prostate weight (g) [file Supplementary Figure 18.pdf › DATA sheet 1/Supplementary Figure 18.pdf]

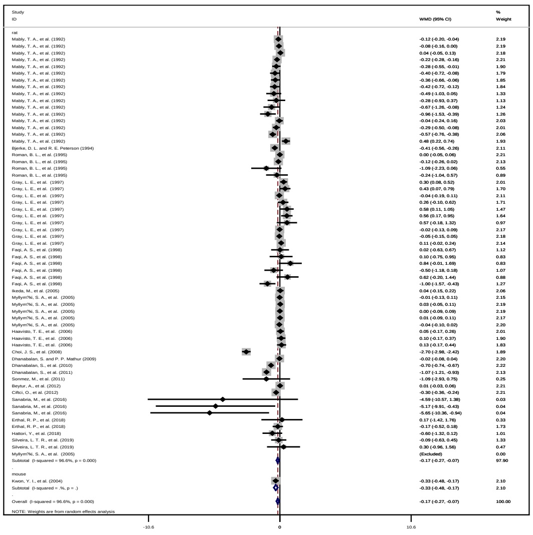

A

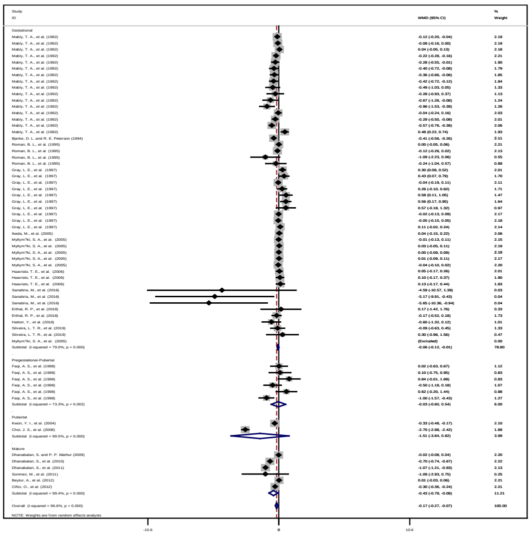

B

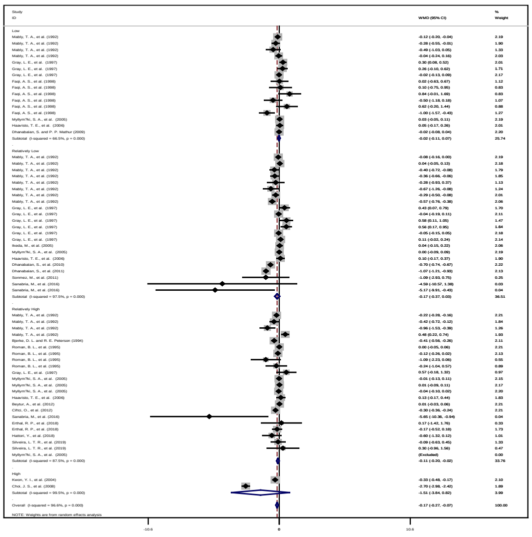

C
