# Supplementary material for: Animal Toxicology Studies on the Male Reproductive Effects of 2,3,7,8-Tetrachlorodibenzo-p-Dioxin: Data Analysis and Health Effects Evaluation
Source: Front Endocrinol (Lausanne). 2021 Nov 3;12:696106. doi: 10.3389/fendo.2021.696106 (PMC8595279; doi:10.3389/fendo.2021.696106)
Supplement: Supplementary Figure 1 — Forest plots of overall effects. (A): Overall effect of TCDD and sperm motility (%); (B): Overall effect of TCDD and abnormal sperm (%); (C): Overall effect of TCDD and anogenital distance(mm); (D): Overall effect of TCDD and relative anogenital distance (%body length); (E): Overall effect of TCDD and seminal vesicle weight (g); (F): Overall effect of TCDD and prostate weight (g) [file Supplementary Figure 6.pdf › DATA sheet 1/Supplementary Figure 6.pdf]

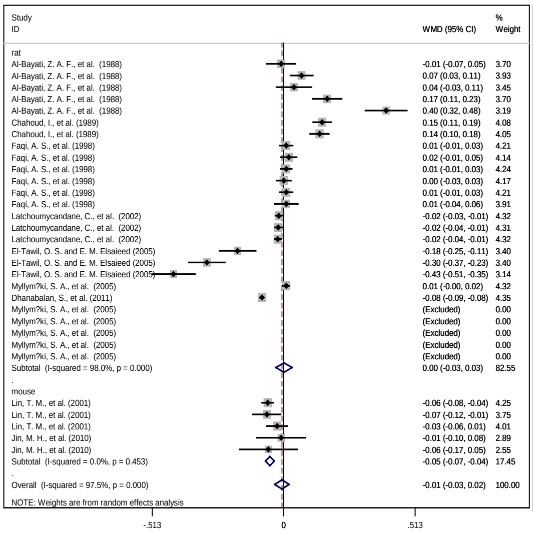
Study
ID
WMD (95% CI)
%
Weight
rat
Al-Bayati, Z. A. F., et al. (1988) -0.01 (-0.07, 0.05) 3.70
Al-Bayati, Z. A. F., et al. (1988) 0.07 (0.03, 0.11) 3.93
Al-Bayati, Z. A. F., et al. (1988) 0.04 (-0.03, 0.11) 3.45
Al-Bayati, Z. A. F., et al. (1988) 0.17 (0.11, 0.23) 3.70
Al-Bayati, Z. A. F., et al. (1988) 0.40 (0.32, 0.48) 3.19
Chahoud, I., et al. (1989) 0.15 (0.11, 0.19) 4.08
Chahoud, I., et al. (1989) 0.14 (0.10, 0.18) 4.05
Faqi, A. S., et al. (1998) 0.01 (-0.01, 0.03) 4.21
Faqi, A. S., et al. (1998) 0.02 (-0.01, 0.05) 4.14
Faqi, A. S., et al. (1998) 0.01 (-0.01, 0.03) 4.24
Faqi, A. S., et al. (1998) 0.00 (-0.03, 0.03) 4.17
Faqi, A. S., et al. (1998) 0.01 (-0.01, 0.03) 4.21
Faqi, A. S., et al. (1998) 0.01 (-0.04, 0.06) 3.91
Latchoumycandane, C., et al. (2002) -0.02 (-0.03, -0.01) 4.32
Latchoumycandane, C., et al. (2002) -0.02 (-0.04, -0.01) 4.31
Latchoumycandane, C., et al. (2002) -0.02 (-0.04, -0.01) 4.32
El-Tawil, O. S. and E. M. Elsaieed (2005) -0.18 (-0.25, -0.11) 3.40
El-Tawil, O. S. and E. M. Elsaieed (2005) -0.30 (-0.37, -0.23) 3.40
El-Tawil, O. S. and E. M. Elsaieed (2005) -0.43 (-0.51, -0.35) 3.14
Myllym?ki, S. A., et al. (2005) 0.01 (-0.00, 0.02) 4.32
Dhanabalan, S., et al. (2011) -0.08 (-0.09, -0.08) 4.35
Myllym?ki, S. A., et al. (2005) (Excluded) 0.00
Myllym?ki, S. A., et al. (2005) (Excluded) 0.00
Myllym?ki, S. A., et al. (2005) (Excluded) 0.00
Myllym?ki, S. A., et al. (2005) (Excluded) 0.00
Myllym?ki, S. A., et al. (2005) (Excluded) 0.00
Subtotal (I-squared = 98.0%, p = 0.000) 0.00 (-0.03, 0.03) 82.55
.
mouse
Lin, T. M., et al. (2001) -0.06 (-0.08, -0.04) 4.25
Lin, T. M., et al. (2001) -0.07 (-0.12, -0.01) 3.75
Lin, T. M., et al. (2001) -0.03 (-0.06, 0.01) 4.01
Jin, M. H., et al. (2010) -0.01 (-0.10, 0.08) 2.89
Jin, M. H., et al. (2010) -0.06 (-0.17, 0.05) 2.55
Subtotal (I-squared = 0.0%, p = 0.453) -0.05 (-0.07, -0.04) 17.45
.
Overall (I-squared = 97.5%, p = 0.000) -0.01 (-0.03, 0.02) 100.00
NOTE: Weights are from random effects analysis
-.513
0
.513

A

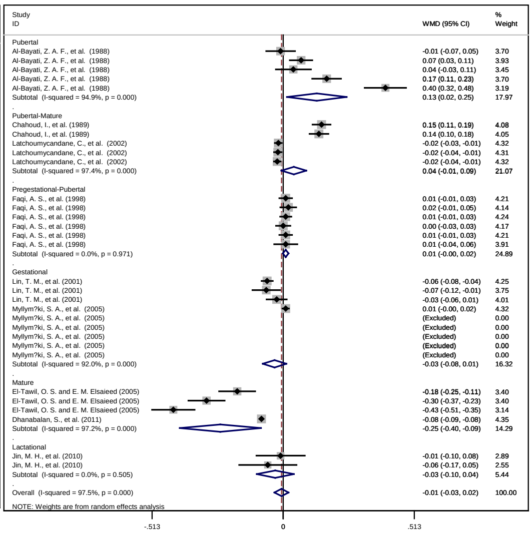
Study
ID
WMD (95% CI)
%
Weight
Pubertal
Al-Bayati, Z. A. F., et al. (1988) -0.01 (-0.07, 0.05) 3.70
Al-Bayati, Z. A. F., et al. (1988) 0.07 (0.03, 0.11) 3.93
Al-Bayati, Z. A. F., et al. (1988) 0.04 (-0.03, 0.11) 3.45
Al-Bayati, Z. A. F., et al. (1988) 0.17 (0.11, 0.23) 3.70
Al-Bayati, Z. A. F., et al. (1988) 0.40 (0.32, 0.48) 3.19
Subtotal (I-squared = 94.9%, p = 0.000) 0.13 (0.02, 0.25) 17.97
.
Pubertal-Mature
Chahoud, I., et al. (1989) 0.15 (0.11, 0.19) 4.08
Chahoud, I., et al. (1989) 0.14 (0.10, 0.18) 4.05
Latchoumycandane, C., et al. (2002) -0.02 (-0.03, -0.01) 4.32
Latchoumycandane, C., et al. (2002) -0.02 (-0.04, -0.01) 4.31
Latchoumycandane, C., et al. (2002) -0.02 (-0.04, -0.01) 4.32
Subtotal (I-squared = 97.4%, p = 0.000) 0.04 (-0.01, 0.09) 21.07
.
Pregestational-Pubertal
Faqi, A. S., et al. (1998) 0.01 (-0.01, 0.03) 4.21
Faqi, A. S., et al. (1998) 0.02 (-0.01, 0.05) 4.14
Faqi, A. S., et al. (1998) 0.01 (-0.01, 0.03) 4.24
Faqi, A. S., et al. (1998) 0.00 (-0.03, 0.03) 4.17
Faqi, A. S., et al. (1998) 0.01 (-0.01, 0.03) 4.21
Faqi, A. S., et al. (1998) 0.01 (-0.04, 0.06) 3.91
Subtotal (I-squared = 0.0%, p = 0.971) 0.01 (-0.00, 0.02) 24.89
.
Gestational
Lin, T. M., et al. (2001) -0.06 (-0.08, -0.04) 4.25
Lin, T. M., et al. (2001) -0.07 (-0.12, -0.01) 3.75
Lin, T. M., et al. (2001) -0.03 (-0.06, 0.01) 4.01
Myllym?ki, S. A., et al. (2005) 0.01 (-0.00, 0.02) 4.32
Myllym?ki, S. A., et al. (2005) (Excluded) 0.00
Myllym?ki, S. A., et al. (2005) (Excluded) 0.00
Myllym?ki, S. A., et al. (2005) (Excluded) 0.00
Myllym?ki, S. A., et al. (2005) (Excluded) 0.00
Myllym?ki, S. A., et al. (2005) (Excluded) 0.00
Subtotal (I-squared = 92.0%, p = 0.000) -0.03 (-0.08, 0.01) 16.32
.
Mature
El-Tawil, O. S. and E. M. Elsaieed (2005) -0.18 (-0.25, -0.11) 3.40
El-Tawil, O. S. and E. M. Elsaieed (2005) -0.30 (-0.37, -0.23) 3.40
El-Tawil, O. S. and E. M. Elsaieed (2005) -0.43 (-0.51, -0.35) 3.14
Dhanabalan, S., et al. (2011) -0.08 (-0.09, -0.08) 4.35
Subtotal (I-squared = 97.2%, p = 0.000) -0.25 (-0.40, -0.09) 14.29
.
Lactational
Jin, M. H., et al. (2010) -0.01 (-0.10, 0.08) 2.89
Jin, M. H., et al. (2010) -0.06 (-0.17, 0.05) 2.55
Subtotal (I-squared = 0.0%, p = 0.505) -0.03 (-0.10, 0.04) 5.44
.
Overall (I-squared = 97.5%, p = 0.000) -0.01 (-0.03, 0.02) 100.00
NOTE: Weights are from random effects analysis
-.513
0
.513

B

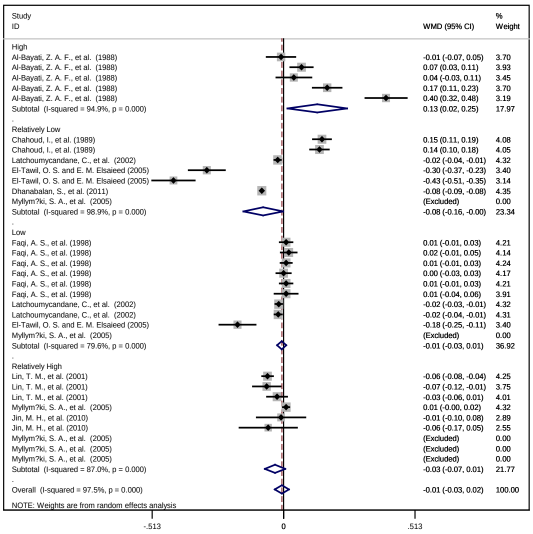
Study
ID
WMD (95% CI)
%
Weight
High
Al-Bayati, Z. A. F., et al. (1988) -0.01 (-0.07, 0.05) 3.70
Al-Bayati, Z. A. F., et al. (1988) 0.07 (0.03, 0.11) 3.93
Al-Bayati, Z. A. F., et al. (1988) 0.04 (-0.03, 0.11) 3.45
Al-Bayati, Z. A. F., et al. (1988) 0.17 (0.11, 0.23) 3.70
Al-Bayati, Z. A. F., et al. (1988) 0.40 (0.32, 0.48) 3.19
Subtotal (I-squared = 94.9%, p = 0.000) 0.13 (0.02, 0.25) 17.97
.
Relatively Low
Chahoud, I., et al. (1989) 0.15 (0.11, 0.19) 4.08
Chahoud, I., et al. (1989) 0.14 (0.10, 0.18) 4.05
Latchoumycandane, C., et al. (2002) -0.02 (-0.04, -0.01) 4.32
El-Tawil, O. S. and E. M. Elsaieed (2005) -0.30 (-0.37, -0.23) 3.40
El-Tawil, O. S. and E. M. Elsaieed (2005) -0.43 (-0.51, -0.35) 3.14
Dhanabalan, S., et al. (2011) -0.08 (-0.09, -0.08) 4.35
Myllym?ki, S. A., et al. (2005) (Excluded) 0.00
Subtotal (I-squared = 98.9%, p = 0.000) -0.08 (-0.16, -0.00) 23.34
.
Low
Faqi, A. S., et al. (1998) 0.01 (-0.01, 0.03) 4.21
Faqi, A. S., et al. (1998) 0.02 (-0.01, 0.05) 4.14
Faqi, A. S., et al. (1998) 0.01 (-0.01, 0.03) 4.24
Faqi, A. S., et al. (1998) 0.00 (-0.03, 0.03) 4.17
Faqi, A. S., et al. (1998) 0.01 (-0.01, 0.03) 4.21
Faqi, A. S., et al. (1998) 0.01 (-0.04, 0.06) 3.91
Latchoumycandane, C., et al. (2002) -0.02 (-0.03, -0.01) 4.32
Latchoumycandane, C., et al. (2002) -0.02 (-0.04, -0.01) 4.31
El-Tawil, O. S. and E. M. Elsaieed (2005) -0.18 (-0.25, -0.11) 3.40
Myllym?ki, S. A., et al. (2005) (Excluded) 0.00
Subtotal (I-squared = 79.6%, p = 0.000) -0.01 (-0.03, 0.01) 36.92
.
Relatively High
Lin, T. M., et al. (2001) -0.06 (-0.08, -0.04) 4.25
Lin, T. M., et al. (2001) -0.07 (-0.12, -0.01) 3.75
Lin, T. M., et al. (2001) -0.03 (-0.06, 0.01) 4.01
Myllym?ki, S. A., et al. (2005) 0.01 (-0.00, 0.02) 4.32
Jin, M. H., et al. (2010) -0.01 (-0.10, 0.08) 2.89
Jin, M. H., et al. (2010) -0.06 (-0.17, 0.05) 2.55
Myllym?ki, S. A., et al. (2005) (Excluded) 0.00
Myllym?ki, S. A., et al. (2005) (Excluded) 0.00
Myllym?ki, S. A., et al. (2005) (Excluded) 0.00
Subtotal (I-squared = 87.0%, p = 0.000) -0.03 (-0.07, 0.01) 21.77
.
Overall (I-squared = 97.5%, p = 0.000) -0.01 (-0.03, 0.02) 100.00
NOTE: Weights are from random effects analysis
-.513
0
.513

C
